# Supplementary material for: A protein interactome for the last eukaryotic common ancestor illuminates the biochemical basis of modern genetic diseases
Source: Cell Genom. 2026 May 27;6(6):101254. doi: 10.1016/j.xgen.2026.101254 (PMC13261697; doi:10.1016/j.xgen.2026.101254)
Supplement: Document S1. Figures S1–S8 [file mmc1.pdf]

Cell Genomics, Volume *6*

Supplemental information

A protein interactome for the last eukaryotic common ancestor illuminates the biochemical basis of modern genetic diseases

Rachael M. Cox, Ophelia Papoulas, Shirlee Shril, Chanjae Lee, Tynan P. Gardner, Zoya T. Ansari, Anna M. Battenhouse, Muyoung Lee, Kevin Drew, Claire D. McWhite, David Yang, Janelle C. Leggere, Dannie Durand, Friedhelm Hildebrandt, John B. Wallingford, and Edward M. Marcotte

SUPPLEMENTAL FIGURES AND LEGENDS

Figure S1. Support for LECA OGs from Wagner parsimony and phylogenetic reconciliation, Related to Figure 1. All OGs presented are supported as present in LECA by at least Dollo parsimony. We further looked for agreement among multiple independent approaches as a robustness test; pathway and gene-level interpretations in the paper are restricted to the multiply-supported subset, with detailed gene-level results provided in **Table S1. (A)** 64% of all LECA OGs supported by Dollo Parsimony are also supported by Wagner or phylogenetic reconciliation with transfers. **(B)** 98% of the core set of 3,193 OGs in the LECA interactome are also supported by Wagner or phylogenetic reconciliation with transfers. For both plots: blue, LECA OGs also supported by Wagner and/or phylogenetic reconciliation with transfers; dark gray, LECA OGs only supported by Dollo parsimony; orange, LECA OGs supported by Dollo parsimony for which phylogenetic reconciliation was not calculated; yellow, LECA OGs supported by Dollo and reconciled as Eukaryotes not Excavata.

Figure S2. Phylogenetic analysis of the reviewed UniProt database by subcellular localization, Related to Figure 1. Limitations to available annotations are evident in an analysis of the UniProt protein database across species, where reviewed proteins have assigned subcellular localizations likely present in the last eukaryotic common ancestor. **(A)** Light gray, total number of reviewed UniProt proteins by UniProt SL term; dark gray, total number of unique eukaryotic OGs assigned to UniProt proteins by UniProt SL term. **(B)** Phylogenetic representation of the proteins sourced from UniProt by UniProt SL term. **(C)** The percentage of eukaryotic orthologous groups (euNOGs) that trace back to LECA by UniProt SL term.

Figure S3. Homozygosity mapping and verification of EFHC2 mutation in individual A4237-22, Related to Figure 6. (A) Homozygosity mapping depicts a homozygosity of 4.4 Mb and confirms the reported non-consanguinity of the parents. **(B)** Chromatograms obtained by direct sequencing of PCR products reveal a homozygous substitution of C for T in exon 4 of the *EFHC2* gene in A4237-22.

Figure S4. Guilt-by-association in the LECA interactome predicts loss-of-function phenotypes in mutant (A,B) yeast and (C) *Chlamydomonas*, Related to Figure 7. Performance in panels **A** and **C** was quantified using the area under receiver operating characteristic curves (AUROC) for leave-one-out cross-validated predictions of known phenotype-linked genes (light blue) versus random associations (yellow), calculated as for Figure 7A and with predictive performance being roughly comparable to the prediction of the human disease gene sets in Figure 7A. Panel **(B)** shows the relevant LECA PPI networks for genes associated with the two labeled yeast phenotypes.

Figure S5. Reference species tree illustration generated by the Interactive Tree of Life for most of the Quest for Orthologs benchmark species (147/156) used in the Dollo and Wagner parsimony analyses, Related to STAR Methods. Branch lengths are not to scale. Major supergroups are highlighted across the tree. Prokaryotic groups include Bacteria (gray) and Archaea (yellow). Eukaryotic groups include Excavata (light blue), Archaeplastida (green), TSAR (purple), and Amorphea (red).

Figure S6. Illustration of transitive closure for grouping gold standard protein complexes into supergroups, Related to STAR Methods.

Figure S7. Illustration of group-based k-fold (in this example, k=3) cross-validation for protein-protein interactions, Related to STAR Methods.

Figure S8. Model selection and optimization, Related to STAR Methods. (A) Precision-recall curves for three different algorithms, varying the number of "top" most important features used as input (duplicate panel to Figure 3B). Feature importance is defined per algorithm, ranked by either the absolute value of coefficients for linear models (LinearSVC, SGDClassifier) or the Gini index (ExtraTreesClassifier). (B) The number of pairwise protein-protein interactions (PPIs) within a 10% FDR threshold for each model. Black stars (★) denote the final models used as input to a community detection algorithm to define protein complexes. (C) The number of unique proteins that have at least one interaction scored within a 10% FDR threshold for each model.

Figure S1.

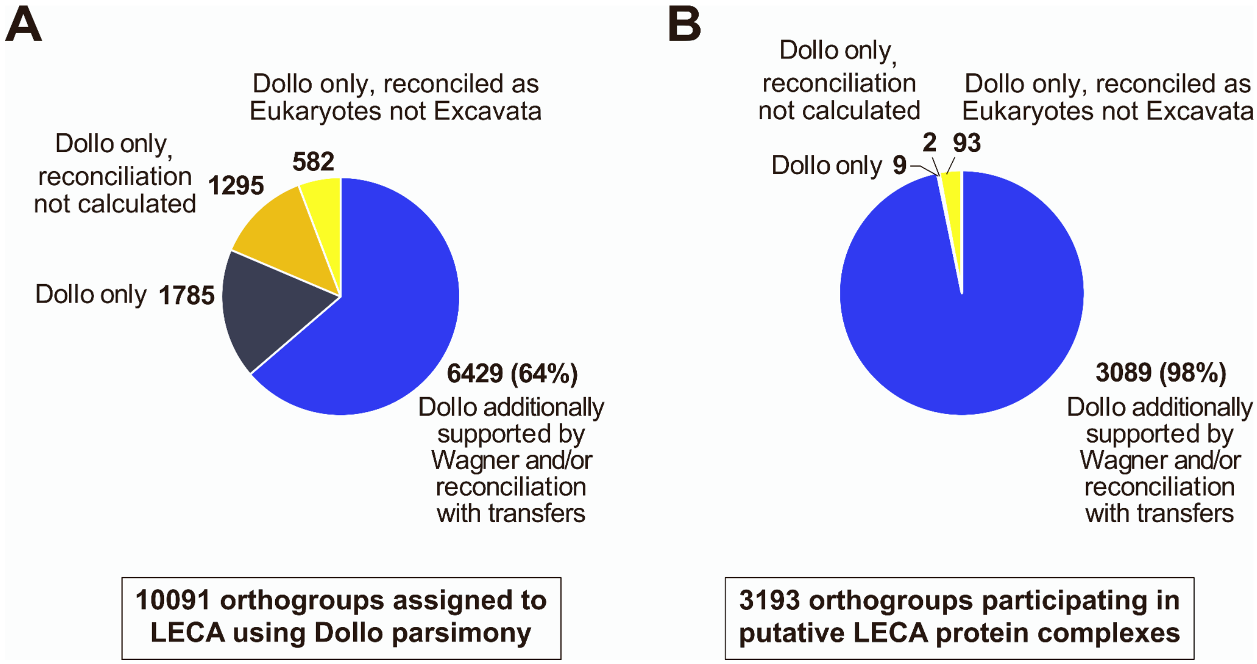
A
Dollo only, reconciled as Eukaryotes not Excavata
582
Dollo only, reconciliation not calculated
1295
Dollo only 1785
6429 (64%)
Dollo additionally supported by Wagner and/or reconciliation with transfers
10091 orthogroups assigned to LECA using Dollo parsimony
B
Dollo only, reconciliation not calculated
Dollo only, reconciled as Eukaryotes not Excavata
2
93
Dollo only 9
3089 (98%)
Dollo additionally supported by Wagner and/or reconciliation with transfers
3193 orthogroups participating in putative LECA protein complexes

Figure S2.

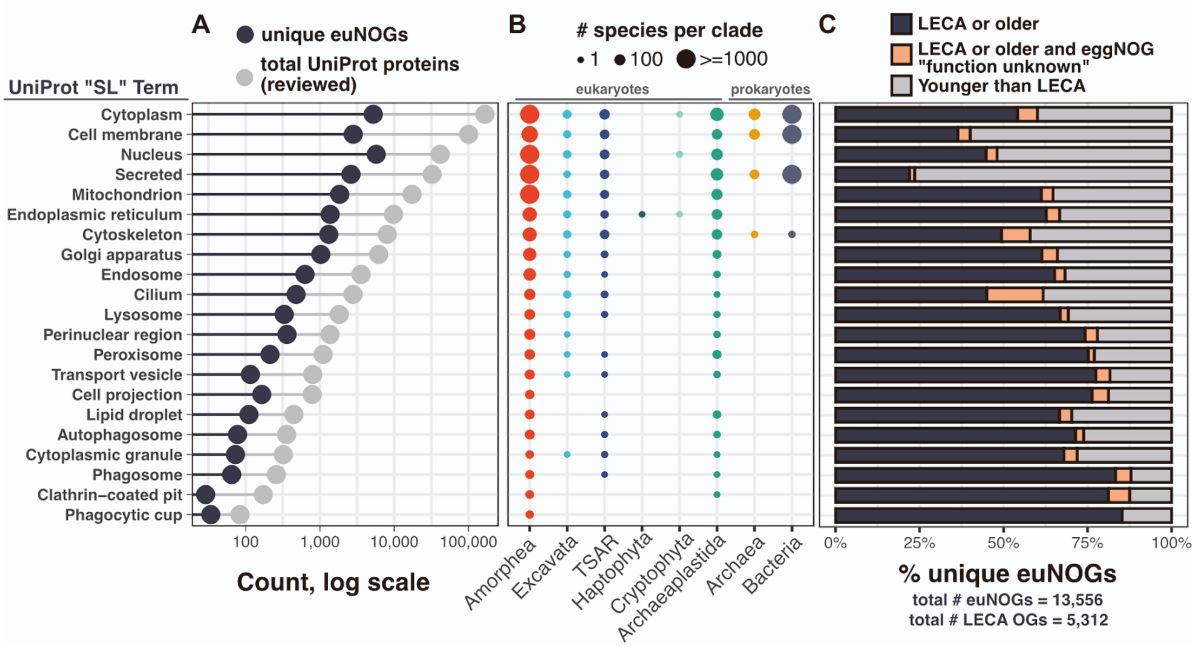
A
unique euNOGs
total UniProt proteins (reviewed)
UniProt "SL" Term
Cytoplasm
Cell membrane
Nucleus
Secreted
Mitochondrion
Endoplasmic reticulum
Cytoskeleton
Golgi apparatus
Endosome
Cilium
Lysosome
Perinuclear region
Peroxisome
Transport vesicle
Cell projection
Lipid droplet
Autophagosome
Cytoplasmic granule
Phagosome
Clathrin–coated pit
Phagocytic cup
100
1,000
10,000
100,000
Count, log scale
B
species per clade
1
100
>=1000
eukaryotes
prokaryotes
Amorphea
Excavata
TSAR
Haptophyta
Cryptophyta
Archaeaplastida
Archaea
Bacteria
C
LECA or older
LECA or older and eggNOG "function unknown"
Younger than LECA
0%
25%
50%
75%
100%
% unique euNOGs
total # euNOGs = 13,556
total # LECA OGs = 5,312

Figure S3.

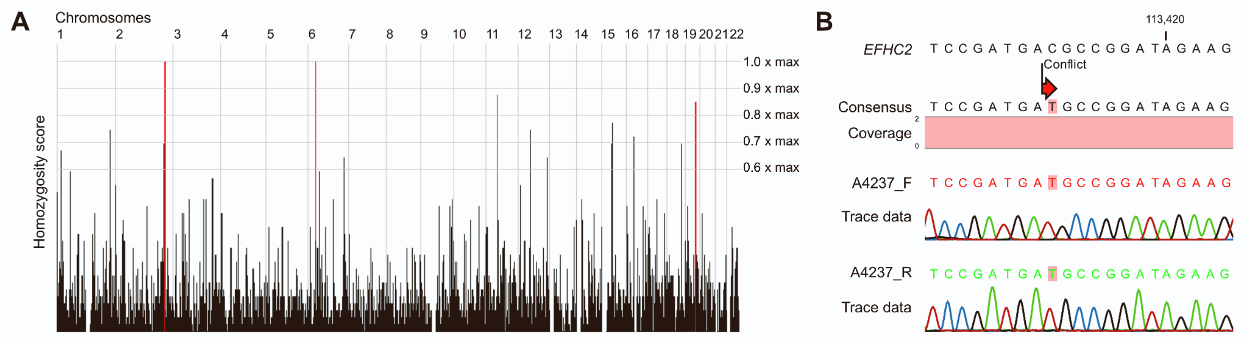
A
Chromosomes
1 2 3 4 5 6 7 8 9 10 11 12 13 14 15 16 17 18 19 20 21 22
1.0 x max
0.9 x max
0.8 x max
0.7 x max
0.6 x max
Homozygosity score
B
113,420
EFHC2
T C C G A T G A C G C C G G A T A G A A G
Conflict
Consensus
T C C G A T G A T G C C G G A T A G A A G
Coverage
A4237_F
T C C G A T G A T G C C G G A T A G A A G
Trace data
A4237_R
T C C G A T G A T G C C G G A T A G A A G
Trace data

Figure S4.

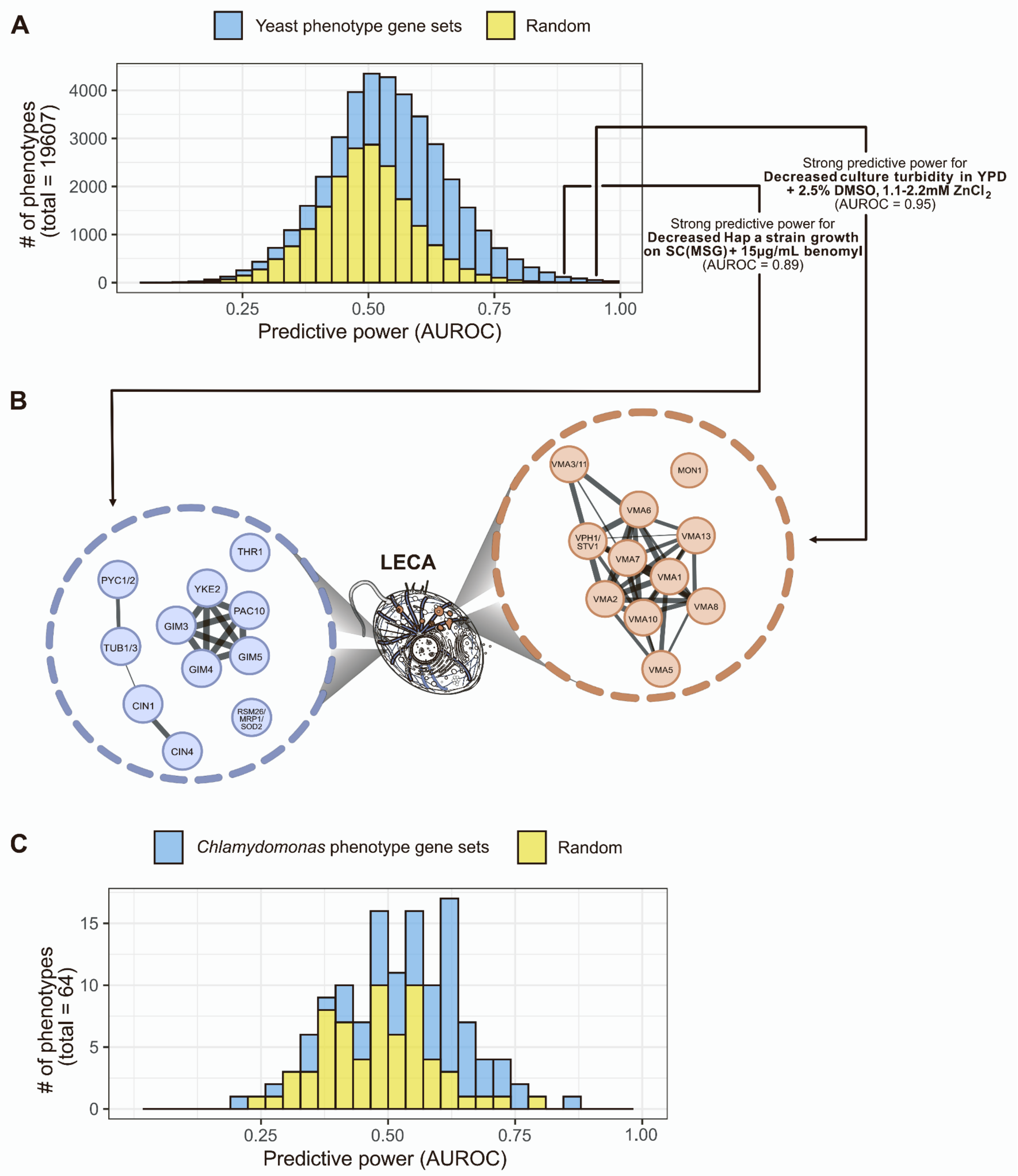
A
Yeast phenotype gene sets
Random
# of phenotypes (total = 19607)
4000
3000
2000
1000
0
0.25
0.50
0.75
1.00
Predictive power (AUROC)
Strong predictive power for
Decreased culture turbidity in YPD + 2.5% DMSO, 1.1-2.2mM ZnCl2
(AUROC = 0.95)
Strong predictive power for
Decreased Hap a strain growth on SC(MSG) + 15μg/mL benomyl
(AUROC = 0.89)
B
LECA
THR1
PYC1/2
YKE2
PAC10
GIM3
GIM5
GIM4
TUB1/3
CIN1
CIN4
RSM26/ MRP1/ SOD2
VMA3/11
MON1
VMA6
VPH1/ STV1
VMA13
VMA7
VMA1
VMA2
VMA8
VMA10
VMA5
C
Chlamydomonas phenotype gene sets
Random
# of phenotypes (total = 64)
15
10
5
0
0.25
0.50
0.75
1.00
Predictive power (AUROC)

Figure S5.

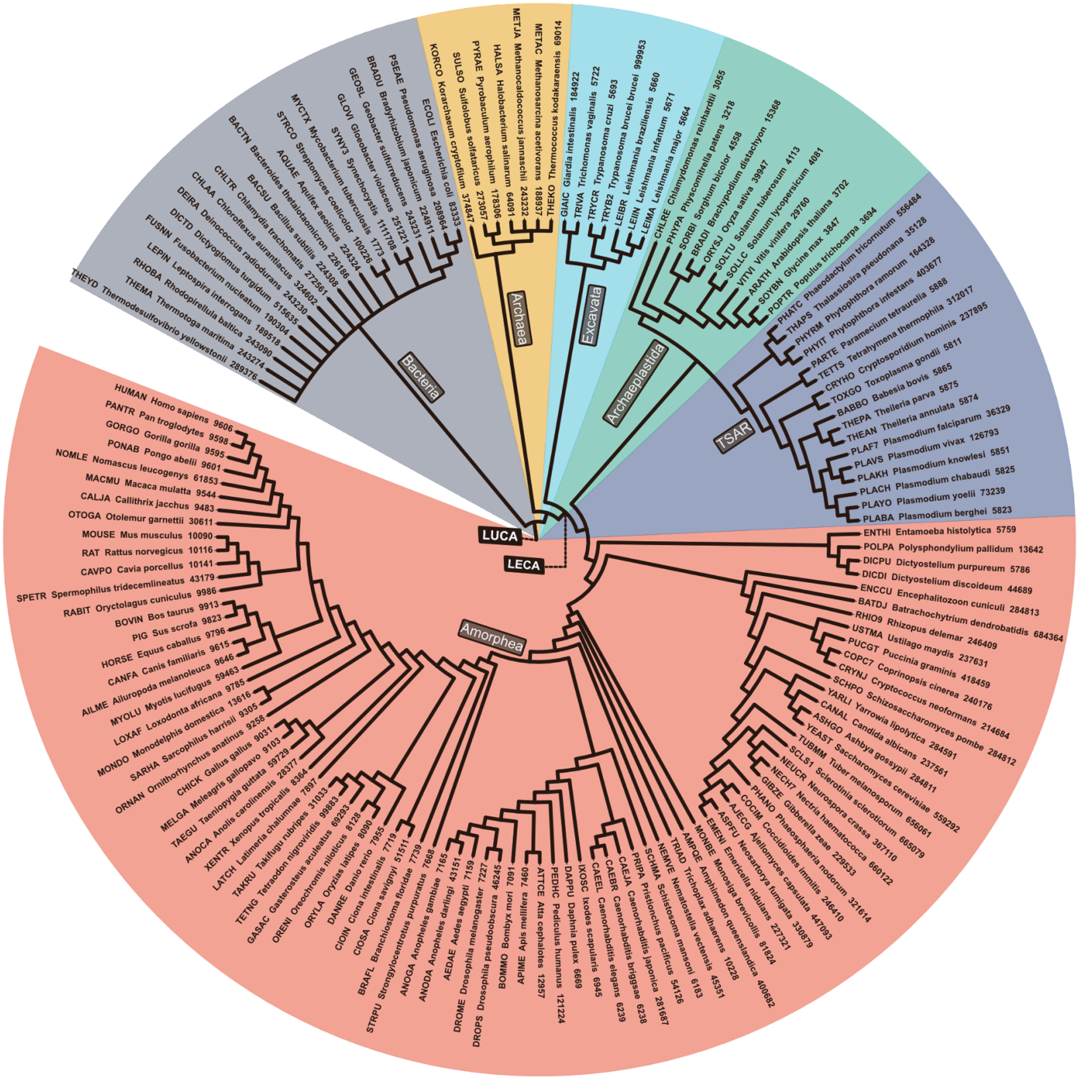
LUCA
LECA
Bacteria
Archaea
Excavata
Archaeplastida
TSAR
Amorphea
THEYD Thermodesulfovibrio yellowstonii 289376
THEMA Thermotoga maritima 243274
RHOBA Rhodopirellula baltica 243090
LEPIN Leptospira interrogans 189518
FUSNN Fusobacterium nucleatum 190304
DICTD Dictyoglomus turgidum 515635
DEIRA Deinococcus radiodurans 243230
CHLAA Chloroflexus aurantiacus 324602
CHLTR Chlamydia trachomatis 272561
BACTN Bacteroides thetaiotaomicron 226186
BACSU Bacillus subtilis 224308
AQUAE Aquifex aeolicus 224324
STRCO Streptomyces coelicolor 100226
MYCTX Mycobacterium tuberculosis 1773
SYNY3 Synechocystis 1111708
GLOVI Gloeobacter violaceus 251221
GEOSL Geobacter sulfurreducens 243231
BRADU Bradyrhizobium japonicum 224911
PSEAE Pseudomonas aeruginosa 208964
ECOLI Escherichia coli 83333
KORCO Korarchaeum cryptofilum 374847
SULSO Sulfolobus solfataricus 273057
PYRAE Pyrobaculum aerophilum 178306
HALSA Halobacterium salinarum 64091
METJA Methanocaldococcus jannaschii 243232
METAC Methanosarcina acetivorans 188937
THEKO Thermococcus kodakarensis 69014
GIAIC Giardia intestinalis 184922
TRIVA Trichomonas vaginalis 5722
TRYCR Trypanosoma cruzi 5693
TRYB2 Trypanosoma brucei brucei 999953
LEIBR Leishmania braziliensis 5660
LEIIN Leishmania infantum 5671
LEIMA Leishmania major 5664
CHLRE Chlamydomonas reinhardtii 3055
PHYPA Physcomitrella patens 3218
SORBI Sorghum bicolor 4558
BRADI Brachypodium distachyon 15368
ORYSJ Oryza sativa 39947
SOLTU Solanum tuberosum 4113
SOLLC Solanum lycopersicum 4081
VITVI Vitis vinifera 29760
ARATH Arabidopsis thaliana 3702
SOYBN Glycine max 3847
POPTR Populus trichocarpa 3694
PHATC Phaeodactylum tricornutum 556484
THAPS Thalassiosira pseudonana 35128
PHYRM Phytophthora ramorum 164328
PHYIT Phytophthora infestans 403677
PARTE Paramecium tetraurelia 5888
TETTS Tetrahymena thermophila 312017
CRYHO Cryptosporidium hominis 237895
TOXGO Toxoplasma gondii 5811
BABBO Babesia bovis 5865
THEPA Theileria parva 5875
THEAN Theileria annulata 5874
PLAF7 Plasmodium falciparum 36329
PLAVS Plasmodium vivax 126793
PLAKH Plasmodium knowlesi 5851
PLACH Plasmodium chabaudi 5825
PLAYO Plasmodium yoelii 73239
PLABA Plasmodium berghei 5823
ENTHI Entamoeba histolytica 5759
POLPA Polysphondylium pallidum 13642
DICPU Dictyostelium purpureum 5786
DICDI Dictyostelium discoideum 44689
ENCCU Encephalitozoon cuniculi 284813
BATDJ Batrachochytrium dendrobatidis 684364
RHIO9 Rhizopus delemar 246409
USTMA Ustilago maydis 237631
PUCGT Puccinia graminis 418459
COPC7 Coprinopsis cinerea 240176
CRYNJ Cryptococcus neoformans 214684
SCHPO Schizosaccharomyces pombe 284812
YARLI Yarrowia lipolytica 284591
CANAL Candida albicans 237561
ASHGO Ashbya gossypii 284811
YEAST Saccharomyces cerevisiae 559292
TUBMM Tuber melanosporum 656061
SCLS1 Sclerotinia sclerotiorum 665079
NEUCR Neurospora crassa 367110
NECH7 Nectria haematococca 660122
GIBZE Gibberella zeae 229533
PHANO Phaeosphaeria nodorum 321614
COCIM Coccidioides immitis 246410
AJECG Ajellomyces capsulatus 447093
ASPFU Neosartorya fumigata 330879
EMENI Emericella nidulans 227321
MONBE Monosiga brevicollis 81824
AMPQE Amphimedon queenslandica 400682
TRIAD Trichoplax adhaerens 10228
NEMVE Nematostella vectensis 45351
SCHMA Schistosoma mansoni 6183
PRIPA Pristionchus pacificus 54126
CAEJA Caenorhabditis japonica 281687
CAEBR Caenorhabditis briggsae 6238
CAEEL Caenorhabditis elegans 6239
IXOSC Ixodes scapularis 6945
DAPPU Daphnia pulex 6669
PEDHC Pediculus humanus 121224
ATTCE Atta cephalotes 12957
APIME Apis mellifera 7460
BOMMO Bombyx mori 7091
DROPS Drosophila pseudoobscura 46245
DROME Drosophila melanogaster 7227
AEDAE Aedes aegypti 7159
ANODA Anopheles darlingi 43151
ANOGA Anopheles gambiae 7165
STRPU Strongylocentrotus purpuratus 7668
BRAFL Branchiostoma floridae 7739
CIOSA Ciona savignyi 51511
CIOIN Ciona intestinalis 7719
DANRE Danio rerio 7955
ORYLA Oryzias latipes 8090
ORENI Oreochromis niloticus 8128
GASAC Gasterosteus aculeatus 69293
TETNG Tetraodon nigroviridis 99883
TAKRU Takifugu rubripes 31033
LATCH Latimeria chalumnae 7897
XENTR Xenopus tropicalis 8364
ANOCA Anolis carolinensis 28377
TAEGU Taeniopygia guttata 59729
MELGA Meleagris gallopavo 9103
CHICK Gallus gallus 9031
ORNAN Ornithorhynchus anatinus 9258
SARHA Sarcophilus harrisii 9305
MONDO Monodelphis domestica 13616
LOXAF Loxodonta africana 9785
MYOLU Myotis lucifugus 59463
AILME Ailuropoda melanoleuca 9646
CANFA Canis familiaris 9615
HORSE Equus caballus 9796
PIG Sus scrofa 9823
BOVIN Bos taurus 9913
RABIT Oryctolagus cuniculus 9986
SPETR Spermophilus tridecemlineatus 43179
CAVPO Cavia porcellus 10141
RAT Rattus norvegicus 10116
MOUSE Mus musculus 10090
OTOGA Otolemur garnettii 30611
CALJA Callithrix jacchus 9483
MACMU Macaca mulatta 9544
NOMLE Nomascus leucogenys 61853
PONAB Pongo abelii 9601
GORGO Gorilla gorilla 9595
PANTR Pan troglodytes 9598
HUMAN Homo sapiens 9606

Figure S6.

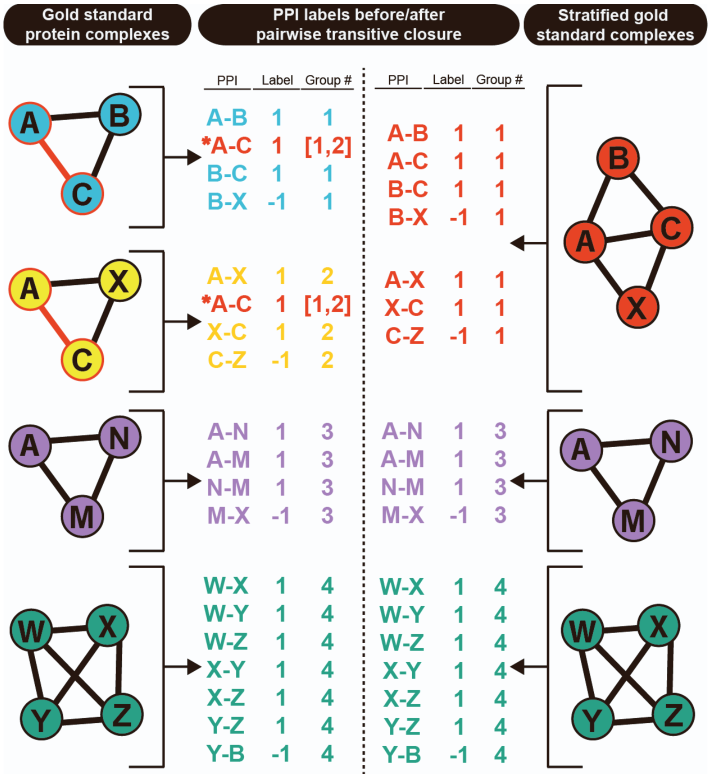

Gold standard protein complexes
PPI labels before/after pairwise transitive closure
Stratified gold standard complexes
PPI Label Group #
A-B 1 1
*A-C 1 [1,2]
B-C 1 1
B-X -1 1
A-X 1 2
*A-C 1 [1,2]
X-C 1 2
C-Z -1 2
A-N 1 3
A-M 1 3
N-M 1 3
M-X -1 3
W-X 1 4
W-Y 1 4
W-Z 1 4
X-Y 1 4
X-Z 1 4
Y-Z 1 4
Y-B -1 4
PPI Label Group #
A-B 1 1
A-C 1 1
B-C 1 1
B-X -1 1
A-X 1 1
X-C 1 1
C-Z -1 1
A-N 1 3
A-M 1 3
N-M 1 3
M-X -1 3
W-X 1 4
W-Y 1 4
W-Z 1 4
X-Y 1 4
X-Z 1 4
Y-Z 1 4
Y-B -1 4

Figure S7.

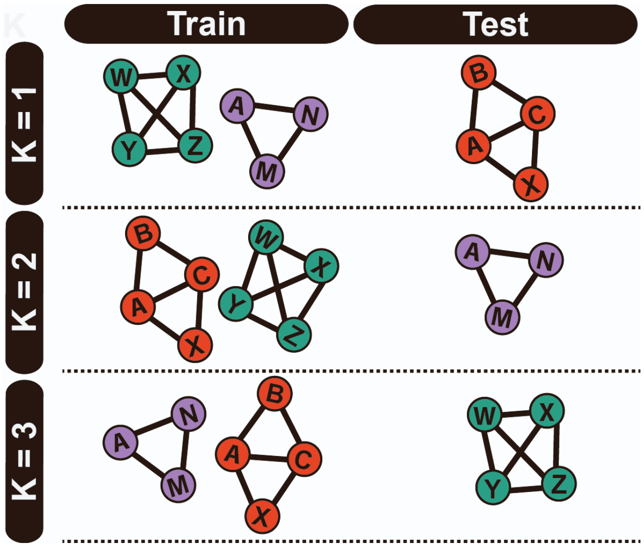

Figure S8.

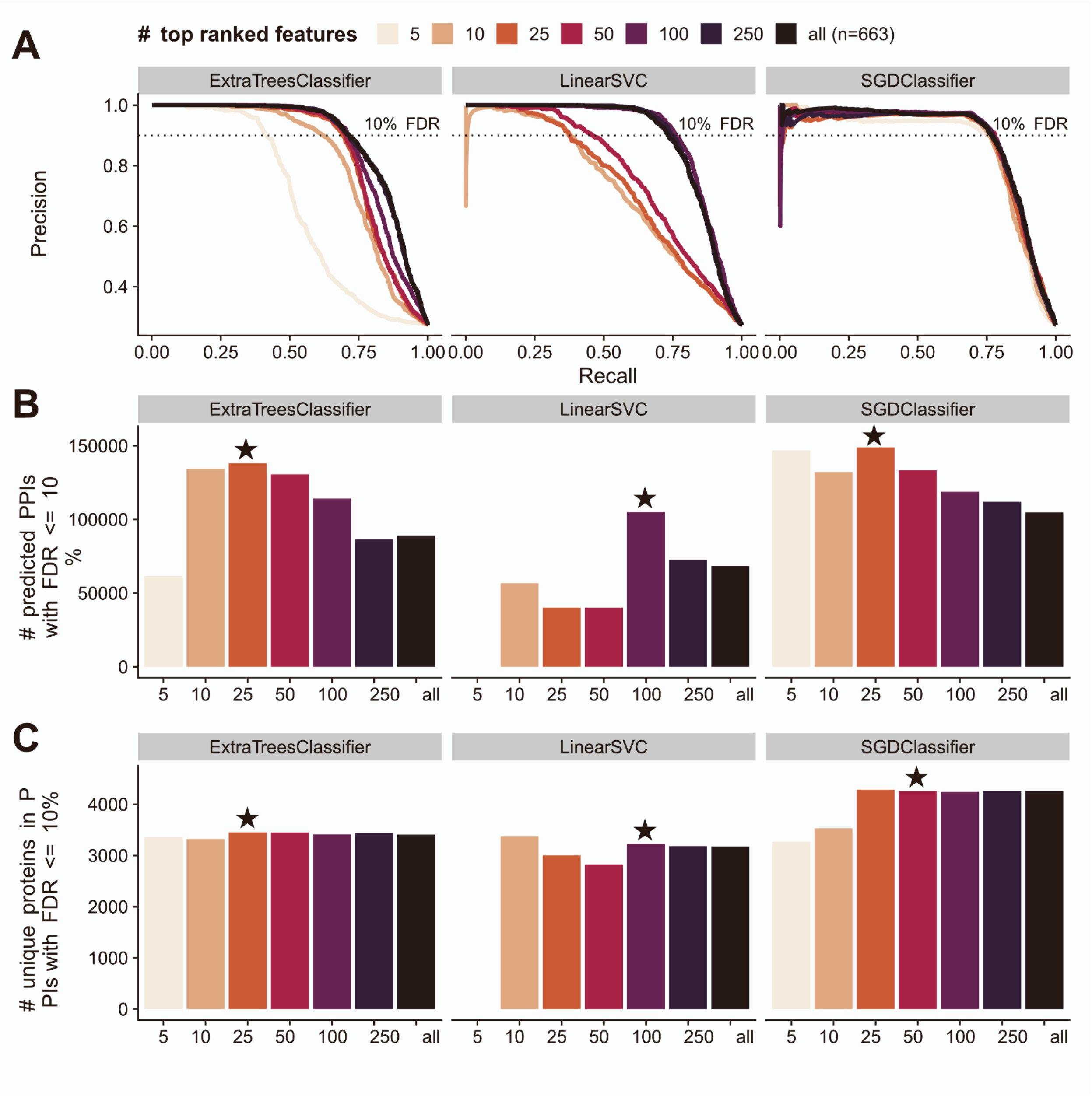

A
top ranked features
5
10
25
50
100
250
all (n=663)
ExtraTreesClassifier
LinearSVC
SGDClassifier
Precision
1.0
0.8
0.6
0.4
10% FDR
0.00
0.25
0.50
0.75
1.00
Recall
B
predicted PPIs with FDR <= 10 %
150000
100000
50000
0
5
10
25
50
100
250
all
C
unique proteins in PPIs with FDR <= 10%
4000
3000
2000
1000
0
