# Supplementary material for: A protein interactome for the last eukaryotic common ancestor illuminates the biochemical basis of modern genetic diseases
Source: Cell Genom. 2026 May 27;6(6):101254. doi: 10.1016/j.xgen.2026.101254 (PMC13261697; doi:10.1016/j.xgen.2026.101254)
Supplement: Document S2. Article plus supplemental information [file mmc6.pdf]

Cell Genomics

Article

A protein interactome for the last eukaryotic common ancestor illuminates the biochemical basis of modern genetic diseases

Graphical abstract

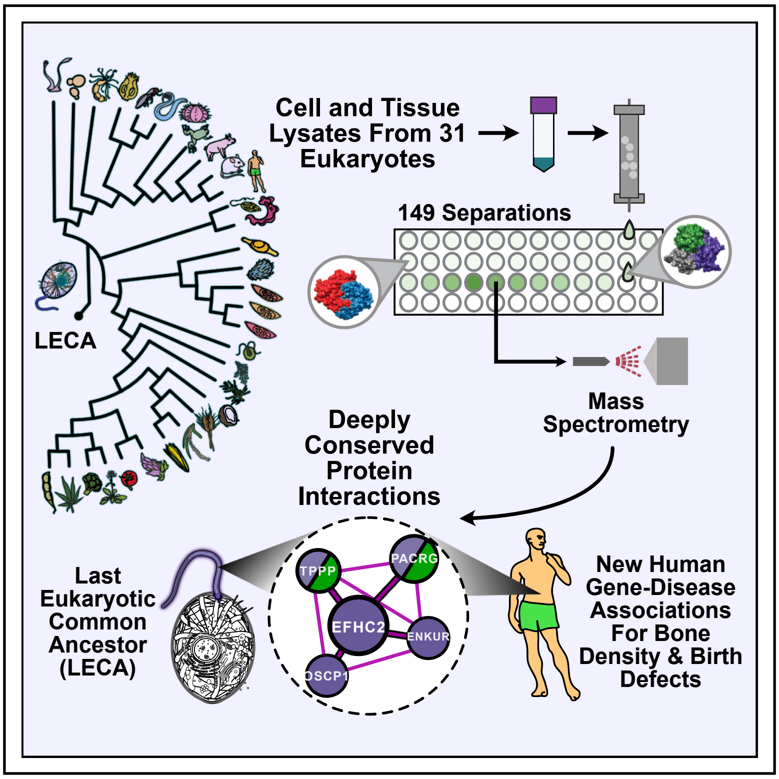

Highlights

- Defines the core biochemical organization of eukaryotes for nearly two billion years
- Deep conservation and loss of vesicle tethering complexes
- Ciliary mechanism in human EFHC2-associated renal failure
- Implicates V-ATPase subunit ATP6V1A in the etiology of mammalian osteopetrosis

Authors

Rachael M. Cox, Ophelia Papoulas, Shirlee Shril, ..., Friedhelm Hildebrandt, John B. Wallingford, Edward M. Marcotte

Correspondence

marcotte@utexas.edu

In brief

Cox et al. reconstruct the ancient network of protein interactions from the last eukaryotic common ancestor. These deeply conserved protein complexes are often critical to modern cells and suggest new links between genes and human disease, identifying new associations for bone density and congenital birth defects validated in animal models.

Cox et al., 2026, Cell Genomics *6*, 101254
June 10, 2026 © 2026 The Author(s). Published by Elsevier Inc.
https://doi.org/10.1016/j.xgen.2026.101254

CellPress

Cell Genomics

CellPress
OPEN ACCESS

Article

A protein interactome for the last eukaryotic common ancestor illuminates the biochemical basis of modern genetic diseases

Rachael M. Cox,[1] Ophelia Papoulas,[1] Shirlee Shril,[2] Chanjae Lee,[1] Tynan P. Gardner,[1] Zoya T. Ansari,[1] Anna M. Battenhouse,[1] Muyoung Lee,[1] Kevin Drew,[3] Claire D. McWhite,[4] David Yang,[1] Janelle C. Leggere,[1] Dannie Durand,[5] Friedhelm Hildebrandt,[2] John B. Wallingford,[1] and Edward M. Marcotte[1,6,*]
[1]Department of Molecular Biosciences, The University of Texas at Austin, Austin, TX 78712, USA
[2]Division of Nephrology, Department of Pediatrics, Boston Children's Hospital, Harvard Medical School, Boston, MA 02215, USA
[3]Department of Biological Sciences, University of Illinois at Chicago, Chicago, IL 60607, USA
[4]Department of Molecular and Cellular Biology, University of Arizona, Tucson, AZ 85721, USA
[5]Department of Biological Sciences, Carnegie Mellon University, 4400 5th Avenue, Pittsburgh, PA 15213, USA
[6]Lead contact
*Correspondence: marcotte@utexas.edu
https://doi.org/10.1016/j.xgen.2026.101254

SUMMARY

All eukaryotes share a single-celled ancestor from ~1.5 to 1.8 billion years ago, the so-called last eukaryotic common ancestor (LECA). Roughly half of gene families found in modern eukaryotes were already present in LECA, forming molecular systems that continue to influence genetic diseases and traits today. To investigate these systems, we compared genes across 156 organisms to define a core set of protein-coding gene families likely present in LECA, with a quarter remaining uncharacterized. Integrating >26,000 mass spectrometry proteomics analyses from 31 species, we inferred higher-order complexes among these ancient proteins. This reconstructed interactome reveals both established and novel assemblies, offering a biochemical snapshot of LECA's organization. Finally, by exploring these ancient protein interactions, we found new human gene-disease associations for bone density and congenital birth defects, illustrating the value of ancestral protein networks for modern functional genetics.

INTRODUCTION

The last eukaryotic common ancestor (LECA), existing ~1.5–1.8 billion years ago, was the unicellular ancestor of all extant eukaryotes.[1,2] Its molecular makeup offers insights into the ancient innovations underlying modern eukaryotic cellular complexity. As such, understanding the core genetic toolkit of LECA has been a long-standing goal in genetics and evolutionary biology.

Previous phylogenomic analyses agree that LECA was highly complex and contained most hallmarks of the eukaryotic cell.[3] Synthesis of these studies suggests LECA had at least one nucleus[4,5] with linear chromosomes and centromeres[6]; an interconnected endomembrane system comprised of an endoplasmic reticulum,[7] Golgi apparatus,[8] vesicle trafficking system,[9] and nuclear envelope[10]; a dynamic actin- and tubulin-based cytoskeleton[11] including pseudopodia,[12] centrioles,[13] and at least one cilium[14]; distinct degradative vesicles such as lysosomes[15] and peroxisomes[16]; and mitochondria capable of both aerobic and anaerobic respiration.[2,17]

While there are many partial descriptions of the genetic content of LECA, including systematic reconstructions of many of LECA's genes,[18–22] there is as yet no integrated picture of LECA's proteome or how these proteins interact in higher-order assemblies. Such interactome data are valuable because most cellular processes rely on protein assemblies.[23] Thus, an interactome for LECA would provide a richer portrait than genomic reconstructions alone. Moreover, since proteins are the primary drivers of molecular phenotype,[24] protein interaction networks are valuable tools for inferring protein functions and uncovering genotype-to-phenotype relationships of medical and agricultural interest.

Previous efforts to map eukaryotic protein interactomes systematically have primarily concentrated on opisthokonts (i.e., animals and fungi)[25–31] with the exception of a handful of studies in plants[32–34] and protists.[35,36] However, comparing protein networks across more divergent species could provide insights not only into evolutionary conservation, but also into the divergence of specific biological processes. For example, conserved machinery can be used in different organism-specific contexts to produce distinct organism-specific phenotypes (e.g., the "phenolog hypothesis"[37]).

Here, we reconstruct the LECA protein interaction network and use it to illuminate human genetic disease. We first derived a preliminary estimate of the LECA protein-coding gene set as a basis for interpreting proteomics data. We then integrated ~26,000 mass spectrometry experiments across 31 eukaryotes spanning ~1.8 billion years of evolution, including unicellular and multicellular taxa, parasites and free-living species, and motile

© 2026 The Author(s). Published by Elsevier Inc.
This is an open access article under the CC BY license (http://creativecommons.org/licenses/by/4.0/).

and non-motile organisms, representing a diverse sampling of eukaryotic cell types, morphologies, lifestyles, and metabolic capacities. The resulting draft map comprises protein interactions present in LECA and widely conserved across the eukaryotic tree of life. Finally, we leveraged this ancient network to predict human gene-disease phenotypes, validated in frogs and mice. Thus, the LECA interactome provides both a framework for understanding eukaryotic origins and a tool for identifying disease-associated proteins.

RESULTS AND DISCUSSION

Inferring the gene content of LECA, the last eukaryotic common ancestor

We inferred LECA's likely gene content using multiple complementary ancestral reconstruction approaches applied to gene families defined by eggNOG orthogroups.[38] As a computationally tractable baseline, we used Dollo parsimony[39] to model gene duplication and loss across 156 reference proteomes (see STAR Methods and supporting Zenodo repository), yielding 10,091 candidate ancestral gene families, hereafter referred to as LECA orthogroups (OGs) (Table S1). To assess robustness—particularly regarding horizontal gene transfer—we evaluated each orthogroup using two additional phylogenetic frameworks that relax Dollo's single-origin assumption: Wagner parsimony and gene tree-species tree reconciliation. A substantial fraction of Dollo-inferred OGs (6,429; 64%) were independently supported by these approaches (Figure S1). We therefore distinguish a "core" LECA OG set supported by multiple methods and restrict all gene- and pathway-specific biological interpretation to this subset. This consensus approach leverages Dollo's sensitivity (thereby missing fewer families) while mitigating its biases.

While this LECA gene set is a model-dependent approximation for which caveats exist (see STAR Methods for discussion of limitations), its size (6–10K gene families) is consistent both with previous literature estimates[3,19,21] and with the notion that early eukaryotes were, at minimum, a fusion of at least two prokaryotes' worth of genes—an archaeal host and a eubacterial mitochondrial endosymbiont. Within the LECA OGs, we recover genes consistent with eukaryotic features inferred from ancestral trait reconstructions, seen by mapping OGs to known functional annotations.[38] Figure 1 highlights LECA OG functions associated with the nucleus, endoplasmic reticulum, Golgi apparatus, endosomes, digestive vesicles, transport vesicles, secretory vesicles, mitochondria, cilium, and an extensive cytoskeleton likely capable of cell projection.

Approximately 25% of functionally annotated LECA OGs relate to DNA replication/repair, transcription, translation, and RNA processing, underscoring eukaryote-specific innovations related to the segregation of transcription and translation within the nucleus and cytoplasm, respectively.[41] Examples of these innovations are the spliceosome, nuclear envelope, nuclear pore complex, and transport factors such as karyopherins regulated by the Ran-GTP system.[10,42] Another ~30% relate to the compartmentalization of energy production, energy conversion, and metabolism.[43] For example, we recover all V-ATPase subunits, a conserved protein complex responsible for the acidification of lysosomes and peroxisomes. Additionally, we traced mitochondrial-specific genes such as TIM and TOM translocases to LECA, in addition to a large suite of mitochondrial carrier family (MLF) proteins and generalist solute carrier (SLC) family proteins. Continuous MLF- and SLC-mediated transport of myriad metabolites across the mitochondrial membrane enables multiple modes of energy conversion,[44] suggesting that LECA had already evolved discriminatory pathways necessary for precise partitioning of varied substrates.

LECA also possessed a rich system of endomembranes, with ~15% of LECA OGs associated with membrane biogenesis, trafficking, and signaling. These include endosomal coat proteins such as clathrin, adaptin, and the COPI and COPII vesicle coat complexes which facilitate vesicle budding from the Golgi apparatus and endoplasmic reticulum, respectively.[9] Proteins related to signal transduction underwent a massive expansion at the root of eukaryotes, particularly within GTPases, such as the Ras, Ran, Rho, Rab, Arf, and dynamin superfamilies,[45] highlighting the complexity of early signaling pathways and their links to endomembranes. Surprisingly, using eggNOG functional annotations we only found ~300 LECA OGs (~3%) associated with the cytoskeleton and cell motility (i.e., a cilium) while previous studies reported ~500 OGs.[46,47]

Finally, the eggNOG algorithm identified 2,387 LECA OGs (~25%) of unknown function (Figure 1, bottom right), many of which are absent from the human genome. The high proportion of uncharacterized genes and low count of ciliary genes prompted us to attempt to assign function, or at a minimum, subcellular location, to LECA OGs by using annotations for extant proteins in UniProt[48] (see STAR Methods and Zenodo repository). This allowed us to recover ~200 LECA OGs associated with cytoskeletal and cell motility pathways that were previously classified as "function unknown" by the eggNOG algorithm, bringing the total up to the ~500 expected OGs based on previous literature.[46,47] In sum, we assign tentative UniProt annotations and subcellular compartments to 1,066 of 2,387 LECA OGs originally categorized as "function unknown" by eggNOG.

Overall, slightly more than half of human genes (n = 13,571) map to 4,777 unique LECA OGs (of which 93% are core LECA OGs), comparable in scale to previous estimates,[49] and the differences in conservation can be instructive. For example, the human genome appears to have lost many genes associated with carbohydrate metabolism, amino acid metabolism, and membrane biogenesis, including those for isocitrate lyase (ICL) and malate synthase, enzymes of the glyoxylate cycle. Glyoxylate is a highly reactive aldehyde and glyoxylate cycles have been demonstrated in nearly every other branch of life outside of mammals.[50,51] Because of malate synthase loss, human cells must rely on other enzymes to neutralize glyoxylate: alanine-glyoxylate aminotransferase (AGT) in the peroxisome or glyoxylate reductase (GRHPR) in the cytosol. Defects in either human enzyme produce primary hyperoxaluria, i.e., renal disease caused by the failure to detoxify glyoxylate.[52] This example highlights how comparative evolution informs mechanisms of human disease.

Mapping the LECA protein interactome

We next sought to reconstruct LECA protein organization using co-fractionation mass spectrometry (CFMS), a high-throughput

CellPress
OPEN ACCESS

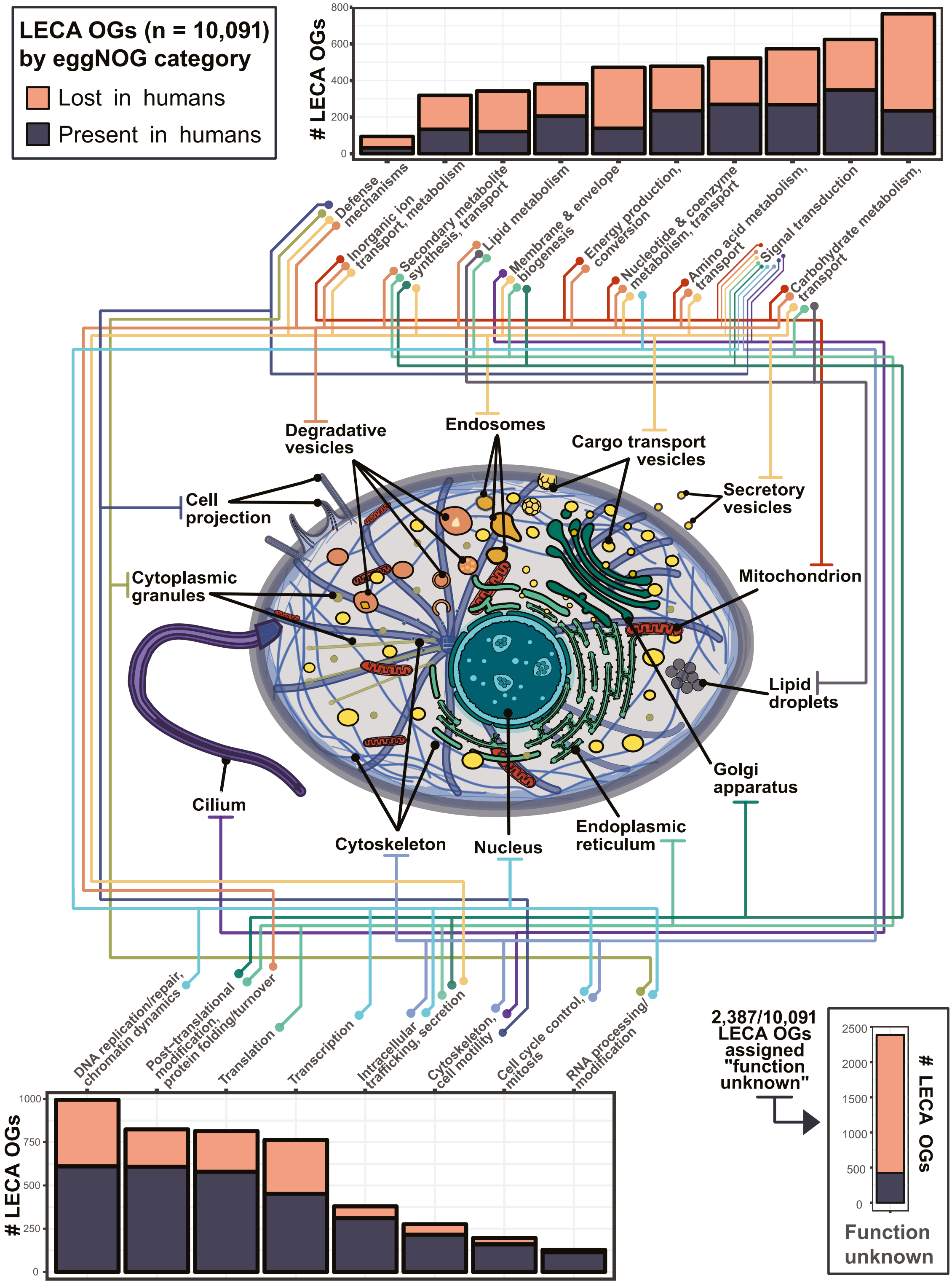

Figure 1. Inferred subcellular organization in LECA, the last eukaryotic common ancestor, based on its estimated gene content

Cell illustration adapted from multiple graphics sourced from SwissBioPics.[40]

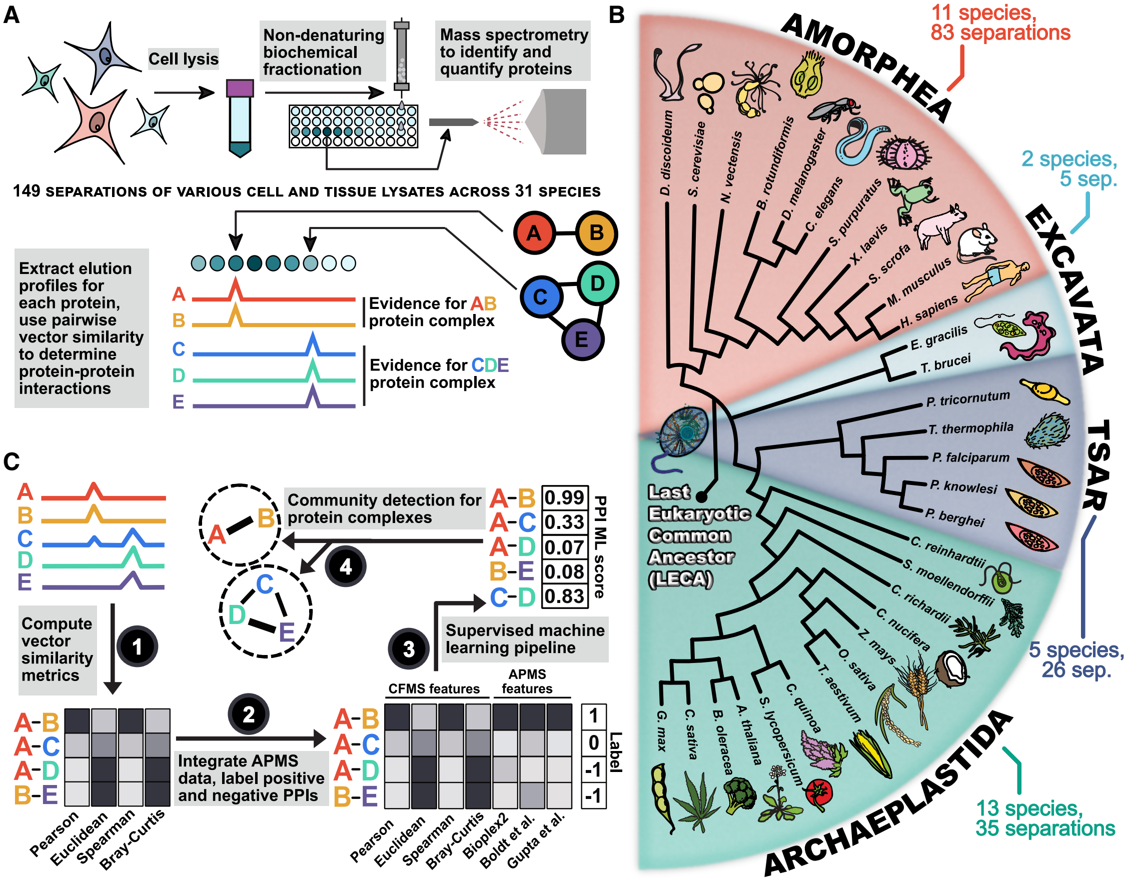

Figure 2. Overview of experimental and computational methods

(A) Schematic representation of a co-fractionation mass spectrometry experiment.

(B) Proteomics data used to construct the LECA interactome included eukaryotes spanning ∼1.8 billion years of evolution. Tree structure is based on Koumandou et al.[22] Branch lengths are not drawn to scale.

(C) Schematic overview of the approach for computing protein-protein interaction (PPI) features based on CFMS (1) and APMS (2) datasets, scoring conserved PPIs based on these features (3), and clustering scored PPIs into complexes (4).

method for measuring protein-protein interactions (PPIs) without tags or affinity reagents (Figure 2).[28,53] CFMS reduces the complexity of a native protein lysate by gently separating protein complexes based on properties such as size, charge, or hydrophobicity, as stably interacting proteins generally co-elute irrespective of the (native) separation. While a single CFMS experiment is insufficient to reliably infer specific protein-protein interactions, integrating observations from orthogonal separations from multiple species, cell types, and tissues confers strong statistical power for inferring conserved interactions.[29,31,33]

To detect conserved protein interactions, we integrated raw CFMS data from more than 10,000 individual biochemical fractions[28,29,33,35,36] across 31 diverse eukaryotic species spanning ∼1.8 billion years of evolution (Figure 2B). We also generated new CFMS data for four minimally characterized, phylogenetically diverse unicellular eukaryotes: *Brachionus rotundiformis* (Amorphea, rotifer), *Euglena gracilis* (Excavata, algae), *Phaeodactylum tricornutum* (TSAR, diatom), and *Tetrahymena thermophila* (TSAR, ciliate). Because LECA was ciliated, we expanded our coverage of ciliary proteomes by collecting CFMS data from *Sus scrofa* (Amorphea, pig) tracheal tissue and *Xenopus laevis* (Amorphea, frog) sperm. Experimental details for each separation are provided either in the STAR Methods section or in the PRIDE database (accessions in the Key Resources Table).

In all, we measured 379,758,411 peptides that were uniquely assigned to 259,732 orthologous groups (or unique proteins not mapping to orthogroups) across the 31 species, 149 separations, and 10,481 fractions (Figure 3A). We augmented our CFMS data with ∼15,000 proteomics experiments[30] that included affinity purification mass spectrometry (APMS),[54–56] proximity labeling,[57,58] and RNA-pulldown data.[59] In total, we incorporated data from 26,297 mass spectrometry experiments. We then filtered these data such that we retained only LECA OGs that were strongly observed, in that the sum of peptide spectral matches (PSMs) across all 149 fractionations was greater than or equal to 150 PSMs. This yielded elution profiles for 5,989 well-measured LECA OGs (∼60% of all LECA OGs and 88% of core OGs).

CellPress
OPEN ACCESS

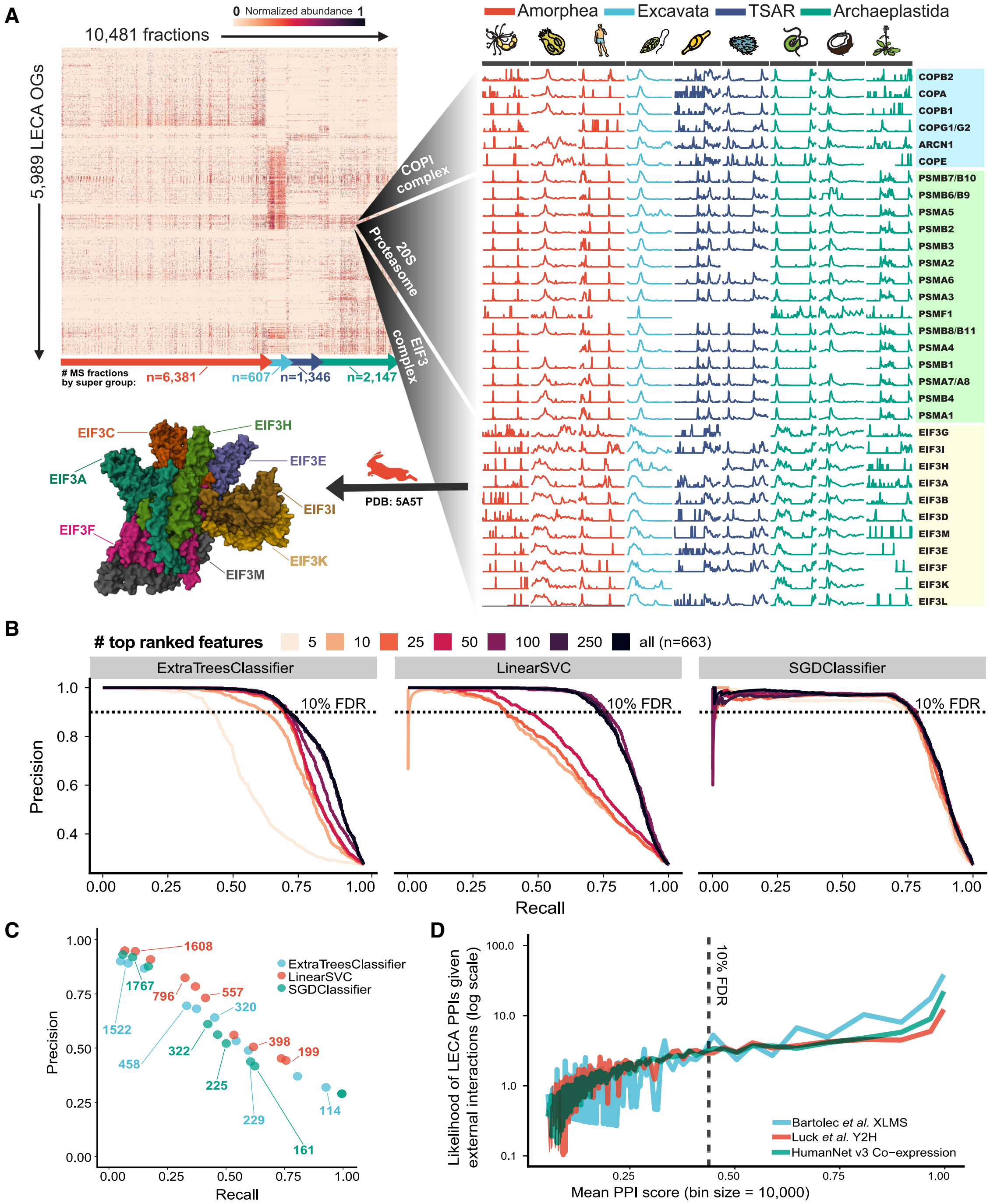

Figure 3. Determining the LECA protein interactome

Co-elution matrix and results of the protein interaction machine learning pipeline.

(A) Heatmap of the filtered elution matrix for 5,989 strongly observed LECA OGs across 10,481 CFMS mass spectrometry fractions (left) and an expanded view of elution vectors for the COPI, 20S proteasome, and eukaryotic initiation factor 3 complexes for a subset of species (right).

(B) Precision-recall performance of three classifiers trained with increasingly larger sets of ranked features.

(C) Precision-recall curves for reconstructing known protein complexes defined by a walktrap algorithm, where pairwise PPI scores from each classifier are used as input. Points are labeled with the total number of protein clusters (complexes) constructed at each point in the hierarchy.

(D) The likelihood of LECA PPIs being present in external protein-protein or mRNA coexpression networks as a function of LECA PPI score, showing agreement increases with scores.

While many complex subunits have co-elution profiles with visually detectable correlation (as for the members of the COPI vesicle coat complex, 20S proteasome, and eukaryotic initiation factor 3 in Figure 3A, right), a computational framework is required for systematic identification and to properly control for false positives (Figure 2C). To this end, we employed a supervised machine learning pipeline trained on our data for known protein complexes. We assembled a set of 1,499 known complexes from two databases that record PPIs for a variety of eukaryotic species[60,61] (see STAR Methods). We then assessed a number of interaction prediction models generated by three different classification algorithms: extremely randomized trees (ExtraTreesClassifier), linear support vector (LinearSVC), and stochastic gradient descent (SGDClassifier).

We constructed models with variable numbers of features derived from the datasets, ranking features on a classifier-by-classifier basis, and evaluated performance by measuring the precision and recall of known complexes withheld from the training data (Figure 3B).

Initial models achieved 5%–38% recall at 90% precision, similar to previous large-scale protein interaction maps.[30,31,33] However, after implementation of a custom data stratification approach (see STAR Methods), performance improved to 40%–90% recall at 90% precision. We suspect that this large jump in performance stemmed from a combination of the large volume of high-quality mass spectrometry data being integrated and the data stratification approach, which we found significantly reduced overfitting (see STAR Methods for details and also for additional discussion of challenges and limitations).

As the CFMS datasets intrinsically capture higher-order multiprotein assemblies (as in Figure 3A), we sought to define these assemblies by clustering the LECA OGs based on the measured pairwise interactions. Using each classifier and its best performing feature set, we identified PPIs with a 10% false discovery rate (FDR) threshold (see Zenodo repository) and hierarchically clustered the proteins into complexes on the basis of these interactions (weighted by their confidence scores) using an unsupervised community detection "walktrap" algorithm,[62] which has previously been shown to perform well on CFMS datasets.[33] Rather than rely on a single clustering threshold for determining protein complexes, we defined a hierarchy of protein interactions (Table S2), reflecting the natural tendency of proteins to associate into sub-complexes, which assemble into larger complexes, and bind accessory factors. The walktrap procedure also determined an "optimal" number of subcommunities to range between 100 and 400, differing significantly by classifier. These large clusters capture the general organization of eukaryotic cells (e.g., partitions include a large spliceosomal cluster, a large chromosomal maintenance cluster, clusters broadly associated with cilia, etc.).

As a positive control, we noted that this approach successfully delineated known complexes. For example, a large spliceosome cluster was demarcated into LSM, PrP19 complex, and U4/U6 x U5 tri-snRNP complexes with increased granularity (Table S2). To quantify walktrap performance, we calculated precision and recall for each cluster at each level of the hierarchy for each classifier (Figure 3C), observing a tradeoff where finer subcommunities have increased precision but decreased recall for capturing known complexes. Overall, the support vector classifier (Figure 3C, red) netted the highest quality protein complexes and was chosen as our final model. The full hierarchy of LECA protein associations into complexes is reported in Table S2.

We assessed how well our final protein interaction model agreed with independent studies that defined interactions using orthogonal approaches. We observe that protein pairs within our highest scoring threshold ($\leq$10% FDR) are significantly more likely than random chance to agree with yeast 2-hybrid (Y2H),[63] mRNA co-expression,[64] and cross-linking mass spectrometry (XLMS)[65] interactions (Figure 3D) and performed comparably with a previous interaction map in plants.[33] The resulting final LECA complexome consists of the highest confidence 109,466 pairwise interactions between 3,193 unique OGs (of which 98% are core LECA OGs), hierarchically assembled into 199 (less granular) to 2,014 (more granular) protein complexes, which are portrayed schematically in Figure 4.

Importantly for our inference of conserved protein interactions, our method does not rely on any single ancestral reconstruction model. Each interaction in the final network requires independent proteomic evidence from multiple major eukaryotic lineages, such that the interaction map is driven by experimental co-fractionation data rather than by phylogenetic modeling alone. With this experimentally determined set of conserved LECA PPIs in hand, in the next sections, we next examined the extent to which our interactome both recapitulates previously hypothesized and discovers new LECA complexes. Current phylogenomic studies hypothesize myriad protein assemblies at the root of eukaryotes.[6,9,10,22,66–68] We focused on ancient protein assemblies related to intracellular trafficking and cell projection because these are highly relevant to modern human disease[69–71] and highlight below multiple examples where we uncovered previously undescribed interactions.

Deep conservation and loss of vesicle tethering complexes

One hallmark of eukaryotic cells is intracellular trafficking by cargo-laden vesicles that bud from one compartment and fuse to another, supported by coat proteins (e.g., clathrin, COPI, and COPII), membrane-anchored SNARE proteins that facilitate fusion,[72] and tethering factors that ensure target specificity.[73] Many compartment-specific tethering modules were likely present in LECA (as in Koumandou et al.[66]), including the ER-associated TRAPP-I, TRAPP-II, and TRAPP-III complexes, the Golgi-associated retrograde protein (GARP), the conserved oligomeric Golgi (COG) complexes, the endosome-associated recycling protein (EARP), and the endolysosomal homotypic fusion and protein sorting (HOPS) complexes. However, the extent to which particular protein interactions are conserved remains unknown. In our LECA interactome, we recover all these core tethering assemblies, along with some unexpected members (Figure 5A). These inferences are based on interactions observed across multiple supergroups and restricted to core OGs.

The GARP and EARP complexes are closely related and share three subunits (VPS51, VPS52, and VPS53).[74] Localization is conferred by additional subunits: VPS50 for endosomes and VPS54 for the Golgi.[74] We observe strongly conserved

CellPress
OPEN ACCESS

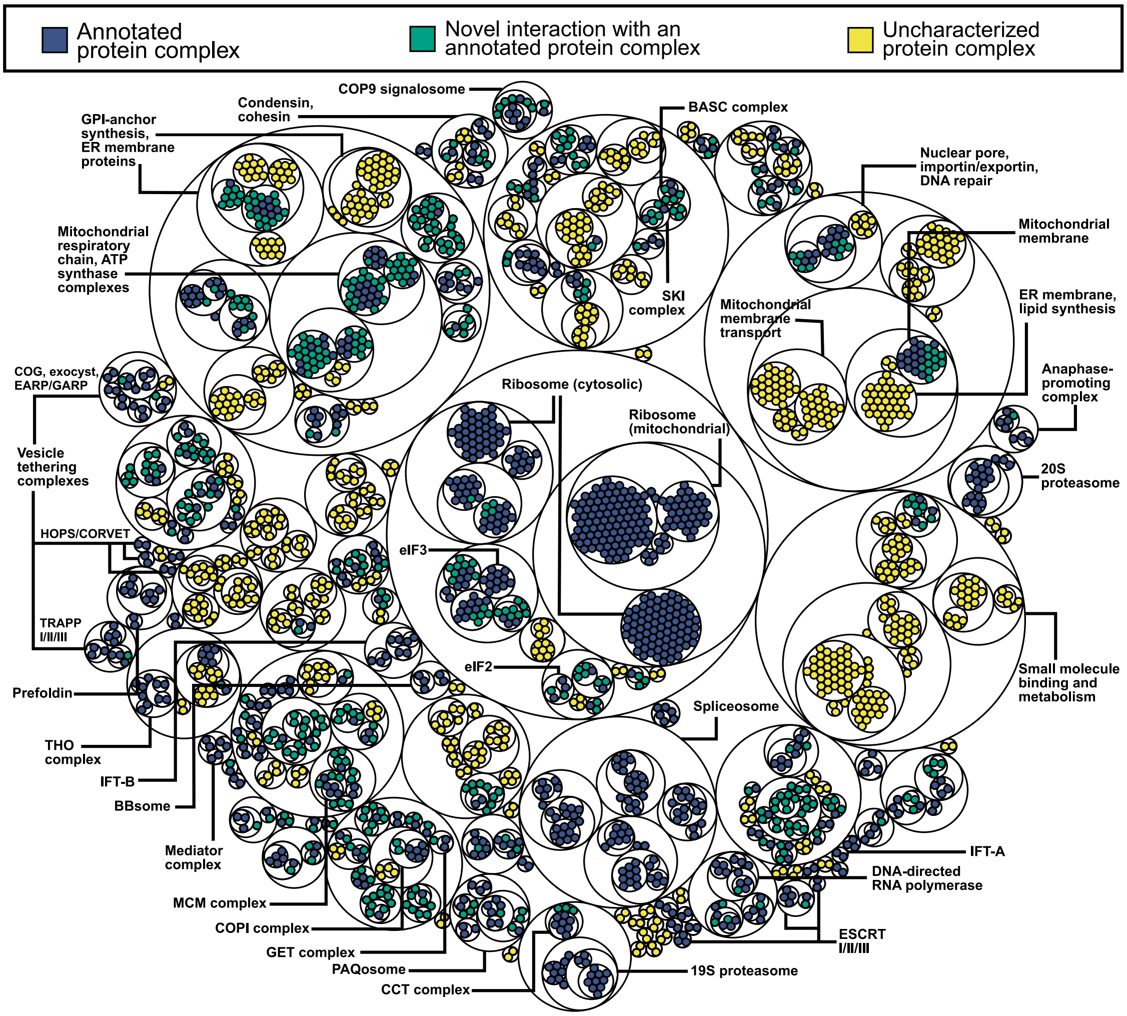

Figure 4. Visualizing hierarchical clustering of protein complexes for a subset of the conserved eukaryotic interactome

The smallest diameter circles correspond to individual proteins, colored by whether the proteins in a cluster are known to interact with each other (blue), whether a novel protein is interacting with a known complex (green), or whether all the associations within a cluster are uncharacterized (yellow).

interactions between all of these subunits, in addition to EIPR1 (EARP and GARP complex-interacting protein 1) (Figure 5A, top right). The interaction of EIPR1 with GARP/EARP was only recently discovered, first in high-throughput screens of human proteins[54,56] and then confirmed in human neuroglioma cells.[75] While EIPR1 is widely conserved, we find that its interaction with GARP/EARP is also ancient and likely traces back to LECA.

In modern eukaryotes, the HOPS complex shares four of its six subunits (VPS11, VPS16, VPS18, and VPS33) with the related CORVET complex, while the remaining two (VPS39 and VPS41) are unique to HOPS. In yeast, CORVET subunits direct the fusion of early and recycling endosomes while HOPS directs the fusion of late endosomes, lysosomes, and autophagosomes.[76] We observe conserved interactions between VPS8 (previously thought to be CORVET specific) and the VPS16, VPS18, VPS39, and VPS41 subunits of the HOPS complex (Figure 5A, top left), raising the possibility that a single HOPS-like complex in LECA may have governed endolysosomal vesicle fusion, with subsequent lineage-specific duplication and specialization of subunits for different compartments.

Analogously, the ER/Golgi-associated TRAPP complex is thought to be composed of five core subunits (TRAPPC1-5) with additional subunits in distinct TRAPP-I (TRAPPC6), TRAPP-II (TRAPPC9, TRAPPC10, and TRAPPC13), and TRAPP-III (TRAPPC8) complexes. However, the number and identity of proteins in TRAPP-I/II/III vary by species.[77] In our ancient interactome, we observe strong interactions between each member of the core C1-C5 complex, conserved across all sampled eukaryotic supergroups. Unexpectedly, we find pan-eukaryotic evidence for TRAPPC12 in this core complex, previously thought to be metazoan specific.[78,79] The remaining interactions are differentially lost in specific eukaryotic lineages, with TRAPPC10 absent in all five sampled TSAR species. Our data, combined with conflicting literature on the exact composition of TRAPP-I/II/III, thus suggest an ancient and flexible core

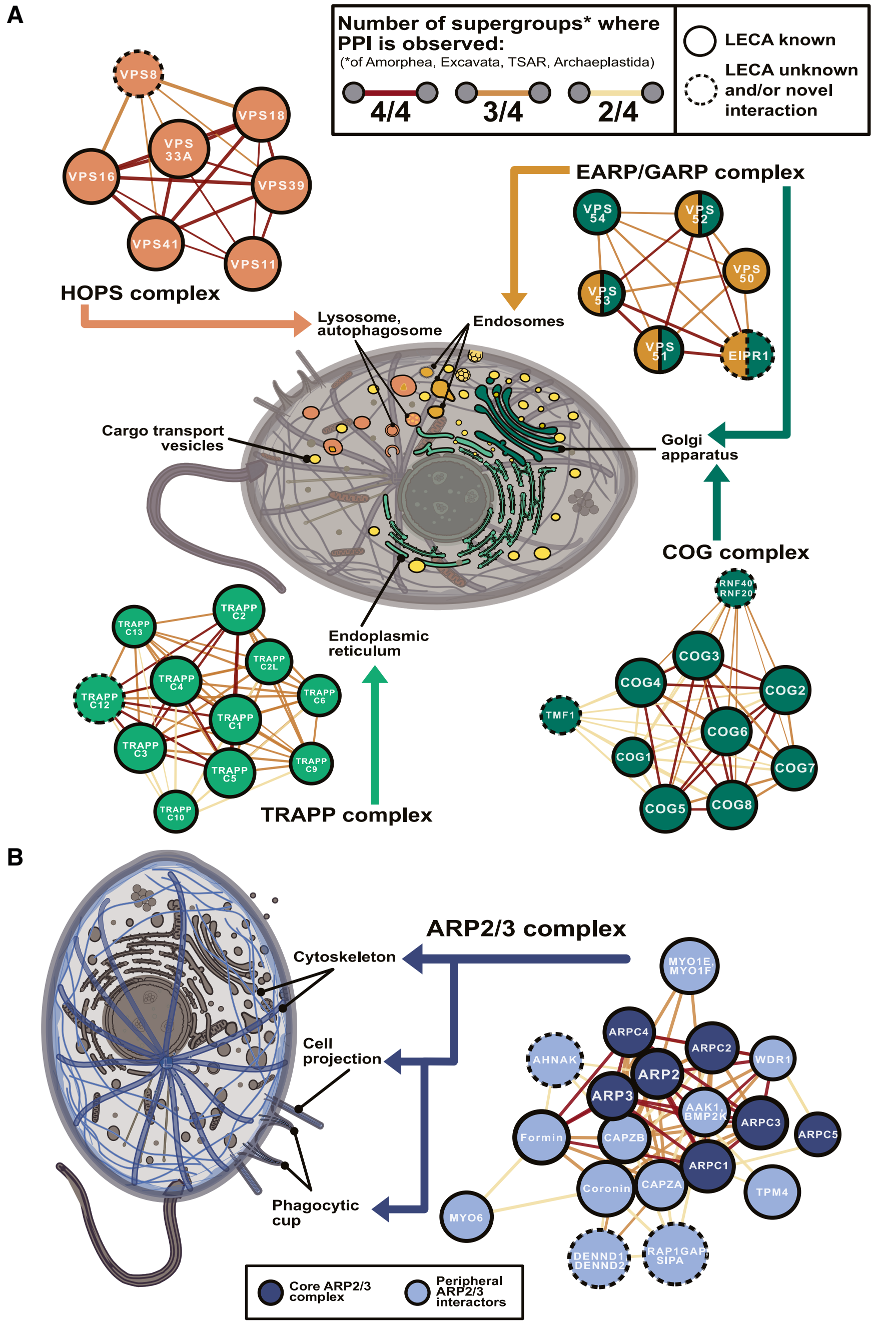

Figure 5. Notable LECA systems related to vesicle tethering and cell projection

(A) Node colors for each vesicle tethering complex correspond to their primary subcellular localization: endoplasmic reticulum (light green), Golgi apparatus (dark green), digestive vesicles (orange), or endosomes (yellow).

(B) Dark and light blue nodes depict core and peripheral cell projection components.

In both (A) and (B), edges between proteins are colored by the number of eukaryotic supergroups in which the interaction is observed, i.e., possesses elution vectors exhibiting a minimum Pearson r of 0.3 within species in two or more of the four eukaryotic supergroups Amorphea, Excavata, TSAR, and/or Archaeplastida (see Figure 2), with darker red edges indicating interactions observed in all supergroups considered.

complex where subunits differentially specialize along different eukaryotic branches.

The eight-subunit COG assembly governs retrograde intra-Golgi trafficking and comprises two heterotrimeric subcomplexes (COG2-4 and COG5-7) linked by a COG1-COG8 heterodimer.[80] Our LECA interactome recapitulates this assembly and includes interactions with TMF1 and the ubiquitin ligase complex RNF20-RNF40 (Figure 5A, bottom right). TMF1-COG interactions have been previously observed only for metazoan COG2 and COG6,[81] but our data show confident TMF1-COG interactions spanning Amorphea and Archaeplastida, with TMF1 lost in Excavata and TSAR. RNF20-RNF40 is generally described in nuclear roles like histone ubiquitination, transcription regulation, and DNA damage repair[82,83] but has been also linked to the Golgi-associated adapter protein WAC,[84] involved in Golgi membrane fusion.[85,86] We see conservation of RNF20-RNF40 interactions with COG across Amorphea, TSAR, and Archaeplastida, suggesting that nuclear repurposing of this ubiquitin ligase complex may be a recent mammalian innovation.

Thus, the LECA interactome reveals conservation and specialization of eukaryotic vesicle tethering complexes. We identified unexpected ancient interactions, such as those involving EIPR1 with GARP/EARP, TMF1 and RNF20-RNF40 with COG, and TRAPPC12 with the TRAPP complex, and additionally saw evidence for flexible and lineage-specific adaptations. Given this utility for examining evolutionary conservation and diversification of LECA-associated complexes, we next applied it to shed light on a central question of LECA evolution that is in dispute.

Primordial origins of cell projection and phagocytosis

While phagocytosis is widely observed across eukaryotes, it remains debated whether LECA could recognize and engulf large particles. Contention stems from how the first eukaryotic common ancestor (FECA) acquired the alpha-proteobacterial precursor of the mitochondrion, i.e., whether FECA was akin to a phagocytosing archaeon or a more “simple” prokaryote that existed in protracted syntrophy with an alpha-proteobacterium.[87–89]

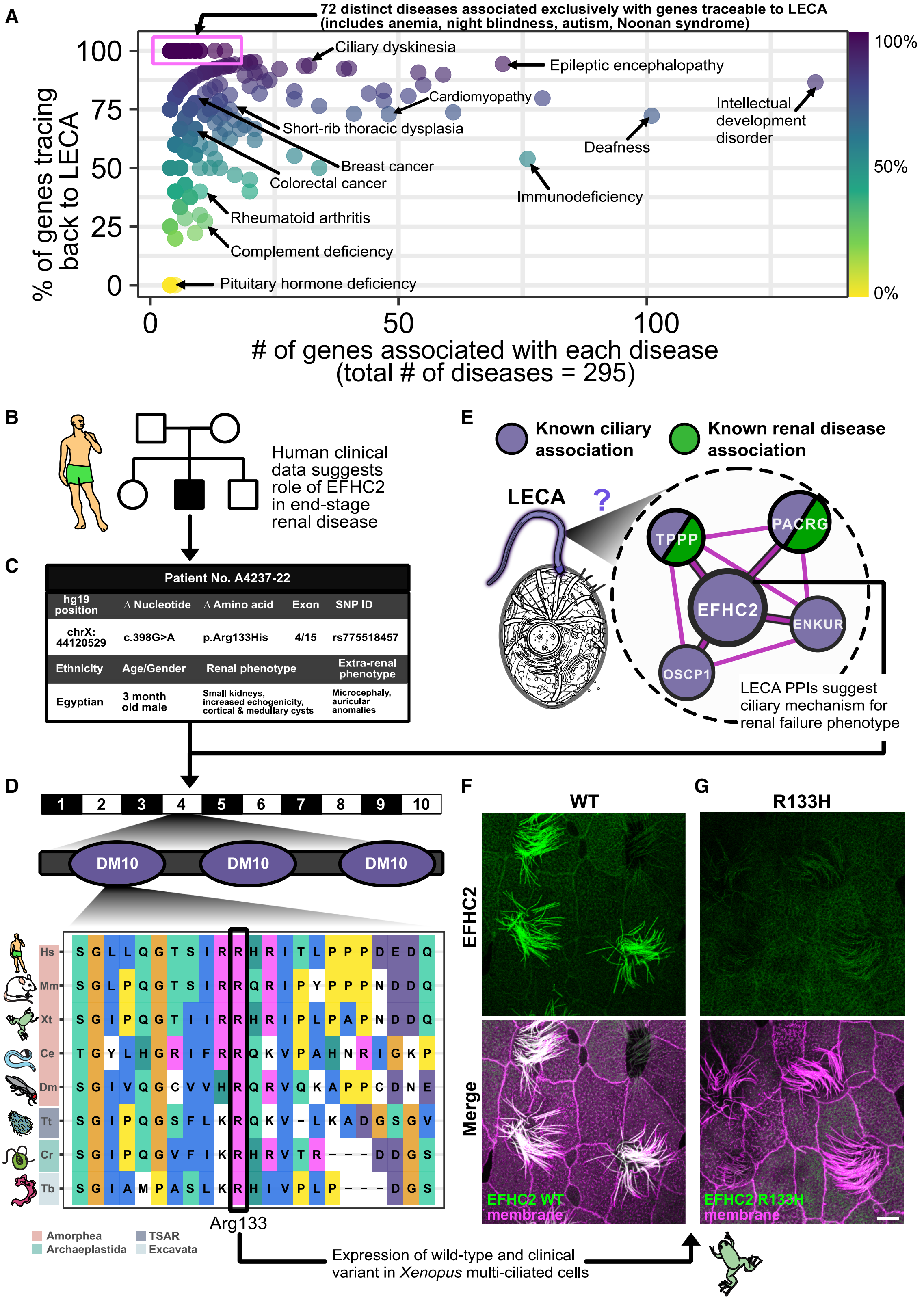

(legend on next page)

Existing phylogenomic investigations into the origins of phagocytosis are conflicting; at least three independent studies conclude phagocytosis probably evolved independently in multiple eukaryotic lineages,[90–92] while others argue that the trait was present in LECA and the absence of phagocytosis in certain eukaryotic groups is due to secondary loss when adapting to new niches.[93,94] Recent investigations into Asgard archaea, the sister group to eukaryotes, reveal a dynamic actin-based cytoskeleton composed of F-actin assemblies, actin-related proteins (Arps), and actin-binding proteins such as profilins and gelsolins capable of modulating eukaryotic actin,[95–97] suggesting that FECA may have had phagocytic capacity.

Within our LECA gene set, we find an extensive complement of OGs associated with both cell projections (pseudopodia, lamellipodia, filopodia) and phagocytosis (phagocytic cups and phagosomes) (Figures 1 and 5B). Specifically, LECA appears to have had Rho GTPases such as RAC/CDC42, formins, coronins, cofilins, gelsolins, and proteins associated with the ARP2/3, ENA/VASP, WASP, SCAR/WAVE, and PI3K complexes. For example, the LECA interactome includes all seven subunits of the ARP2/3 complex, responsible for the cytoskeletal rearrangement required for cell projection, clustering with protein complexes involved in cell protrusion and proteins critical for phagocytosis in extant eukaryotic cells (Figure 5B). The core ARP2/3 complex consists of the proteins ARP2, ARP3, ARPC1, ARPC2, ARPC3, ARPC4, and ARPC5. Interestingly, in our data, ARPC5 is the most peripherally associated component in the cluster and has been completely lost in all species sampled within Excavata and TSAR.

Combined with previous evidence,[98–100] these genes suggest that the ancestral eukaryotic cell was likely capable of pseudopod formation and projection-based motility, despite the lack of UniProt annotations in species outside Amorphea (Figure S2). However, because cell projections and phagocytosis share underlying molecular machinery, it is less clear if the presence of these systems necessarily imply a phagocytosing LECA, and more evidence is required to conclude that phagocytosis is ancestral at the root of eukaryotes. To address this, we explored the conservation of additional interactions.

Among peripheral interactors of the ARP2/3 complex, we observe CAPZA and CAPZB forming the heterodimeric F-actin capping complex, an essential regulator of actin nucleation that restricts elongation,[101] as well as formins and coronins known to promote elongation.[98,102] We also find interactions with WDR1, a promoter of cofilin-mediated actin severing[103] that assists both actin polymerization and depolymerization.[104] Research strongly implicates these systems in Amorphean phagocytosis: coronins are strongly enriched in phagocytic cups and defects result in impaired phagocytosis in both *Dictyostelium* and mammalian cells.[105–107] Furthermore, we observe interaction evidence in at least two major eukaryotic supergroups consistent with the reported roles of AAK1 in receptor-mediated endocytosis[108] and unconventional myosin in phagocytosis.[109–112] Taken together, our results strongly support a phagocytosing LECA.

The LECA interactome reveals a ciliary mechanism for *EFHC2*-associated renal failure

Conservation of protein interactions over billions of years implies strong constraint and likely pathogenicity when disrupted. Thus, studying genetic variation through the lens of conserved protein interactions should clarify mechanisms of genetic disease development, tolerance, and resilience. We therefore expect LECA protein interactions to offer direct insights into human disease genetics, and by similar logic, into genotype-phenotype relationships of other eukaryotes.

Slightly more than half of human genes date back to LECA. Where these conserved genes have been characterized, they are responsible for a large and diverse subset of major human diseases, spanning developmental disorders, cancers, chronic respiratory diseases, neurodegenerative conditions, and motor disorders (Figure 6A). For example, of the ~100 human genes linked with deafness, nearly ¾ trace back to LECA (Figure 6A). While some human diseases are "new," evolutionarily speaking, such as deficiency of the animal-specific pituitary hormone, many other diseases, such as ciliary dyskinesia, arise nearly entirely from genes in LECA OGs. In order to test the utility of our data for illuminating the biology of extant species, we next asked if the LECA interactome could be leveraged to predict human disease mechanisms and novel gene-disease relationships.

Approximately 500 LECA OGs are related to cilia, and among the most common ciliopathies are diseases of the kidney.[113] We identified a male infant with microcephaly, seizures, polycystic kidney disease, and end-stage renal failure, and whole-exome sequencing and pedigree analyses revealed a significant hemizygous, X-linked G>A variant in *EFHC2* (rs34729789, chr11:44148852:G>A) (Figures 6B–6D and Figure S3). Little is known of the function of *EFHC2*, though it and its paralog *EFHC1* (both belong to the same OG) encode proteins thought

Figure 6. LECA protein interactions suggest mechanisms of genetic disease, as for end-stage renal disease gene *EFHC2*, identified by whole-exome sequencing and confirmed to have a ciliary etiology

(A) Causal genes for human diseases are frequently ancient, as shown by plotting gene-disease relationships from OMIM, with each point representing a unique disease group with an associated number of genes (*x* axis) and age, determined as the percentage of genes in LECA OGs (*y* axis).

(B) Pedigree of the index family A4237. Squares represent males, circles females, black shading the affected proband individual A4237-22 included in whole-exome sequencing (WES), and white shading the unaffected parents and siblings.

(C) Summary of the phenotype and recessive disease-causing R133H *EFHC2* variant identified by WES.

(D) Location of arginine 133 in relation to *EFHC2* exon/intron (black/white) structure and DM10 protein domains (purple), and its deep evolutionary conservation.

(E) EFHC2-containing ciliary complex uncovered in the LECA interactome.

(F) Localization of GFP-EFHC2 to axonemes in *Xenopus* motile cilia.

(G) Introduction of the R133H mutation results in loss of ciliary localization of GFP-EFHC2, confirmed by co-labeling with membrane-RFP. Scale bar represents 10 μm.

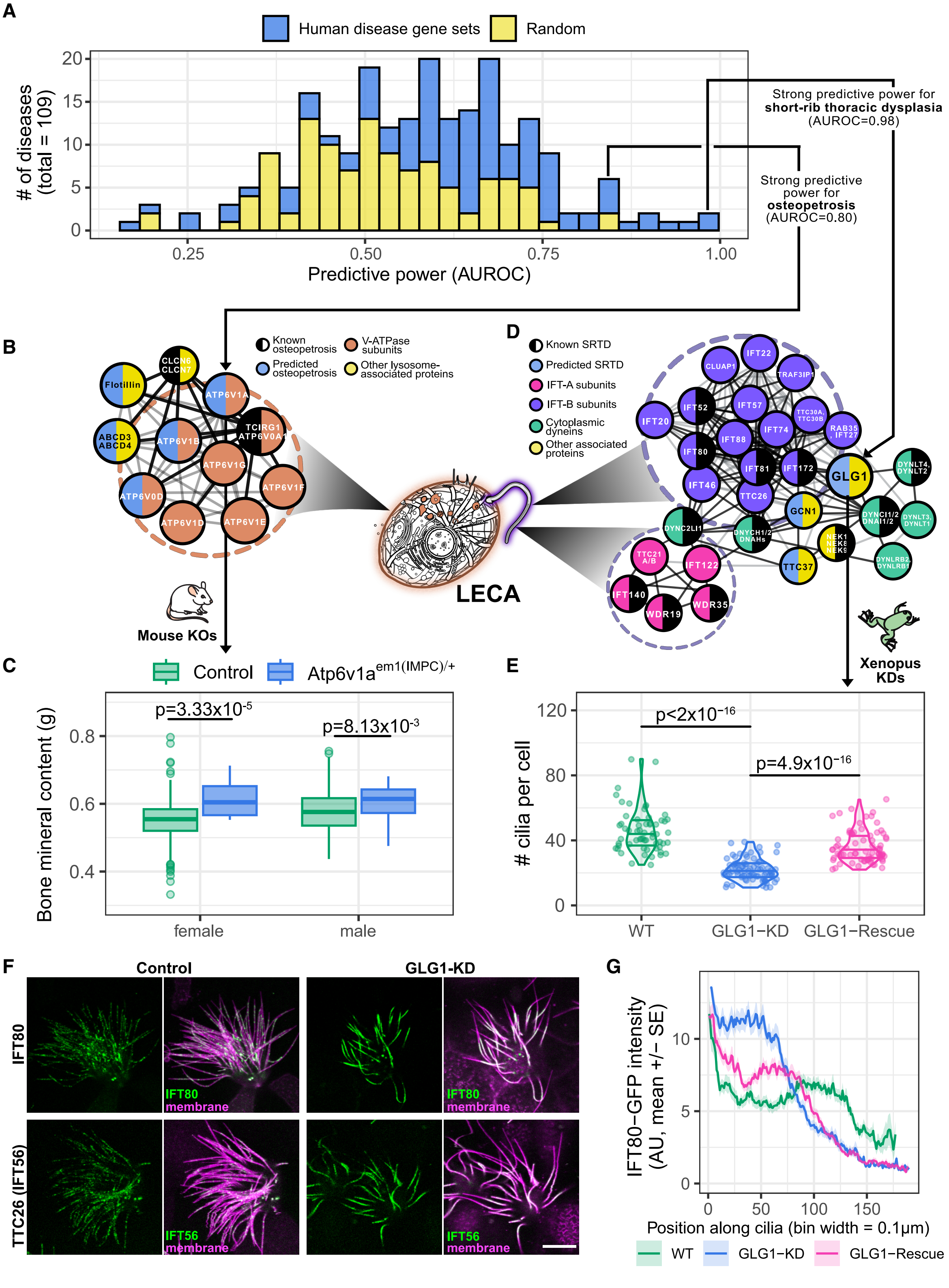

(legend on next page)

to be microtubule inner proteins (MIPs) that function specifically in motile cilia; loss of their orthologs in *Chlamydomonas* or *Tetrahymena* leads to defective ciliary axonemes and/or ciliary beating, but do not disrupt ciliogenesis.[114,115] Because motile cilia are absent from mammalian kidneys, the link between *EFHC2* and this patient's disease was surprising. We therefore examined our LECA interactome for insights.

We first noted that the patient's missense variant altered an arginine residue at position 133 to histidine, and this residue is conserved across Archaeplastida, Excavata, TSAR, and Amorphea (Figure 6D). Moreover, EFHC2 was closely and exclusively linked in our LECA interactome to other proteins involved in cilia motility (Figure 6E). We therefore examined the protein's localization in *Xenopus* multiciliated cells and found it to be very strongly localized to ciliary axonemes. By contrast, the disease-associated R133H variant failed to localize to cilia (Figures 6F and 6G), suggesting that defective ciliary localization of this protein contributed to ciliopathic kidney disease in the affected child.

This result prompted us to ask if other cluster proteins, also thought to function in motile cilia, might be implicated in kidney disease. Indeed, previous genomic analyses link both *PACRG*[116] and *TPPP*[117] to chronic kidney disease, with *PACRG* specifically linked to end-stage renal disease.[116] This combination of clinical data, the LECA interactome, and specific hypothesis testing in a vertebrate model organism thus links EFHC2 ciliary function to an end-stage renal disease for which the molecular etiology was previously unknown and underscores the power of our comparative evolution strategy.

Network propagation for systematic ranking of gene-disease relationships

We next sought to score potential disease-causative proteins systematically within our conserved interactome on a disease-by-disease basis. To this end, we used cross-validated network guilt-by-association[118] to predict novel gene-disease pairs for 109 diseases based on clinically validated genotype-to-phenotype relationships sourced from the OMIM database[119] (Figure 7A; see STAR Methods). We measured performance as areas under receiver operating characteristic curves (AUROC) and compared the predictive performance of known disease-associated gene sets versus that of random gene sets. As expected, random predictions yield AUROC scores around 0.5 (Figure 7A, yellow). In contrast, LECA-interactome predictions skewed to higher scores (Figure 7A, blue), and using a score threshold of 0.7, we made strong new disease candidate predictions for almost one-third of the diseases considered (~35 Mendelian disorders). As the LECA interactome should also predict phenotypes in other eukaryotes, we performed similar tests for yeast and *Chlamydomonas* mutant phenotypes and observed similarly strong performance (Figure S4). Notably, because network propagation operates on experimentally supported conserved interactions rather than on phylogenetic reconstruction alone, these phenotype predictions are robust to uncertainty in the precise evolutionary origin of individual gene families. Below, we discuss the prediction and validation in animals of two such novel protein associations.

Identification and validation of *ATP6V1A* as a novel candidate for osteopetrosis

Our LECA network propagation approach implicated several vacuolar-type H^+-ATPase (V-ATPase) proteins in the molecular etiology of osteopetrosis (AUROC ~0.8), a disorder in which bones grow abnormally and become overly dense[120] (Figure 7B). Given the role of V-ATPases in bone homeostasis by acidifying the space between osteoclasts and bone to help dissolve hydroxyapatite,[121] one might assume that disrupting many V-ATPase subunits increases bone density. However, only three subunits have been implicated in osteopetrosis in humans[119,122] or mice.[123] The remaining subunits instead display a remarkably broad spectrum of disease associations, including cutis laxa (loose skin) and renal tubular acidosis,[124] neurodegenerative disease, deafness,[125] Zimmermann-Laband syndrome,[126] and even osteoporosis (bone loss),[127] highlighting the need to elucidate the discrete molecular functions of specific V-ATPase subunits.

Our LECA network propagation approach gratifyingly made precise predictions, linking three specific subunits (ATP6V1A, ATP6V1B, and ATP6V0D) to osteopetrosis (Figure 7B). To confirm these predictions, we examined heterozygous

Figure 7. Guilt-by-association in the LECA interactome identifies *ATP6V1A* as causative for osteopetrosis and *GLG1* for short-rib thoracic dysplasia (SRTD)

(A) Guilt-by-association in the LECA PPI network correctly associates genes to human diseases for roughly $^1/_3$ of the 109 diseases tested, measured as the areas under receiver operating characteristic curves (AUROCs) of leave-one-out cross-validated predictions of known disease genes (light blue) versus random associations (yellow).

(B) PPI network of genes clinically linked to osteopetrosis (black half-discs; 3 additional genes lie outside this cluster), the highest-ranking new candidates (purple), and their interactions with other V-ATPase subunits that were not indicated for osteopetrosis (orange).

(C) For the top-scoring gene *ATP6V1A*, the bone mineral content is plotted for knockout (KO) mice with a heterozygous exon deletion in *ATP6V1A* ($n = 8$ for each sex, $n = 16$ total) compared to healthy control mice (female $n = 834$, male $n = 780$). Null mice show significantly increased bone density (see STAR Methods for statistical description), consistent with the clinical manifestation of osteopetrosis.

(D) The PPI network of genes clinically linked to SRTD (black half-discs) implicates *GLG1* (yellow) and suggests a ciliary role, based on interactions with intraflagellar trafficking IFT-A (blue) and IFT-B (purple) complexes, cytoplasmic dyneins and dynactins (green), and other interactors (gray).

(E) Morpholino knockdown (KD) of *GLG1* significantly reduced the number of cilia in *X. laevis* multi-ciliated cells (Bonferroni adjusted *t* test $p < 10^{-16}$, $n = 60$ control cells, 79 knockdown cells, and 76 rescue cells, 9 embryos per condition over 3 injection replicates) compared to uninjected control animals; rescue by co-injection with a non-targeted *GLG1* allele confirmed specificity.

(F) In control *Xenopus* multi-ciliated cells, IFT56-GFP and IFT80-GFP, two subunits of IFT-B, are distributed as particles along the ciliary axonemes. However, MO knockdown of *GLG1* leads to the accumulation of IFT-B proteins in the proximal region of axonemes. Scale bar represents 10 μm.

(G) This effect is quantified for IFT80-GFP for 3 cilia per cell for all cells analyzed in (E).

CRISPR-Cas9 knockouts of *ATP6V1A* (performed by the KOMP2 high-throughput mouse phenotyping site at the Baylor College of Medicine, see STAR Methods) and found these mice showed significantly increased bone mineral content (Figure 7C). The effect size was much stronger in female mice (p = 0.00003) than in male mice (p = 0.00813), echoing previous observations of sexual dimorphism in the body composition of mammals.[128,129] Despite the obvious lack of bones in the single-celled last eukaryotic ancestor, our examination of the protein interaction network of that organism nonetheless identified a specific mammalian phenotype with one specific subunit from among a large repertoire of closely related genes.

Ancient interactions suggest new candidate genes for a lethal human ciliopathy

Our highest scoring disease association (AUROC ~0.98) involved short-rib thoracic dysplasia (SRTD), a severe human ciliopathy characterized by skeletal abnormalities including dysplasia of the axial skeleton that often lethally impairs respiratory function.[130] The disease is strongly associated with proteins in intraflagellar transport (IFT), the system moving cargoes into and out of cilia, and this was reflected in our LECA interactome (Figure 7D). Our highest scoring non-IFT protein prediction, however, was the Golgi protein GLG1.[131] This was notable because mouse GLG1 mutants display rib defects similar to SRTD,[132] yet the protein has never been implicated in any aspect of ciliary biology.

We therefore explored the function of GLG1 in *Xenopus* multiciliated cells (MCCs) and found it predominantly localized to the Golgi in MCCs, as expected. We observed no localization at basal bodies or in cilia (not shown). Nonetheless, GLG1 knockdown resulted in significant loss of cilia from MCCs, an effect that was specific since it could be rescued by expression of GLG1-FLAG (Figure 7E). To ask if this ciliogenesis defect was related to IFT, we performed live imaging of GFP fusions to two IFT proteins. In normal cells, both markers labeled small punctae in axonemes of *Xenopus* MCCs, consistent with previous imaging of IFT in these cells.[133,134] By contrast, GLG1 knockdown cells displayed large accumulations of IFT proteins within axonemes (Figures 7F and 7G) that resemble those seen previously after disruption of IFT.[133,134]

Thus, analysis of the LECA interactome made a single, specific prediction of ciliary function for just one among the large array of Golgi-resident proteins, and that prediction was validated by experiments in *Xenopus*. These data provided new insights into the still obscure link between IFT and the Golgi[135,136] and, moreover, identified a plausible candidate gene for SRTD.

Conclusions

Conserved ancient protein interactions provide insight into the genetic basis of modern traits and diseases. In this work, we reconstructed macromolecular assemblies of ancient proteins that as yet have only been sparsely described. We defined a core set of likely LECA orthogroups, finding that slightly more than half of human genes can be traced back to this set. We integrated those data with >26,000 mass spectrometry proteomics experiments, capturing hundreds of millions of peptide measurements for hundreds of thousands of proteins in species spanning the eukaryotic tree. Using these data, we reconstructed a high-quality conserved LECA protein interactome. Improved probabilistic gain-loss and DTL models may refine membership at the margins of the LECA gene set but are unlikely to alter the major conserved complexes defined here, which are supported by direct proteomic evidence across multiple eukaryotic supergroups. This interaction network has formed the core of eukaryotic biology for nearly two billion years, and the dataset gives insights into known protein complexes and novel assemblies.

Consistent with our central premise that the most highly conserved protein assemblies will tend to be most critical for cell and organism function, the LECA interactome successfully predicts mechanisms of human disease and new gene-disease relationships. We presented evidence for a ciliary mechanism in human *EFHC2*-associated renal failure, identified the V-ATPase subunit ATP6V1A in the etiology of mammalian osteopetrosis, and demonstrated a role for the Golgi protein GLG1 in trafficking IFT-A proteins into cilia as a molecular mechanism for short-rib thoracic dysplasia. Given the richness of these datasets, we expect this approach to extend to traits and diseases in most eukaryotes, while providing insights into specific mechanisms involved due to being anchored in deeply conserved protein activities.

Limitations of the study

Our reconstruction of LECA's proteome and interactome has several limitations, including assumptions of the models used to infer ancestral genes, uneven taxonomic and experimental sampling, limited resolution of paralogs, and biases in CFMS favoring abundant, stable complexes. A discussion of these limitations is provided in the STAR Methods.

RESOURCE AVAILABILITY

Lead contact

Requests for further information and resources should be directed to and will be fulfilled by the lead contact, Edward M. Marcotte (marcotte@utexas.edu).

Materials availability

This study did not generate new materials.

Data and code availability

All raw and interpreted mass spectrometry data were deposited to the ProteomeXchange via the MassIVE repository under the identifiers in the Key Resources Table. Project data files are available at Zenodo (https://doi.org/10.5281/zenodo.10961422). Custom R, Python, Bash, and Perl scripts for analyses and figure generation are available at https://github.com/marcottelab/leca-proteomics and https://github.com/marcottelab/leca_notung_reconciliation under the DOIs given in the key resources table.

ACKNOWLEDGMENTS

The authors gratefully acknowledge support from the Tetrahymena stock center (Cornell University & Washington University in St. Louis), the Texas Advanced Computing Center (UT Austin) for computing resources, the Knockout Mouse Program (KOMP2) at the Baylor College of Medicine for *Atp6v1a* mice data, Johann Eberhart for rotifer cultures, the UTEX Culture Collection of Algae for algal samples, Maureen Stolzer for assistance with

reconciliation, and Angel Syrett and Elinor Marcotte for species illustrations. Research was funded by grants from the National Institute of General Medical Sciences (R35GM122480 to E.M.M. and F31GM143881 to R.M.C.), National Institute of Diabetes and Digestive and Kidney Diseases (R01DK068306 to F.H.), National Institute of Child Health and Human Development (R00HD092613 to K.D. and R01HD085901 to J.B.W. and E.M.M.), Army Research Office (W911NF-12-1-0390 to E.M.M.), and Welch Foundation (F-1515 to E.M.M.).

AUTHOR CONTRIBUTIONS

Design and co-supervision: R.M.C., J.B.W., and E.M.M. Proteomics experiments: O.P. aided by K.D. and J.C.L. Data analysis: R.M.C. aided by T.P.G., Z.T.A., A.M.B., M.L., K.D., C.D.M., D.Y., and D.D. and guided by E.M.M. Clinical genetics: S.S. and F.H. *Xenopus* experiments: C.L., T.P.G., and J.B.W. Manuscript initial draft: R.M.C., O.P., J.B.W., and E.M.M. All authors discussed results and contributed edits.

DECLARATION OF INTERESTS

The authors declare no competing interests.

STAR★METHODS

Detailed methods are provided in the online version of this paper and include the following:

- KEY RESOURCES TABLE
- EXPERIMENTAL MODEL AND STUDY PARTICIPANT DETAILS
- METHOD DETAILS
  - LECA gene set and resources
  - Mass spectrometry
  - Experimental analyses of candidate disease genes
- QUANTIFICATION AND STATISTICAL ANALYSIS
  - Computational analyses
  - Resources for disease analyses

SUPPLEMENTAL INFORMATION

Supplemental information can be found online at https://doi.org/10.1016/j.xgen.2026.101254.

Received: November 27, 2024
Revised: November 25, 2025
Accepted: May 5, 2026
Published: May 27, 2026

REFERENCES

1. Betts, H.C., Puttick, M.N., Clark, J.W., Williams, T.A., Donoghue, P.C.J., and Pisani, D. (2018). Integrated genomic and fossil evidence illuminates life's early evolution and eukaryote origin. Nat. Ecol. Evol. *2*, 1556–1562. https://doi.org/10.1038/s41559-018-0644-x.
2. Brocks, J.J., Nettersheim, B.J., Adam, P., Schaeffer, P., Jarrett, A.J.M., Güneli, N., Liyanage, T., van Maldegem, L.M., Hallmann, C., and Hope, J.M. (2023). Lost world of complex life and the late rise of the eukaryotic crown. Nature *618*, 767–773. https://doi.org/10.1038/s41586-023-06170-w.
3. Richards, T.A., Eme, L., Archibald, J.M., Leonard, G., Coelho, S.M., de Mendoza, A., Dessimoz, C., Dolezal, P., Fritz-Laylin, L.K., Gabaldón, T., et al. (2024). Reconstructing the last common ancestor of all eukaryotes. PLoS Biol. *22*, e3002917. https://doi.org/10.1371/journal.pbio.3002917.
4. Skejo, J., Garg, S.G., Gould, S.B., Hendriksen, M., Tria, F.D.K., Bremer, N., Franjević, D., Blackstone, N.W., and Martin, W.F. (2021). Evidence for a Syncytial Origin of Eukaryotes from Ancestral State Reconstruction. Genome Biol. Evol. *13*, evab096. https://doi.org/10.1093/gbe/evab096.
5. Bremer, N., Tria, F.D.K., Skejo, J., and Martin, W.F. (2023). The Ancestral Mitotic State: Closed Orthomitosis With Intranuclear Spindles in the Syncytial Last Eukaryotic Common Ancestor. Genome Biol. Evol. *15*, evad016. https://doi.org/10.1093/gbe/evad016.
6. Tromer, E.C., van Hooff, J.J.E., Kops, G.J.P.L., and Snel, B. (2019). Mosaic origin of the eukaryotic kinetochore. Proc. Natl. Acad. Sci. *116*, 12873–12882. https://doi.org/10.1073/pnas.1821945116.
7. Kontou, A., Herman, E.K., Field, M.C., Dacks, J.B., and Koumandou, V.L. (2022). Evolution of factors shaping the endoplasmic reticulum. Traffic *23*, 462–473. https://doi.org/10.1111/tra.12863.
8. Klute, M.J., Melançon, P., and Dacks, J.B. (2011). Evolution and Diversity of the Golgi. Cold Spring Harb. Perspect. Biol. *3*, a007849. https://doi.org/10.1101/cshperspect.a007849.
9. Field, M.C., and Dacks, J.B. (2009). First and last ancestors: reconstructing evolution of the endomembrane system with ESCRTs, vesicle coat proteins, and nuclear pore complexes. Curr. Opin. Cell Biol. *21*, 4–13. https://doi.org/10.1016/j.ceb.2008.12.004.
10. Mans, B.J., Anantharaman, V., Aravind, L., and Koonin, E.V. (2004). Comparative Genomics, Evolution and Origins of the Nuclear Envelope and Nuclear Pore Complex. Cell Cycle *3*, 1612–1637. https://doi.org/10.4161/cc.3.12.1316.
11. Wickstead, B., and Gull, K. (2011). The evolution of the cytoskeleton. J. Cell Biol. *194*, 513–525. https://doi.org/10.1083/jcb.201102065.
12. Richards, T.A., and Cavalier-Smith, T. (2005). Myosin domain evolution and the primary divergence of eukaryotes. Nature *436*, 1113–1118. https://doi.org/10.1038/nature03949.
13. Hodges, M.E., Scheumann, N., Wickstead, B., Langdale, J.A., and Gull, K. (2010). Reconstructing the evolutionary history of the centriole from protein components. J. Cell Sci. *123*, 1407–1413. https://doi.org/10.1242/jcs.064873.
14. Mitchell, D.R. (2017). Evolution of Cilia. Cold Spring Harb. Perspect. Biol. *9*, a028290. https://doi.org/10.1101/cshperspect.a028290.
15. Dacks, J.B., and Field, M.C. (2007). Evolution of the eukaryotic membrane-trafficking system: origin, tempo and mode. J. Cell Sci. *120*, 2977–2985. https://doi.org/10.1242/jcs.013250.
16. Gabaldón, T. (2018). Evolution of the Peroxisomal Proteome. In Proteomics of Peroxisomes: Identifying Novel Functions and Regulatory Networks Subcellular Biochemistry, L.A. del Río and M. Schrader, eds. (Springer), pp. 221–233. https://doi.org/10.1007/978-981-13-2233-4_9.
17. Müller, M., Mentel, M., van Hellemond, J.J., Henze, K., Woehle, C., Gould, S.B., Yu, R.-Y., van der Giezen, M., Tielens, A.G.M., and Martin, W.F. (2012). Biochemistry and Evolution of Anaerobic Energy Metabolism in Eukaryotes. Microbiol. Mol. Biol. Rev. *76*, 444–495. https://doi.org/10.1128/mmbr.05024-11.
18. Koonin, E.V. (2010). The Incredible Expanding Ancestor of Eukaryotes. Cell *140*, 606–608. https://doi.org/10.1016/j.cell.2010.02.022.
19. Deutekom, E.S., Snel, B., and van Dam, T.J.P. (2021). Benchmarking orthology methods using phylogenetic patterns defined at the base of Eukaryotes. Brief. Bioinform. 22, bbaa206.
20. O'Malley, M.A., Leger, M.M., Wideman, J.G., and Ruiz-Trillo, I. (2019). Concepts of the last eukaryotic common ancestor. Nat. Ecol. Evol. *3*, 338–344. https://doi.org/10.1038/s41559-019-0796-3.
21. Vosseberg, J., van Hooff, J.J.E., Marcet-Houben, M., van Vlimmeren, A., van Wijk, L.M., Gabaldón, T., and Snel, B. (2021). Timing the origin of eukaryotic cellular complexity with ancient duplications. Nat. Ecol. Evol. *5*, 92–100. https://doi.org/10.1038/s41559-020-01320-z.
22. Koumandou, V.L., Wickstead, B., Ginger, M.L., van der Giezen, M., Dacks, J.B., and Field, M.C. (2013). Molecular paleontology and complexity in the last eukaryotic common ancestor. Crit. Rev. Biochem. Mol. Biol. *48*, 373–396. https://doi.org/10.3109/10409238.2013.821444.

23. Hartwell, L.H., Hopfield, J.J., Leibler, S., and Murray, A.W. (1999). From molecular to modular cell biology. Nature *402*, C47–C52. https://doi.org/10.1038/35011540.

24. Jimenez-Sanchez, G., Childs, B., and Valle, D. (2001). Human disease genes. Nature *409*, 853–855. https://doi.org/10.1038/35057050.

25. Li, S., Armstrong, C.M., Bertin, N., Ge, H., Milstein, S., Boxem, M., Vidalain, P.-O., Han, J.-D.J., Chesneau, A., Hao, T., et al. (2004). A map of the interactome network of the metazoan C. elegans. Science *303*, 540–543. https://doi.org/10.1126/science.1091403.

26. Krogan, N.J., Cagney, G., Yu, H., Zhong, G., Guo, X., Ignatchenko, A., Li, J., Pu, S., Datta, N., Tikuisis, A.P., et al. (2006). Global landscape of protein complexes in the yeast Saccharomyces cerevisiae. Nature *440*, 637–643. https://doi.org/10.1038/nature04670.

27. Guruharsha, K.G., Rual, J.-F., Zhai, B., Mintseris, J., Vaidya, P., Vaidya, N., Beekman, C., Wong, C., Rhee, D.Y., Cenaj, O., et al. (2011). A protein complex network of Drosophila melanogaster. Cell *147*, 690–703. https://doi.org/10.1016/j.cell.2011.08.047.

28. Havugimana, P.C., Hart, G.T., Nepusz, T., Yang, H., Turinsky, A.L., Li, Z., Wang, P.I., Boutz, D.R., Fong, V., Phanse, S., et al. (2012). A census of human soluble protein complexes. Cell *150*, 1068–1081. https://doi.org/10.1016/j.cell.2012.08.011.

29. Wan, C., Borgeson, B., Phanse, S., Tu, F., Drew, K., Clark, G., Xiong, X., Kagan, O., Kwan, J., Bezginov, A., et al. (2015). Panorama of ancient metazoan macromolecular complexes. Nature *525*, 339–344. https://doi.org/10.1038/nature14877.

30. Drew, K., Wallingford, J.B., and Marcotte, E.M. (2021). hu.MAP 2.0: integration of over 15,000 proteomic experiments builds a global compendium of human multiprotein assemblies. Mol. Syst. Biol. *17*, e10016. https://doi.org/10.15252/msb.202010016.

31. Sae-Lee, W., McCafferty, C.L., Verbeke, E.J., Havugimana, P.C., Papoulas, O., McWhite, C.D., Houser, J.R., Vanuytsel, K., Murphy, G.J., Drew, K., et al. (2022). The protein organization of a red blood cell. Cell Rep. *40*, 111103. https://doi.org/10.1016/j.celrep.2022.111103.

32. Bassel, G.W., Gaudinier, A., Brady, S.M., Hennig, L., Rhee, S.Y., and De Smet, I. (2012). Systems Analysis of Plant Functional, Transcriptional, Physical Interaction, and Metabolic Networks. Plant Cell *24*, 3859–3875. https://doi.org/10.1105/tpc.112.100776.

33. McWhite, C.D., Papoulas, O., Drew, K., Cox, R.M., June, V., Dong, O.X., Kwon, T., Wan, C., Salmi, M.L., Roux, S.J., et al. (2020). A Pan-plant Protein Complex Map Reveals Deep Conservation and Novel Assemblies. Cell *181*, 460–474.e14. https://doi.org/10.1016/j.cell.2020.02.049.

34. Pan, J., Li, L.-P., You, Z.-H., Yu, C.-Q., Ren, Z.-H., and Guan, Y.-J. (2021). Prediction of Protein–Protein Interactions in Arabidopsis, Maize, and Rice by Combining Deep Neural Network With Discrete Hilbert Transform. Front. Genet. *12*, 745228.

35. Crozier, T.W.M., Tinti, M., Larance, M., Lamond, A.I., and Ferguson, M.A.J. (2017). Prediction of Protein Complexes in Trypanosoma brucei by Protein Correlation Profiling Mass Spectrometry and Machine Learning. Mol. Cell. Proteomics *16*, 2254–2267. https://doi.org/10.1074/mcp.O117.068122.

36. Hillier, C., Pardo, M., Yu, L., Bushell, E., Sanderson, T., Metcalf, T., Herd, C., Anar, B., Rayner, J.C., Billker, O., and Choudhary, J.S. (2019). Landscape of the Plasmodium Interactome Reveals Both Conserved and Species-Specific Functionality. Cell Rep. *28*, 1635–1647.e5. https://doi.org/10.1016/j.celrep.2019.07.019.

37. McGary, K.L., Park, T.J., Woods, J.O., Cha, H.J., Wallingford, J.B., and Marcotte, E.M. (2010). Systematic discovery of nonobvious human disease models through orthologous phenotypes. Proc. Natl. Acad. Sci. *107*, 6544–6549. https://doi.org/10.1073/pnas.0910200107.

38. Huerta-Cepas, J., Szklarczyk, D., Heller, D., Hernández-Plaza, A., Forslund, S.K., Cook, H., Mende, D.R., Letunic, I., Rattei, T., Jensen, L.J., et al. (2019). eggNOG 5.0: a hierarchical, functionally and phylogenetically annotated orthology resource based on 5090 organisms and 2502 viruses. Nucleic Acids Res. *47*, D309–D314. https://doi.org/10.1093/nar/gky1085.

39. Farris, J.S. (1977). Phylogenetic Analysis Under Dollo's Law. Syst. Biol. *26*, 77–88. https://doi.org/10.1093/sysbio/26.1.77.

40. Le Mercier, P., Bolleman, J., de Castro, E., Gasteiger, E., Bansal, P., Auchincloss, A.H., Boutet, E., Breuza, L., Casals-Casas, C., Estreicher, A., et al. (2022). SwissBioPics-an interactive library of cell images for the visualization of subcellular location data. Database. *2022*, baac026. https://doi.org/10.1093/database/baac026.

41. Martin, W., and Koonin, E.V. (2006). Introns and the origin of nucleus–cytosol compartmentalization. Nature *440*, 41–45. https://doi.org/10.1038/nature04531.

42. Alber, F., Dokudovskaya, S., Veenhoff, L.M., Zhang, W., Kipper, J., Devos, D., Suprapto, A., Karni-Schmidt, O., Williams, R., Chait, B.T., et al. (2007). The molecular architecture of the nuclear pore complex. Nature *450*, 695–701. https://doi.org/10.1038/nature06405.

43. Gabaldón, T., and Pittis, A.A. (2015). Origin and evolution of metabolic sub-cellular compartmentalization in eukaryotes. Biochimie *119*, 262–268. https://doi.org/10.1016/j.biochi.2015.03.021.

44. Ferrada, E., and Superti-Furga, G. (2022). A structure and evolutionary-based classification of solute carriers. iScience *25*, 105096. https://doi.org/10.1016/j.isci.2022.105096.

45. Leipe, D.D., Wolf, Y.I., Koonin, E.V., and Aravind, L. (2002). Classification and evolution of P-loop GTPases and related ATPases. J. Mol. Biol. *317*, 41–72. https://doi.org/10.1006/jmbi.2001.5378.

46. Li, J.B., Gerdes, J.M., Haycraft, C.J., Fan, Y., Teslovich, T.M., May-Simera, H., Li, H., Blacque, O.E., Li, L., Leitch, C.C., et al. (2004). Comparative Genomics Identifies a Flagellar and Basal Body Proteome that Includes the BBS5 Human Disease Gene. Cell *117*, 541–552. https://doi.org/10.1016/S0092-8674(04)00450-7.

47. Sigg, M.A., Menchen, T., Lee, C., Johnson, J., Jungnickel, M.K., Choksi, S.P., Garcia, G., Busengdal, H., Dougherty, G.W., Pennekamp, P., et al. (2017). Evolutionary Proteomics Uncovers Ancient Associations of Cilia with Signaling Pathways. Dev. Cell *43*, 744–762.e11. https://doi.org/10.1016/j.devcel.2017.11.014.

48. UniProt Consortium (2019). UniProt: a worldwide hub of protein knowledge. Nucleic Acids Res. *47*, D506–D515. https://doi.org/10.1093/nar/gky1049.

49. Domazet-Lošo, T., and Tautz, D. (2008). An Ancient Evolutionary Origin of Genes Associated with Human Genetic Diseases. Mol. Biol. Evol. *25*, 2699–2707. https://doi.org/10.1093/molbev/msn214.

50. Kondrashov, F.A., Koonin, E.V., Morgunov, I.G., Finogenova, T.V., and Kondrashova, M.N. (2006). Evolution of glyoxylate cycle enzymes in Metazoa: evidence of multiple horizontal transfer events and pseudogene formation. Biol. Direct *1*, 31. https://doi.org/10.1186/1745-6150-1-31.

51. Dunn, M.F., Ramírez-Trujillo, J.A., and Hernández-Lucas, I. (2009). Major roles of isocitrate lyase and malate synthase in bacterial and fungal pathogenesis. Microbiology *155*, 3166–3175. https://doi.org/10.1099/mic.0.030858-0.

52. Salido, E., Pey, A.L., Rodriguez, R., and Lorenzo, V. (2012). Primary hyperoxalurias: Disorders of glyoxylate detoxification. Biochim. Biophys. Acta *1822*, 1453–1464. https://doi.org/10.1016/j.bbadis.2012.03.004.

53. Kristensen, A.R., Gsponer, J., and Foster, L.J. (2012). A high-throughput approach for measuring temporal changes in the interactome. Nat. Methods *9*, 907–909. https://doi.org/10.1038/nmeth.2131.

54. Hein, M.Y., Hubner, N.C., Poser, I., Cox, J., Nagaraj, N., Toyoda, Y., Gak, I.A., Weisswange, I., Mansfeld, J., Buchholz, F., et al. (2015). A human interactome in three quantitative dimensions organized by stoichiometries and abundances. Cell *163*, 712–723. https://doi.org/10.1016/j.cell.2015.09.053.

55. Boldt, K., van Reeuwijk, J., Lu, Q., Koutroumpas, K., Nguyen, T.-M.T., Texier, Y., van Beersum, S.E.C., Horn, N., Willer, J.R., Mans, D.A., et al. (2016). An organelle-specific protein landscape identifies novel

diseases and molecular mechanisms. Nat. Commun. *7*, 11491. https://doi.org/10.1038/ncomms11491.

56. Huttlin, E.L., Bruckner, R.J., Paulo, J.A., Cannon, J.R., Ting, L., Baltier, K., Colby, G., Gebreab, F., Gygi, M.P., Parzen, H., et al. (2017). Architecture of the human interactome defines protein communities and disease networks. Nature *545*, 505–509. https://doi.org/10.1038/nature22366.

57. Gupta, G.D., Coyaud, É., Gonçalves, J., Mojarad, B.A., Liu, Y., Wu, Q., Gheiratmand, L., Comartin, D., Tkach, J.M., Cheung, S.W.T., et al. (2015). A Dynamic Protein Interaction Landscape of the Human Centrosome-Cilium Interface. Cell *163*, 1484–1499. https://doi.org/10.1016/j.cell.2015.10.065.

58. Youn, J.-Y., Dunham, W.H., Hong, S.J., Knight, J.D.R., Bashkurov, M., Chen, G.I., Bagci, H., Rathod, B., MacLeod, G., Eng, S.W.M., et al. (2018). High-Density Proximity Mapping Reveals the Subcellular Organization of mRNA-Associated Granules and Bodies. Mol. Cell *69*, 517–532.e11. https://doi.org/10.1016/j.molcel.2017.12.020.

59. Treiber, T., Treiber, N., Plessmann, U., Harlander, S., Daiß, J.-L., Eichner, N., Lehmann, G., Schall, K., Urlaub, H., and Meister, G. (2017). A Compendium of RNA-Binding Proteins that Regulate MicroRNA Biogenesis. Mol. Cell *66*, 270–284.e13. https://doi.org/10.1016/j.molcel.2017.03.014.

60. Giurgiu, M., Reinhard, J., Brauner, B., Dunger-Kaltenbach, I., Fobo, G., Frishman, G., Montrone, C., and Ruepp, A. (2019). CORUM: the comprehensive resource of mammalian protein complexes—2019. Nucleic Acids Res. *47*, D559–D563. https://doi.org/10.1093/nar/gky973.

61. Meldal, B.H.M., Bye-A-Jee, H., Gajdoš, L., Hammerová, Z., Horácková, A., Melicher, F., Perfetto, L., Pokorný, D., Lopez, M.R., Türková, A., et al. (2019). Complex Portal 2018: extended content and enhanced visualization tools for macromolecular complexes. Nucleic Acids Res. *47*, D550–D558. https://doi.org/10.1093/nar/gky1001.

62. Pons, P., and Latapy, M. (2005). Computing communities in large networks using random walks (long version). Preprint at arXiv. https://doi.org/10.48550/arXiv.physics/0512106.

63. Luck, K., Kim, D.-K., Lambourne, L., Spirohn, K., Begg, B.E., Bian, W., Brignall, R., Cafarelli, T., Campos-Laborie, F.J., Charloteaux, B., et al. (2020). A reference map of the human binary protein interactome. Nature *580*, 402–408. https://doi.org/10.1038/s41586-020-2188-x.

64. Kim, C.Y., Baek, S., Cha, J., Yang, S., Kim, E., Marcotte, E.M., Hart, T., and Lee, I. (2022). HumanNet v3: an improved database of human gene networks for disease research. Nucleic Acids Res. *50*, D632–D639. https://doi.org/10.1093/nar/gkab1048.

65. Bartolec, T.K., Vázquez-Campos, X., Norman, A., Luong, C., Johnson, M., Payne, R.J., Wilkins, M.R., Mackay, J.P., and Low, J.K.K. (2023). Cross-linking mass spectrometry discovers, evaluates, and corroborates structures and protein–protein interactions in the human cell. Proc. Natl. Acad. Sci. *120*, e2219418120. https://doi.org/10.1073/pnas.2219418120.

66. Koumandou, V.L., Dacks, J.B., Coulson, R.M.R., and Field, M.C. (2007). Control systems for membrane fusion in the ancestral eukaryote; evolution of tethering complexes and SM proteins. BMC Evol. Biol. *7*, 29. https://doi.org/10.1186/1471-2148-7-29.

67. Carvalho-Santos, Z., Azimzadeh, J., and Bettencourt-Dias, M. (2011). Tracing the origins of centrioles, cilia, and flagella. J. Cell Biol. *194*, 165–175. https://doi.org/10.1083/jcb.201011152.

68. Mani, J., Meisinger, C., and Schneider, A. (2016). Peeping at TOMs—Diverse Entry Gates to Mitochondria Provide Insights into the Evolution of Eukaryotes. Mol. Biol. Evol. *33*, 337–351. https://doi.org/10.1093/molbev/msv219.

69. D'Souza, Z., Taher, F.S., and Lupashin, V.V. (2020). Golgi inCOGnito: From vesicle tethering to human disease. Biochim. Biophys. Acta. Gen. Subj. *1864*, 129694. https://doi.org/10.1016/j.bbagen.2020.129694.

70. Morava, É., Guillard, M., Lefeber, D.J., and Wevers, R.A. (2009). Autosomal recessive cutis laxa syndrome revisited. Eur. J. Hum. Genet. *17*, 1099–1110. https://doi.org/10.1038/ejhg.2009.22.

71. Makhoul, C., Gosavi, P., and Gleeson, P.A. (2019). Golgi Dynamics: The Morphology of the Mammalian Golgi Apparatus in Health and Disease. Front. Cell Dev. Biol. *7*, 112.

72. Bonifacino, J.S., and Glick, B.S. (2004). The Mechanisms of Vesicle Budding and Fusion. Cell *116*, 153–166. https://doi.org/10.1016/S0092-8674(03)01079-1.

73. Gillingham, A.K., and Munro, S. (2019). Transport carrier tethering – how vesicles are captured by organelles. Curr. Opin. Cell Biol. *59*, 140–146. https://doi.org/10.1016/j.ceb.2019.04.010.

74. Schindler, C., Chen, Y., Pu, J., Guo, X., and Bonifacino, J.S. (2015). EARP is a multisubunit tethering complex involved in endocytic recycling. Nat. Cell Biol. *17*, 639–650. https://doi.org/10.1038/ncb3129.

75. Gershlick, D.C., Schindler, C., Chen, Y., and Bonifacino, J.S. (2016). TSSC1 is novel component of the endosomal retrieval machinery. Mol. Biol. Cell *27*, 2867–2878. https://doi.org/10.1091/mbc.E16-04-0209.

76. Balderhaar, H.J.k., and Ungermann, C. (2013). CORVET and HOPS tethering complexes – coordinators of endosome and lysosome fusion. J. Cell Sci. *126*, 1307–1316. https://doi.org/10.1242/jcs.107805.

77. Kim, J.J., Lipatova, Z., and Segev, N. (2016). TRAPP Complexes in Secretion and Autophagy. Front. Cell Dev. Biol. *4*, 20.

78. Wendler, F., Gillingham, A.K., Sinka, R., Rosa-Ferreira, C., Gordon, D.E., Franch-Marro, X., Peden, A.A., Vincent, J.-P., and Munro, S. (2010). A genome-wide RNA interference screen identifies two novel components of the metazoan secretory pathway. EMBO J. *29*, 304–314. https://doi.org/10.1038/emboj.2009.350.

79. Scrivens, P.J., Noueihed, B., Shahrzad, N., Hul, S., Brunet, S., and Sacher, M. (2011). C4orf41 and TTC-15 are mammalian TRAPP components with a role at an early stage in ER-to-Golgi trafficking. Mol. Biol. Cell *22*, 2083–2093. https://doi.org/10.1091/mbc.e10-11-0873.

80. Ungar, D., Oka, T., Vasile, E., Krieger, M., and Hughson, F.M. (2005). Subunit Architecture of the Conserved Oligomeric Golgi Complex. J. Biol. Chem. *280*, 32729–32735. https://doi.org/10.1074/jbc.M504590200.

81. Miller, V.J., Sharma, P., Kudlyk, T.A., Frost, L., Rofe, A.P., Watson, I.J., Duden, R., Lowe, M., Lupashin, V.V., and Ungar, D. (2013). Molecular Insights into Vesicle Tethering at the Golgi by the Conserved Oligomeric Golgi (COG) Complex and the Golgin TATA Element Modulatory Factor (TMF). J. Biol. Chem. *288*, 4229–4240. https://doi.org/10.1074/jbc.M112.426767.

82. Sethi, G., Shanmugam, M.K., Arfuso, F., and Kumar, A.P. (2018). Role of RNF20 in cancer development and progression – a comprehensive review. Biosci. Rep. *38*, BSR20171287. https://doi.org/10.1042/BSR20171287.

83. Fu, J., Liao, L., Balaji, K.S., Wei, C., Kim, J., and Peng, J. (2021). Epigenetic modification and a role for the E3 ligase RNF40 in cancer development and metastasis. Oncogene *40*, 465–474. https://doi.org/10.1038/s41388-020-01556-w.

84. Zhang, F., and Yu, X. (2011). WAC, a Functional Partner of RNF20/40, Regulates Histone H2B Ubiquitination and Gene Transcription. Mol. Cell *41*, 384–397. https://doi.org/10.1016/j.molcel.2011.01.024.

85. Totsukawa, G., Kaneko, Y., Uchiyama, K., Toh, H., Tamura, K., and Kondo, H. (2011). VCIP135 deubiquitinase and its binding protein, WAC, in p97ATPase-mediated membrane fusion. EMBO J. *30*, 3581–3593. https://doi.org/10.1038/emboj.2011.260.

86. Joachim, J., Jefferies, H.B.J., Razi, M., Frith, D., Snijders, A.P., Chakravarty, P., Judith, D., and Tooze, S.A. (2015). Activation of ULK Kinase and Autophagy by GABARAP Trafficking from the Centrosome Is Regulated by WAC and GM130. Mol. Cell *60*, 899–913. https://doi.org/10.1016/j.molcel.2015.11.018.

87. Sagan, L. (1967). On the origin of mitosing cells. J. Theor. Biol. *14*, 255–274. https://doi.org/10.1016/0022-5193(67)90079-3.

88. Martin, W., and Müller, M. (1998). The hydrogen hypothesis for the first eukaryote. Nature *392*, 37–41. https://doi.org/10.1038/32096.

89. Mills, D.B., Boyle, R.A., Daines, S.J., Sperling, E.A., Pisani, D., Donoghue, P.C.J., and Lenton, T.M. (2022). Eukaryogenesis and oxygen in

CellPress
OPEN ACCESS

Earth history. Nat. Ecol. Evol. *6*, 520–532. https://doi.org/10.1038/s41559-022-01733-y.

90. Yutin, N., Wolf, M.Y., Wolf, Y.I., and Koonin, E.V. (2009). The origins of phagocytosis and eukaryogenesis. Biol. Direct *4*, 9. https://doi.org/10.1186/1745-6150-4-9.

91. Martin, W.F., Tielens, A.G.M., Mentel, M., Garg, S.G., and Gould, S.B. (2017). The Physiology of Phagocytosis in the Context of Mitochondrial Origin. Microbiol. Mol. Biol. Rev. *81*, e00008-17. https://doi.org/10.1128/mmbr.00008-17.

92. Bremer, N., Tria, F.D.K., Skejo, J., Garg, S.G., and Martin, W.F. (2022). Ancestral State Reconstructions Trace Mitochondria But Not Phagocytosis to the Last Eukaryotic Common Ancestor. Genome Biol. Evol. *14*, evac079. https://doi.org/10.1093/gbe/evac079.

93. Maruyama, S., and Kim, E. (2013). A Modern Descendant of Early Green Algal Phagotrophs. Curr. Biol. *23*, 1081–1084. https://doi.org/10.1016/j.cub.2013.04.063.

94. Burns, J.A., Pittis, A.A., and Kim, E. (2018). Gene-based predictive models of trophic modes suggest Asgard archaea are not phagocytotic. Nat. Ecol. Evol. *2*, 697–704. https://doi.org/10.1038/s41559-018-0477-7.

95. Akıl, C., and Robinson, R.C. (2018). Genomes of Asgard archaea encode profilins that regulate actin. Nature *562*, 439–443. https://doi.org/10.1038/s41586-018-0548-6.

96. Akıl, C., Tran, L.T., Orhant-Prioux, M., Baskaran, Y., Manser, E., Blanchoin, L., and Robinson, R.C. (2020). Insights into the evolution of regulated actin dynamics via characterization of primitive gelsolin/cofilin proteins from Asgard archaea. Proc. Natl. Acad. Sci. *117*, 19904–19913. https://doi.org/10.1073/pnas.2009167117.

97. Rodrigues-Oliveira, T., Wollweber, F., Ponce-Toledo, R.I., Xu, J., Rittmann, S.K.-M.R., Klingl, A., Pilhofer, M., and Schleper, C. (2023). Actin cytoskeleton and complex cell architecture in an Asgard archaeon. Nature *613*, 332–339. https://doi.org/10.1038/s41586-022-05550-y.

98. Pollard, T.D. (2007). Regulation of Actin Filament Assembly by Arp2/3 Complex and Formins. Annu. Rev. Biophys. Biomol. Struct. *36*, 451–477. https://doi.org/10.1146/annurev.biophys.35.040405.101936.

99. Davidson, A.J., Amato, C., Thomason, P.A., and Insall, R.H. (2018). WASP family proteins and formins compete in pseudopod- and bleb-based migration. J. Cell Biol. *217*, 701–714. https://doi.org/10.1083/jcb.201705160.

100. Damiano-Guercio, J., Kurzawa, L., Mueller, J., Dimchev, G., Schaks, M., Nemethova, M., Pokrant, T., Brühmann, S., Linkner, J., Blanchoin, L., et al. (2020). Loss of Ena/VASP interferes with lamellipodium architecture, motility and integrin-dependent adhesion. eLife *9*, e55351. https://doi.org/10.7554/eLife.55351.

101. Edwards, M., Zwolak, A., Schafer, D.A., Sept, D., Dominguez, R., and Cooper, J.A. (2014). Capping protein regulators fine-tune actin assembly dynamics. Nat. Rev. Mol. Cell Biol. *15*, 677–689. https://doi.org/10.1038/nrm3869.

102. Gandhi, M., and Goode, B.L. (2013). Coronin: The Double-Edged Sword of Actin Dynamics. In Madame Curie Bioscience Database. [Internet] (Landes Bioscience).

103. Bolger-Munro, M., Choi, K., Cheung, F., Liu, Y.T., Dang-Lawson, M., Deretic, N., Keane, C., and Gold, M.R. (2021). The Wdr1-LIMK-Cofilin Axis Controls B Cell Antigen Receptor-Induced Actin Remodeling and Signaling at the Immune Synapse. Front. Cell Dev. Biol. *9*, 649433.

104. Pavlov, D., Muhlrad, A., Cooper, J., Wear, M., and Reisler, E. (2007). Actin Filament Severing by Cofilin. J. Mol. Biol. *365*, 1350–1358. https://doi.org/10.1016/j.jmb.2006.10.102.

105. Maniak, M., Rauchenberger, R., Albrecht, R., Murphy, J., and Gerisch, G. (1995). Coronin involved in phagocytosis: Dynamics of particle-induced relocalization visualized by a green fluorescent protein tag. Cell *83*, 915–924. https://doi.org/10.1016/0092-8674(95)90207-4.

106. Yan, M., Collins, R.F., Grinstein, S., and Trimble, W.S. (2005). Coronin-1 Function Is Required for Phagosome Formation. Mol. Biol. Cell *16*, 3077–3087. https://doi.org/10.1091/mbc.e04-11-0989.

107. Yan, M., Di Ciano-Oliveira, C., Grinstein, S., and Trimble, W.S. (2007). Coronin Function Is Required for Chemotaxis and Phagocytosis in Human Neutrophils1. J. Immunol. *178*, 5769–5778. https://doi.org/10.4049/jimmunol.178.9.5769.

108. Toshima, J., Toshima, J.Y., Martin, A.C., and Drubin, D.G. (2005). Phosphoregulation of Arp2/3-dependent actin assembly during receptor-mediated endocytosis. Nat. Cell Biol. *7*, 246–254. https://doi.org/10.1038/ncb1229.

109. Yu, B., Egbejimi, A., Dharmat, R., Xu, P., Zhao, Z., Long, B., Miao, H., Chen, R., Wensel, T.G., Cai, J., and Chen, Y. (2018). Phagocytosed photoreceptor outer segments activate mTORC1 in the retinal pigment epithelium. Sci. Signal. *11*, eaag3315. https://doi.org/10.1126/scisignal.aag3315.

110. Dürrwang, U., Fujita-Becker, S., Erent, M., Kull, F.J., Tsiavaliaris, G., Geeves, M.A., and Manstein, D.J. (2006). Dictyostelium myosin-IE is a fast molecular motor involved in phagocytosis. J. Cell Sci. *119*, 550–558. https://doi.org/10.1242/jcs.02774.

111. Barger, S.R., Reilly, N.S., Shutova, M.S., Li, Q., Maiuri, P., Heddleston, J.M., Mooseker, M.S., Flavell, R.A., Svitkina, T., Oakes, P.W., et al. (2019). Membrane-cytoskeletal crosstalk mediated by myosin-I regulates adhesion turnover during phagocytosis. Nat. Commun. *10*, 1249. https://doi.org/10.1038/s41467-019-09104-1.

112. Fili, N., and Toseland, C.P. (2019). Unconventional Myosins: How Regulation Meets Function. Int. J. Mol. Sci. *21*, 67. https://doi.org/10.3390/ijms21010067.

113. Bergmann, C., Guay-Woodford, L.M., Harris, P.C., Horie, S., Peters, D.J.M., and Torres, V.E. (2018). Polycystic kidney disease. Nat. Rev. Dis. Primers *4*, 50. https://doi.org/10.1038/s41572-018-0047-y.

114. Ikeda, K., Brown, J.A., Yagi, T., Norrander, J.M., Hirono, M., Eccleston, E., Kamiya, R., and Linck, R.W. (2003). Rib72, a Conserved Protein Associated with the Ribbon Compartment of Flagellar A-microtubules and Potentially Involved in the Linkage between Outer Doublet Microtubules. J. Biol. Chem. *278*, 7725. https://doi.org/10.1074/jbc.M210751200.

115. Stoddard, D., Zhao, Y., Bayless, B.A., Gui, L., Louka, P., Dave, D., Suryawanshi, S., Tomasi, R.F.-X., Dupuis-Williams, P., Baroud, C.N., et al. (2018). Tetrahymena RIB72A and RIB72B are microtubule inner proteins in the ciliary doublet microtubules. Mol. Biol. Cell *29*, 2566–2577. https://doi.org/10.1091/mbc.E18-06-0405.

116. Swan, E.J., Salem, R.M., Sandholm, N., Tarnow, L., Rossing, P., Lajer, M., Groop, P.H., Maxwell, A.P., and McKnight, A.J.; GENIE Consortium (2015). Genetic risk factors affecting mitochondrial function are associated with kidney disease in people with Type 1 diabetes. Diabet. Med. *32*, 1104–1109. https://doi.org/10.1111/dme.12763.

117. Han, M., Moon, S., Lee, S., Kim, K., An, W.J., Ryu, H., Kang, E., Ahn, J.-H., Sung, H.Y., Park, Y.S., et al. (2023). Novel Genetic Variants Associated with Chronic Kidney Disease Progression. J. Am. Soc. Nephrol. *34*, 857–875. https://doi.org/10.1681/ASN.0000000000000066.

118. Wang, P.I., and Marcotte, E.M. (2010). It's the machine that matters: Predicting gene function and phenotype from protein networks. J. Proteomics *73*, 2277–2289. https://doi.org/10.1016/j.jprot.2010.07.005.

119. Amberger, J.S., Bocchini, C.A., Schiettecatte, F., Scott, A.F., and Hamosh, A. (2015). OMIM.org: Online Mendelian Inheritance in Man (OMIM), an online catalog of human genes and genetic disorders. Nucleic Acids Res. *43*, D789–D798. https://doi.org/10.1093/nar/gku1205.

120. Tolar, J., Teitelbaum, S.L., and Orchard, P.J. (2004). Osteopetrosis. N. Engl. J. Med. *351*, 2839–2849. https://doi.org/10.1056/NEJMra040952.

121. Qin, A., Cheng, T.S., Pavlos, N.J., Lin, Z., Dai, K.R., and Zheng, M.H. (2012). V-ATPases in osteoclasts: Structure, function and potential inhibitors of bone resorption. Int. J. Biochem. Cell Biol. *44*, 1422–1435. https://doi.org/10.1016/j.biocel.2012.05.014.

122. Duan, X., Yang, S., Zhang, L., and Yang, T. (2018). V-ATPases and osteoclasts: ambiguous future of V-ATPases inhibitors in osteoporosis. Theranostics *8*, 5379–5399. https://doi.org/10.7150/thno.28391.

123. Nishi, T., Kawasaki-Nishi, S., and Forgac, M. (2003). Expression and function of the mouse V-ATPase d subunit isoforms. J. Biol. Chem. *278*, 46396–46402. https://doi.org/10.1074/jbc.M303924200.

124. Esmail, S., Kartner, N., Yao, Y., Kim, J.W., Reithmeier, R.A.F., and Manolson, M.F. (2018). Molecular mechanisms of cutis laxa– and distal renal tubular acidosis–causing mutations in V-ATPase a subunits, ATP6V0A2 and ATP6V0A4. J. Biol. Chem. *293*, 2787–2800. https://doi.org/10.1074/jbc.M117.818872.

125. Yuan, Y., Zhang, J., Chang, Q., Zeng, J., Xin, F., Wang, J., Zhu, Q., Wu, J., Lu, J., Guo, W., et al. (2014). De novo mutation in ATP6V1B2 impairs lysosome acidification and causes dominant deafness-onychodystrophy syndrome. Cell Res. *24*, 1370–1373. https://doi.org/10.1038/cr.2014.77.

126. Kortüm, F., Caputo, V., Bauer, C.K., Stella, L., Ciolfi, A., Alawi, M., Bocchinfuso, G., Flex, E., Paolacci, S., Dentici, M.L., et al. (2015). Mutations in KCNH1 and ATP6V1B2 cause Zimmermann-Laband syndrome. Nat. Genet. *47*, 661–667. https://doi.org/10.1038/ng.3282.

127. Duan, X., Liu, J., Zheng, X., Wang, Z., Zhang, Y., Hao, Y., Yang, T., and Deng, H. (2016). Deficiency of *ATP6V1H* Causes Bone Loss by Inhibiting Bone Resorption and Bone Formation through the TGF-β1 Pathway. Theranostics *6*, 2183–2195. https://doi.org/10.7150/thno.17140.

128. Öz, O.K., Zerwekh, J.E., Fisher, C., Graves, K., Nanu, L., Millsaps, R., and Simpson, E.R. (2000). Bone Has a Sexually Dimorphic Response to Aromatase Deficiency. J. Bone Miner. Res. *15*, 507–514. https://doi.org/10.1359/jbmr.2000.15.3.507.

129. Wells, J.C.K. (2007). Sexual dimorphism of body composition. Best Pract. Res. Clin. Endocrinol. Metab. *21*, 415–430. https://doi.org/10.1016/j.beem.2007.04.007.

130. Lai, B., Jiang, H., Gao, Y., and Zhou, X. (2024). Skeletal ciliopathy: pathogenesis and related signaling pathways. Mol. Cell. Biochem. *479*, 811–823. https://doi.org/10.1007/s11010-023-04765-5.

131. Steegmaier, M., Borges, E., Berger, J., Schwarz, H., and Vestweber, D. (1997). The E-selectin-ligand ESL-1 is located in the Golgi as well as on microvilli on the cell surface. J. Cell Sci. *110*, 687–694. https://doi.org/10.1242/jcs.110.6.687.

132. Yang, T., Mendoza-Londono, R., Lu, H., Tao, J., Li, K., Keller, B., Jiang, M.M., Shah, R., Chen, Y., Bertin, T.K., et al. (2010). E-selectin ligand–1 regulates growth plate homeostasis in mice by inhibiting the intracellular processing and secretion of mature TGF-β. J. Clin. Investig. *120*, 2474–2485. https://doi.org/10.1172/JCI42150.

133. Brooks, E.R., and Wallingford, J.B. (2012). Control of vertebrate intraflagellar transport by the planar cell polarity effector Fuz. J. Cell Biol. *198*, 37–45. https://doi.org/10.1083/jcb.201204072.

134. Toriyama, M., Lee, C., Taylor, S.P., Duran, I., Cohn, D.H., Bruel, A.-L., Tabler, J.M., Drew, K., Kelly, M.R., Kim, S., et al. (2016). The ciliopathy-associated CPLANE proteins direct basal body recruitment of intraflagellar transport machinery. Nat. Genet. *48*, 648–656. https://doi.org/10.1038/ng.3558.

135. Follit, J.A., Tuft, R.A., Fogarty, K.E., and Pazour, G.J. (2006). The intraflagellar transport protein IFT20 is associated with the Golgi complex and is required for cilia assembly. Mol. Biol. Cell *17*, 3781–3792. https://doi.org/10.1091/mbc.e06-02-0133.

136. Quidwai, T., Wang, J., Hall, E.A., Petriman, N.A., Leng, W., Kiesel, P., Wells, J.N., Murphy, L.C., Keighren, M.A., Marsh, J.A., et al. (2021). A WDR35-dependent coat protein complex transports ciliary membrane cargo vesicles to cilia. eLife *10*, e69786. https://doi.org/10.7554/eLife.69786.

137. Drew, K., Lee, C., Cox, R.M., Dang, V., Devitt, C.C., McWhite, C.D., Papoulas, O., Huizar, R.L., Marcotte, E.M., and Wallingford, J.B. (2020). A systematic, label-free method for identifying RNA-associated proteins in vivo provides insights into vertebrate ciliary beating machinery. Dev. Biol. *467*, 108–117. https://doi.org/10.1016/j.ydbio.2020.08.008.

138. Boeckmann, B., Marcet-Houben, M., Rees, J.A., Forslund, K., Huerta-Cepas, J., Muffato, M., Yilmaz, P., Xenarios, I., Bork, P., Lewis, S.E., et al. (2015). Quest for Orthologs Entails Quest for Tree of Life: In Search of the Gene Stream. Genome Biol. Evol. *7*, 1988–1999. https://doi.org/10.1093/gbe/evv121.

139. Altenhoff, A.M., Garrayo-Ventas, J., Cosentino, S., Emms, D., Glover, N.M., Hernández-Plaza, A., Nevers, Y., Sundesha, V., Szklarczyk, D., Fernández, J.M., et al. (2020). The Quest for Orthologs benchmark service and consensus calls in 2020. Nucleic Acids Res. *48*, W538–W545. https://doi.org/10.1093/nar/gkaa308.

140. Cantalapiedra, C.P., Hernández-Plaza, A., Letunic, I., Bork, P., and Huerta-Cepas, J. (2021). eggNOG-mapper v2: Functional Annotation, Orthology Assignments, and Domain Prediction at the Metagenomic Scale. Mol. Biol. Evol. *38*, 5825–5829. https://doi.org/10.1093/molbev/msab293.

141. Csűös, M. (2010). Count: evolutionary analysis of phylogenetic profiles with parsimony and likelihood. Bioinformatics *26*, 1910–1912. https://doi.org/10.1093/bioinformatics/btq315.

142. Boeckmann, B., Dylus, D., Moretti, S., Altenhoff, A., Train, C.-M., Kriventseva, E., Bougueleret, L., Xenarios, I., Privman, E., Gabaldon, T., et al. (2017). Taxon sampling unequally affects individual nodes in a phylogenetic tree: consequences for model gene tree construction in SwissTree. Preprint at at bioRxiv. https://doi.org/10.1101/181966.

143. Kluge, A.G., and Farris, J.S. (1969). Quantitative Phyletics and the Evolution of Anurans. Syst. Biol. *18*, 1–32. https://doi.org/10.1093/sysbio/18.1.1.

144. Chen, K., Durand, D., and Farach-Colton, M. (2000). NOTUNG: a program for dating gene duplications and optimizing gene family trees. J. Comput. Biol. *7*, 429–447. https://doi.org/10.1089/106652700750050871.

145. Nehrt, N.L., Clark, W.T., Radivojac, P., and Hahn, M.W. (2011). Testing the Ortholog Conjecture with Comparative Functional Genomic Data from Mammals. PLoS Comput. Biol. *7*, e1002073. https://doi.org/10.1371/journal.pcbi.1002073.

146. Altenhoff, A.M., Studer, R.A., Robinson-Rechavi, M., and Dessimoz, C. (2012). Resolving the Ortholog Conjecture: Orthologs Tend to Be Weakly, but Significantly, More Similar in Function than Paralogs. PLoS Comput. Biol. *8*, e1002514. https://doi.org/10.1371/journal.pcbi.1002514.

147. Altenhoff, A.M., and Dessimoz, C. (2012). Inferring orthology and paralogy. Methods Mol. Biol. *855*, 259–279. https://doi.org/10.1007/978-1-61779-582-4_9.

148. Altenhoff, A.M., Boeckmann, B., Capella-Gutierrez, S., Dalquen, D.A., DeLuca, T., Forslund, K., Huerta-Cepas, J., Linard, B., Pereira, C., Pryszcz, L.P., et al. (2016). Standardized benchmarking in the quest for orthologs. Nat. Methods *13*, 425–430. https://doi.org/10.1038/nmeth.3830.

149. Nevers, Y., Jones, T.E.M., Jyothi, D., Yates, B., Ferret, M., Portell-Silva, L., Codo, L., Cosentino, S., Marcet-Houben, M., Vlasova, A., et al. (2022). The Quest for Orthologs orthology benchmark service in 2022. Nucleic Acids Res. *50*, W623–W632. https://doi.org/10.1093/nar/gkac330.

150. Daugherty, M.D., and Malik, H.S. (2012). Rules of Engagement: Molecular Insights from Host-Virus Arms Races. Annu. Rev. Genet. *46*, 677–700. https://doi.org/10.1146/annurev-genet-110711-155522.

151. Gilad, Y., Oshlack, A., Smyth, G.K., Speed, T.P., and White, K.P. (2006). Expression profiling in primates reveals a rapid evolution of human transcription factors. Nature *440*, 242–245. https://doi.org/10.1038/nature04559.

152. de Mendoza, A., and Sebé-Pedrós, A. (2019). Origin and evolution of eukaryotic transcription factors. Curr. Opin. Genet. Dev. *58–59*, 25–32. https://doi.org/10.1016/j.gde.2019.07.010.

153. Sloan, D.B., Alverson, A.J., Chuckalovcak, J.P., Wu, M., McCauley, D.E., Palmer, J.D., and Taylor, D.R. (2012). Rapid Evolution of Enormous, Multichromosomal Genomes in Flowering Plant Mitochondria with Exceptionally High Mutation Rates. PLoS Biol. *10*, e1001241. https://doi.org/10.1371/journal.pbio.1001241.

154. Luo, A., and Ho, S.Y.W. (2018). The molecular clock and evolutionary timescales. Biochem. Soc. Trans. *46*, 1183–1190. https://doi.org/10.1042/BST20180186.

155. Gould, S.J. (1970). Dollo on Dollo's law: Irreversibility and the status of evolutionary laws. J. Hist. Biol. *3*, 189–212. https://doi.org/10.1007/BF00137351.

156. Rogozin, I.B., Wolf, Y.I., Babenko, V.N., and Koonin, E.V. (2006). Dollo parsimony and the reconstruction of genome evolution. In Parsimony, Phylogeny, and Genomics, V.A. Albert, ed. (Oxford University Press)). https://doi.org/10.1093/acprof:oso/9780199297306.003.0011.

157. Goldberg, E.E., and Igić, B. (2008). ON PHYLOGENETIC TESTS OF IRREVERSIBLE EVOLUTION. Evolution *62*, 2727–2741. https://doi.org/10.1111/j.1558-5646.2008.00505.x.

158. Van Etten, J., and Bhattacharya, D. (2020). Horizontal Gene Transfer in Eukaryotes: Not if, but How Much? Trends Genet. *36*, 915–925. https://doi.org/10.1016/j.tig.2020.08.006.

159. Martin, W., and Borst, P. (2003). Secondary loss of chloroplasts in trypanosomes. Proc. Natl. Acad. Sci. *100*, 765–767. https://doi.org/10.1073/pnas.0437776100.

160. Hannaert, V., Saavedra, E., Duffieux, F., Szikora, J.-P., Rigden, D.J., Michels, P.A.M., and Opperdoes, F.R. (2003). Plant-like traits associated with metabolism of Trypanosoma parasites. Proc. Natl. Acad. Sci. USA *100*, 1067–1071. https://doi.org/10.1073/pnas.0335769100.

161. Maruyama, S., Matsuzaki, M., Misawa, K., and Nozaki, H. (2009). Cyanobacterial contribution to the genomes of the plastid-lacking protists. BMC Evol. Biol. *9*, 197. https://doi.org/10.1186/1471-2148-9-197.

162. Matsuzaki, M., Misumi, O., Shin-i, T., Maruyama, S., Takahara, M., Miyagishima, S.Y., Mori, T., Nishida, K., Yagisawa, F., Nishida, K., et al. (2004). Genome sequence of the ultrasmall unicellular red alga Cyanidioschyzon merolae 10D. Nature *428*, 653–657. https://doi.org/10.1038/nature02398.

163. Reyes-Prieto, A., and Bhattacharya, D. (2007). Phylogeny of Calvin cycle enzymes supports Plantae monophyly. Mol. Phylogenet. Evol. *45*, 384–391. https://doi.org/10.1016/j.ympev.2007.02.026.

164. Bhattacharya, D., Yoon, H.S., and Hackett, J.D. (2004). Photosynthetic eukaryotes unite: endosymbiosis connects the dots. Bioessays *26*, 50–60. https://doi.org/10.1002/bies.10376.

165. Burki, F., Roger, A.J., Brown, M.W., and Simpson, A.G.B. (2020). The New Tree of Eukaryotes. Trends Ecol. Evol. *35*, 43–55. https://doi.org/10.1016/j.tree.2019.08.008.

166. Hampl, V., Hug, L., Leigh, J.W., Dacks, J.B., Lang, B.F., Simpson, A.G.B., and Roger, A.J. (2009). Phylogenomic analyses support the monophyly of Excavata and resolve relationships among eukaryotic "supergroups.". Proc. Natl. Acad. Sci. *106*, 3859–3864. https://doi.org/10.1073/pnas.0807880106.

167. Parfrey, L.W., Grant, J., Tekle, Y.I., Lasek-Nesselquist, E., Morrison, H.G., Sogin, M.L., Patterson, D.J., and Katz, L.A. (2010). Broadly Sampled Multigene Analyses Yield a Well-Resolved Eukaryotic Tree of Life. Syst. Biol. *59*, 518–533. https://doi.org/10.1093/sysbio/syq037.

168. Williamson, K., Eme, L., Baños, H., McCarthy, C.G.P., Susko, E., Kamikawa, R., Orr, R.J.S., Muñoz-Gómez, S.A., Minh, B.Q., Simpson, A.G.B., and Roger, A.J. (2025). A robustly rooted tree of eukaryotes reveals their excavate ancestry. Nature *640*, 974–981. https://doi.org/10.1038/s41586-025-08709-5.

169. Mishra, S., and Lercher, M.J. (2025). Horizontal Gene Transfer Inference: Gene Presence-Absence Outperforms Gene Trees. Mol. Biol. Evol. *42*, msaf166. https://doi.org/10.1093/molbev/msaf166.

170. Raychowdhury, M.K., McLaughlin, M., Ramos, A.J., Montalbetti, N., Bouley, R., Ausiello, D.A., and Cantiello, H.F. (2005). Characterization of Single Channel Currents from Primary Cilia of Renal Epithelial Cells. J. Biol. Chem. *280*, 34718–34722. https://doi.org/10.1074/jbc.M507793200.

171. McCafferty, C.L., Papoulas, O., Jordan, M.A., Hoogerbrugge, G., Nichols, C., Pigino, G., Taylor, D.W., Wallingford, J.B., and Marcotte, E.M. (2022). Integrative modeling reveals the molecular architecture of the intraflagellar transport A (IFT-A) complex. eLife *11*, e81977. https://doi.org/10.7554/eLife.81977.

172. Dentler, W.L. (1995). Chapter 3 Isolation of Cilia from Tetrahymena thermophila. In Methods in Cell Biology (Elsevier), pp. 13–15. https://doi.org/10.1016/S0091-679X(08)60784-0.

173. Mallam, A.L., Sae-Lee, W., Schaub, J.M., Tu, F., Battenhouse, A., Jang, Y.J., Kim, J., Wallingford, J.B., Finkelstein, I.J., Marcotte, E.M., and Drew, K. (2019). Systematic Discovery of Endogenous Human Ribonucleoprotein Complexes. Cell Rep. *29*, 1351–1368.e5. https://doi.org/10.1016/j.celrep.2019.09.060.

174. McWhite, C.D., Papoulas, O., Drew, K., Dang, V., Leggere, J.C., Sae-Lee, W., and Marcotte, E.M. (2021). Co-fractionation/mass spectrometry to identify protein complexes. STAR Protoc. *2*, 100370. https://doi.org/10.1016/j.xpro.2021.100370.

175. Groza, T., Gomez, F.L., Mashhadi, H.H., Muñoz-Fuentes, V., Gunes, O., Wilson, R., Cacheiro, P., Frost, A., Keskivali-Bond, P., Vardal, B., et al. (2023). The International Mouse Phenotyping Consortium: comprehensive knockout phenotyping underpinning the study of human disease. Nucleic Acids Res. *51*, D1038–D1045. https://doi.org/10.1093/nar/gkac972.

176. Birling, M.-C., Yoshiki, A., Adams, D.J., Ayabe, S., Beaudet, A.L., Bottomley, J., Bradley, A., Brown, S.D.M., Bürger, A., Bushell, W., et al. (2021). A resource of targeted mutant mouse lines for 5,061 genes. Nat. Genet. *53*, 416–419. https://doi.org/10.1038/s41588-021-00825-y.

177. Kurbatova, N., Mason, J.C., Morgan, H., Meehan, T.F., and Karp, N.A. (2015). PhenStat: A Tool Kit for Standardized Analysis of High Throughput Phenotypic Data. PLoS One *10*, e0131274. https://doi.org/10.1371/journal.pone.0131274.

178. Park, T.J., Gray, R.S., Sato, A., Habas, R., and Wallingford, J.B. (2005). Subcellular Localization and Signaling Properties of Dishevelled in Developing Vertebrate Embryos. Curr. Biol. *15*, 1039–1044. https://doi.org/10.1016/j.cub.2005.04.062.

179. Huizar, R.L., Lee, C., Boulgakov, A.A., Horani, A., Tu, F., Marcotte, E.M., Brody, S.L., and Wallingford, J.B. (2018). A liquid-like organelle at the root of motile ciliopathy. eLife *7*, e38497. https://doi.org/10.7554/eLife.38497.

180. Lee, C., Cox, R.M., Papoulas, O., Horani, A., Drew, K., Devitt, C.C., Brody, S.L., Marcotte, E.M., and Wallingford, J.B. (2020). Functional partitioning of a liquid-like organelle during assembly of axonemal dyneins. eLife *9*, e58662. https://doi.org/10.7554/eLife.58662.

181. Fisher, M., James-Zorn, C., Ponferrada, V., Bell, A.J., Sundararaj, N., Segerdell, E., Chaturvedi, P., Bayyari, N., Chu, S., Pells, T., et al. (2023). Xenbase: key features and resources of the Xenopus model organism knowledgebase. Genetics *224*, iyad018. https://doi.org/10.1093/genetics/iyad018.

182. Kwon, T., Choi, H., Vogel, C., Nesvizhskii, A.I., and Marcotte, E.M. (2011). MSblender: A Probabilistic Approach for Integrating Peptide Identifications from Multiple Database Search Engines. J. Proteome Res. *10*, 2949–2958. https://doi.org/10.1021/pr2002116.

183. Olson, R.S., Bartley, N., Urbanowicz, R.J., and Moore, J.H. (2016). Evaluation of a Tree-based Pipeline Optimization Tool for Automating Data Science. In Proceedings of the Genetic and Evolutionary Computation Conference 2016 (ACM)), pp. 485–492. https://doi.org/10.1145/2908812.2908918.

184. Newman, M.E.J., and Girvan, M. (2004). Finding and evaluating community structure in networks. Phys. Rev. E *69*, 026113. https://doi.org/10.1103/PhysRevE.69.026113.

185. Meysman, P., Titeca, K., Eyckerman, S., Tavernier, J., Goethals, B., Martens, L., Valkenborg, D., and Laukens, K. (2017). Protein complex analysis: From raw protein lists to protein interaction networks. Mass Spectrom. Rev. *36*, 600–614. https://doi.org/10.1002/mas.21485.

186. Turco, G., Chang, C., Wang, R.Y., Kim, G., Stoops, E.H., Richardson, B., Sochat, V., Rust, J., Oughtred, R., Thayer, N., et al. (2023). Global analysis of the yeast knockout phenome. Sci. Adv. *9*, eadg5702. https://doi.org/10.1126/sciadv.adg5702.

187. Merchant, S.S., Prochnik, S.E., Vallon, O., Harris, E.H., Karpowicz, S.J., Witman, G.B., Terry, A., Salamov, A., Fritz-Laylin, L.K., Maréchal-Drouard, L., et al. (2007). The Chlamydomonas genome reveals the evolution of key animal and plant functions. Science *318*, 245–250. https://doi.org/10.1126/science.1143609.

CellPress
OPEN ACCESS

STAR★METHODS

KEY RESOURCES TABLE

REAGENT or RESOURCE	SOURCE	IDENTIFIER
Biological samples		
Euglena gracilis	UTEX Culture Collection of Algae	UTEX 753
Tetrahymena thermophila	Tetrahymena Stock Center	SB175 (SD01508)
Pig trachea (Sus scrofa)	Sierra for Medical Science	N/A
Diatom (Phaeodactylum tricornutum)	UTEX Culture Collection of Algae	UTEX 646
Rotifer (Brachionus rotundiformis)	Eberhart lab, UT Austin	S-type
Mass spectrometry data		
Euglena gracilis	This work	PRIDE: PXD050669
Tetrahymena thermophila	This work	PRIDE: PXD050671, PXD050672, PXD050674
Pig (*Sus scrofa*)	This work	PRIDE: PXD041980
Diatom (*Phaeodactylum tricornutum*)	This work	PRIDE: PXD050670
Rotifer (*Brachionus rotundiformis*)	This work	PRIDE: PXD050673
Arabidopsis thaliana	McWhite et al.[33]	PRIDE: PXD013264, PXD013321, PXD014617
Broccoli (*Brassica oleracea*)	McWhite et al.[33]	PRIDE: PXD013281, PXD013322, PXD013282
Chlamydomonas reinhardtii	McWhite et al.[33]	PRIDE: PXD013369, PXD013735
Coconut (*Cocos nucifera*)	McWhite et al.[33]	PRIDE: PXD012865
Fern (*Ceratopteris richardii*)	McWhite et al.[33]	PRIDE: PXD013320
Hemp (*Cannabis sativa*)	McWhite et al.[33]	PRIDE: PXD012969
Maize (*Zea mays*)	McWhite et al.[33]	PRIDE: PXD012810
Quinoa (*Chenopodium quinoa*)	McWhite et al.[33]	PRIDE: PXD013080
Rice (*Oryza sativa*)	McWhite et al.[33]	PRIDE: PXD013213
Selaginella moellendorffii	McWhite et al.[33]	PRIDE: PXD013093
Soy (*Glycine max*)	McWhite et al.[33]	PRIDE: PXD013198, PXD013704
Tomato (*Solanum lycopersicum*)	McWhite et al.[33]	PRIDE: PXD013004
Wheat (*Triticum aestivum*)	McWhite et al.[33]	PRIDE: PXD013214, PXD013280, PXD013300
Plasmodium berghei	Hillier et al.[36]	PRIDE: PXD009039
Plasmodium falciparum	Hillier et al.[36]	PRIDE: PXD009039
Plasmodium knowlesi	Hillier et al.[36]	PRIDE: PXD009039
Trypanosoma brucei	Crozier et al.[35]	PRIDE: PXD005968
Human (*Homo sapiens*)	Wan et al.[29]	PRIDE: PXD002322, PXD002328
Worm (*Caenorhabditis elegans*)	Wan et al.[29]	PRIDE: PXD002319
Slime mold (*Dictyostelium discoideum*)	Wan et al.[29]	PRIDE: PXD002320
Fly (*Drosophila melanogaster*)	Wan et al.[29]	PRIDE: PXD002321
Mouse (*Mus musculus*)	Wan et al.[29]	PRIDE: PXD002323
Sea anemone (*Nematostella vectensis*)	Wan et al.[29]	PRIDE: PXD002324
Sea urchin (*Strongylocentrotus purpuratus*)	Wan et al.[29]	PRIDE: PXD002325
African clawed frog (*Xenopus laevis*)	Drew et al.[137]; Wan et al.[29]	PRIDE: PXD017650, PXD017659; PXD002326
Yeast (*Saccharomyces cerevisiae*)	Wan et al.[29]	PRIDE: PXD002327
Software and algorithms		
Orthogroup inference	eggNOG v2.0.5	https://github.com/eggnogdb/eggnog-mapper/releases

(*Continued on next page*)

Continued

REAGENT or RESOURCE	SOURCE	IDENTIFIER
Reference database construction	This paper	https://github.com/marcottelab/leca-proteomics (scripts/concat_ortho_proteins.py), available at https://doi.org/10.5281/zenodo.19701798
Peptide identification	MSblender	https://github.com/marcottelab/MSblender, available at https://doi.org/10.5281/zenodo.19698770
Ancestral genome inference	Count	http://www.iro.umontreal.ca/~csuros/gene_content/count.html
Feature extraction (CFMS data)	McWhite et al.[33]	https://github.com/marcottelab/protein_complex_maps/tree/master/protein_complex_maps/features/ExtractFeatures/canned_scripts/extract_features.py, available at https://doi.org/10.5281/zenodo.19701816
Feature integration (APMS data)	hu.MAP 2.0	https://humap2.proteincomplexes.org/download
	This paper	https://github.com/marcottelab/leca-proteomics (notebooks/map_entrez_to_eggnog.ipynb), available at https://doi.org/10.5281/zenodo.19701798
Pairwise protein interaction labels	This paper	https://github.com/marcottelab/leca-proteomics (scripts/label_featmat.py), available at https://doi.org/10.5281/zenodo.19701798
Model optimization	TPOT 0.11.7	http://epistasislab.github.io/tpot/
	This paper	https://github.com/marcottelab/leca-proteomics (scripts/run_tpot.py), available at https://doi.org/10.5281/zenodo.19701798
Feature/model selection	This paper	https://github.com/marcottelab/leca-proteomics (scripts/select_features.py), available at https://doi.org/10.5281/zenodo.19701798
Final model generation/assessment	This paper	https://github.com/marcottelab/leca-proteomics (scripts/predict_ppis.py), available at https://doi.org/10.5281/zenodo.19701798
Protein complex detection	This paper	https://github.com/marcottelab/leca-proteomics (scripts/detect_communities.py), available at https://doi.org/10.5281/zenodo.19701798
Phylogenetic reconciliation	Notung	https://www.cs.cmu.edu/~durand/Notung/and supporting code available at https://doi.org/10.5281/zenodo.19701837

EXPERIMENTAL MODEL AND STUDY PARTICIPANT DETAILS

The present study includes proteomics data from 31 species of organisms, detailed in the Key Resources Table. Additional cell biological data were collected from *Xenopus laevis* and were conducted in accordance with the animal protocol AUP-2024-00130 and the animal ethics guidelines of the University of Texas at Austin. Phenotypic and genetic data were obtained by authors S.S. and F.H. for one individual. This study was approved by the institutional review board (IRB) of Boston Children's Hospital (protocol IRB-P00006200) as well as the institutional review boards of institutions where authors S.S. and F.H. have recruited families.

METHOD DETAILS

LECA gene set and resources

Resources for inferring the LECA gene set

Reference proteomes for 156 species (122 eukaryotes, 7 archaea, 27 bacteria; see Zenodo repository) were downloaded from the UniProt database along with the corresponding reference species tree[138] for the parsimony analysis (Figure S5). This species tree and set of organisms were selected because they span the tree of life and serve as the gold standards curated by the Quest for Orthologs group for benchmarking orthology inference.[139] The species tree was downloaded from SwissTree (https://swisstree.sib.swiss/cgi-bin/swisst). Analysis of UniProt database reviewed proteins annotated with subcellular localizations was performed using the standardized SL accessions (see Zenodo repository), extracted with REST API queries.

Orthology mapping

Protein sequences from each reference FASTA file were searched against the eggNOG 5.0 database[38] and mapped to orthologous groups (OGs) at the rootNOG level (taxonomic level = 1) using eggNOG-mapper v2.0.5[140] with DIAMOND and a hit cut-off e-value of 10^{-3}. As a result, 89,955 unique OGs spanning 156 species across the tree of life were used as input to the Dollo parsimony analysis. The group of rootNOGs assigned to the LECA node as a result of the parsimony procedure were converted to euNOGs (taxonomic level = 2759) with a set of hierarchical mapping files provided by Dr. Jaime Huerta-Cepas, the author of the eggNOG algorithm, via personal correspondence.

Dollo and Wagner parsimony

As no single model is assumed to be correct, instead, we use agreement among complementary reconstruction approaches to define a set of ancestral gene families for downstream biological interpretation. First, using the Count evolutionary analysis software,[141] we implemented a Dollo parsimony approach[39] across 156 organisms to obtain an approximation of the LECA proteome. The Dollo parsimony model relies on the simplifying assumption that gene loss is irreversible, e.g., once a gene is lost it cannot be regained in a lineage. Thus, we determined the ancestral LECA proteome as the set of orthogroups either (a) shared by the respective outgroups (prokaryotes) and at least one of the eukaryotic species or (b) shared by two eukaryotic groups whose last common ancestor was LECA as defined by the gold standard species tree.[142] This approach has previously been shown to be effective at reconstructing likely LECA orthogroups.[19] However, there are caveats to its use, as detailed in the section below titled "Challenges and limitations to defining the LECA gene set". Given these caveats, we additionally used the Count package to implement Wagner parsimony[143] on the same organism/gene set as for Dollo. The Wagner model removes the irreversibility of Dollo gene loss events and allows for both gains and losses, thus accommodating horizontal gene transfer events.

Phylogenetic reconciliation

To complement the Dollo and Wagner parsimony–based inference of ancestral orthogroups, we implemented a large-scale phylogenetic reconciliation pipeline using Notung 3.0[144] to more directly infer gene duplications, losses, and horizontal transfers relative to the reference species tree. We retrieved 10,089 eukaryotic-level gene trees (taxonomic level = 2759) from the eggNOG 5.0 database. For consistency with Notung input requirements, we generated a species-level taxonomic mapping by collapsing strain- and subspecies-level identifiers to their corresponding parent species and renaming each gene-tree leaf with its species mnemonic.

Following preprocessing, each gene tree was subjected to a stepwise Notung workflow consisting of (i) pruning to remove species absent from the reference tree, (ii) duplication–loss (DL) rooting, (iii) DL rearrangement of branches at 70% and 90% support thresholds, (iv) DL-rooting of the rearranged trees, and (v) duplication–transfer–loss (DTL) reconciliation with the reference tree under the "–phylogenomics" mode to infer orthogroup presence or absence across ancestral nodes. All procedures were executed in automated Bash and Python scripts (available on the supporting Github repository); final DTL-reconciled trees and tabulated origin/fam-present summaries are available on the supporting Zenodo repository. The resulting reconciliations yielded two parallel datasets (DTL70, DTL90) that were then summarized to identify orthogroups inferred to originate in LECA and to compare reconciliation-based with Dollo- and Wagner-based ancestral reconstructions. 1,295 (13%) of the OGs were not tested by reconciliation, largely due to species differences between the eggNOG gene trees and the reference species tree.

Investigation of orthologous groups of unknown function

We attempted to assign functions, or, at the minimum, subcellular localizations, to the 25% of uncharacterized (by eggNOG) LECA OGs using the UniProt database. To this end, we downloaded 363,430 proteins from the UniProt database that were (a) "reviewed" status and (b) assigned a standardized subcellular localization (SL) ID by Uniprot for each compartment that we trace back to the last eukaryotic common ancestor (Figure S2A). The total number of proteins assigned to a subcellular compartment varied by four orders of magnitude, where 166,296 proteins were assigned to the cytoplasm at the highest end and 116 proteins were assigned to phagocytic cups at the lowest end (Figure S2A). Furthermore, we investigated the diversity and magnitude of eukaryotic and prokaryotic species contributing to these annotations (Figure S2B) and observed an underrepresentation of clades outside Amorphea (see tree in Figure S5 for supergroup organization). For example, the nucleus is considered a distinguishing feature of eukaryotes; UniProt proteins annotated to localize to the nucleus come predominantly from 1,032 distinct Amorphean species, with almost two orders of magnitude fewer such annotations contributed by non-amorphean species, consisting of 152 archaeplastidans, 2 cryptophytes, 21 excavates, and 62 TSAR species.

To quantify what proportion of the LECA gene set is represented in UniProt, we mapped all 363,420 reviewed UniProt proteins mentioned above to eukaryotic orthologous groups ("euNOGs"; NCBI taxonomic identifier = 2759). This resulted in 13,556 unique euNOGs, the percentage of which that trace back to LECA varies significantly by subcellular localization (Figure S2C). As an aside, we note that the same euNOG can often be assigned multiple UniProt SL IDs, netting a total 24,628 euNOG-SL mappings.

Of the 2,790 ciliary proteins that map to 478 unique eukaryotic orthologous groups (euNOGs), nearly ~25% of the 299 UniProt ciliary euNOGs that intersect with LECA OGs were originally assigned "unknown function" by the eggNOG functional annotation algorithm–a larger proportion than most of the other eukaryotic compartments described within the UniProt database. Similarly, of 1,317 cytoskeletal euNOGs, ~60% trace back to LECA and 111 of those were assigned the "function unknown" eggNOG category. Thus, the combination of eggNOG and UniProt annotations provided a reasonable initial annotation set for subsequent analyses.

Challenges and limitations to defining the LECA gene set

Binning proteins into evolutionarily related orthologous groups with respect to the root of the eukaryotic tree nets a "coarse-grained" mapping of the relationships between eukaryotic genes, *i.e.*, we cannot rigorously distinguish orthologs from paralogs, which could limit the degree to which we can infer functional continuity across homologous gene, as orthologs are argued to be more likely to retain ancestral functions than paralogs (and xenologs), although such trends have generally been weak when measured.[145,146] With that said, we are still able to draw conclusions about the properties of families of genes rather than the pairwise relationships of individual members; this approach is intuitive and convenient for large-scale systematic studies and broadly supported,[147–149] albeit not resolving the phylogenetic relationships among the genes within each orthogroup. However, there is considerable disparity between OG assignment algorithms, though eggNOG has been demonstrated to have among the highest accuracies when tested on a benchmark set of manually curated orthologs.[19] In the same study, the eggNOG algorithm was also shown to perform best at detecting distant homology and properly splitting out-paralogs, making it the best suited algorithm currently available for our goals. Some protein families and eukaryotic lineages with fast rates of evolution (e.g., transcription factors, proteins associated with the innate immune response, and in general plants that are prone to whole genome duplication[150–154]) remain a weakness to the approach. Proteins such as these are likely "under-split" with respect to their associated orthologous groups. Leucine-rich repeat proteins are a salient example: more than 100 human LRR proteins were assigned to KOG0619, an OG we traced back to LECA related to intracellular trafficking and secretion, and this trend persists across nearly all eukaryotes sampled that had proteins assigned to KOG0619. In this way, we are most likely underestimating the size of the distinct LECA gene set.

Table S1 reports the observed support under each method for every LECA OG, so that researchers can check the level of support for specific gene families of interest. Here, we note the strengths and weaknesses of each method contributing to the LECA OG determination. First, the Dollo parsimony procedure used to bound the overall LECA OG set assumes that the probability that a trait emerges more than once is negligible[39] and is the simplest form of ancestral state inference. The use of Dollo parsimony is justified as a base model[155–157] given that (a) our goal was to determine a binary character state (the presence or absence of genes), (b) we had a consensus reference species tree in hand,[138] (c) the target gene set is eukaryotic wherein independent gene losses are common and gains of multiple genes are (relatively) rare, (d) the expected influence of horizontal gene transfer is thought overall to be minimal (estimated to be ~1% of genes or less,[158] but see below), and (e) probabilistic ancestral state reconstruction methods, such as phylogenetic birth-death-gain models, are prohibitively slow for a dataset of this size. Nonetheless, one flaw in this approach is particularly worth noting: multiple species within Excavata host plastids or plastid-derived genes orthologous to plastid proteins in plants,[159–161] even though it is widely accepted that primary plastids share a single origin[162,163] and Archaeplastida is monophyletic.[164,165] If the Archaeplastidan monophyly is to be believed,[166,167] the last common ancestor of Excavata independently acquired plastids (violating Dollo's law), resulting in the inflation of our LECA gene set by ~40 plastid-associated OGs. To correct this error, we manually removed these OGs from consideration during construction of the LECA interactome.

Due to these limitations with Dollo, we applied additional phylogenetic procedures to each of the Dollo-estimated LECA OGs in order to annotate any additional support by these algorithms. Importantly, as the true extent of horizontal gene transfer in early eukaryotes is not well determined, we used methods that considered the possibility of horizontal gene transfer events, Wagner parsimony and duplication-transfer-loss (DTL) phylogenetic reconciliation. Of the 10,091 LECA OGs from Dollo, 6,429 were additionally supported by either Wagner or DTL reconciliation. Notably, an additional 582 OGs were reconciled as Eukaryotes not Excavata. While some of these cases may simply reflect limited taxon sampling in the selected species tree, it is worth noting that a recent tree places the root of the eukaryotic tree within Excavata,[168] and the "Eukaryote not Excavata" OGs may still represent potential LECA OGs. For OGs conserved across only a subset of major clades, changes to the species tree used for reconciliation might thus impact estimates of OG ages, although the most broadly conserved OGs should be reasonably robust to this effect. For the final set of 3,193 OGs present in the estimated LECA interactome, 98% are supported by either Wagner or DTL reconciliation, indicating robust support for their presence in LECA, at least under the caveats discussed here.

Finally, errors in gene trees (as well as reliance on individual estimated gene trees) can degrade performance in reconciliation methods; these methods have also been observed to exhibit higher false positive rates for inferred horizontal gene transfer events as compared to implicit phylogenetic methods such as Count's Wagner parsimony implementation (e.g.,[169]). Hence, these methods can also be subject to age estimation errors, potentially favoring younger ages.

Resources for interactome mapping

Biological samples, mass spectrometry datasets, and software used in this analysis are summarized in the Key Resources Table. Proteomes for 31 eukaryotic species were sourced as summarized in Table S3.

Mass spectrometry

Native protein extraction and fractionation

For lysates described below protease inhibitor cocktail was cOmplete mini EDTA-free (Roche), phosphatase inhibitors were PhosSTOP EASY pack (Roche), and all steps after addition of lysis buffers were conducted at 4°C or on ice unless otherwise indicated. Native soluble extracts were quantified by DC Protein Assay (BioRad). All protein samples were 0.45 μm filtered (Ultrafree-MC-HV Durapore PVDF, Millipore) prior to chromatography. Chromatography was performed on an HPLC system as in[33] unless otherwise stated.

Brachionus rotundiformis was collected in batches on Filter Mesh 100 Nylon (~65 μm pore) to remove feeder algae prior to flash freezing in liquid nitrogen. Frozen material (3.1 g) was ground to power in a liquid nitrogen-chilled mortar and pestle and resuspended in an equal volume of *Tetrahymena* Lysis Buffer (25 mM Tris pH7.4, 25 mM NaCl, 1 mM EDTA, 10% glycerol, 0.2% NP40, with 1 mM DTT, 1 mM PMSF, phosphatase inhibitors, and protease inhibitor cocktail added freshly). Cells were disrupted with 10 strokes in a glass dounce fit with a tight pestle. Following centrifugation 3000 x g, 10 min to remove debris, the supernatant was clarified twice by centrifugation 20,000 x g 10 min. Size Exclusion Chromatography was performed with 2.6 mg extract in a 200 μL sample loop and mobile phase Buffer S (50 mM Tris-HCl pH 7.5, 50 mM NaCl).

Phaeodactylum tricornutum (UTEX 646) grown without silica was briefly washed by pelleting (2000 x g, 10 min, 21°C, no brake) and resuspended in 0.5x artificial seawater (UTEX) before collecting (3000 x g, 4°C, slow deceleration) and flash freezing. Frozen material was ground to powder and allowed to thaw before refreezing and regrinding. 1g of powdered material was resuspended in 800 μL Lysis Buffer (50 mM Tris pH 7.5, 150 mM NaCl 5 mM EGTA, 10% glycerol, 1% NP40 with 0.1mM DTT) with phosphatase inhibitors and Plant Specific Protease Inhibitors (Sigma #P9599). Material was frozen and thawed again before sonicating 6 × 10 s on, 20 s off, 70% duty cycle. Lysis was monitored by microscopy. The extract was incubated on ice with periodic gentle vortexing for 30 min prior to clarification twice at 14,000 x g, 10 min. Extract was diluted 3-fold with 50 mM NaCl prior to loading 2 mg for SEC separation as above. For separation by mixed bed ion-exchange chromatography (Poly CATWAX A, PolyLC Inc.) salt was reduced by 5x dilution with 10 mM Tris pH 7.5, 5% glycerol, 0.01% NaN_3 and proteins were re-concentrated by ultrafiltration (Amicon Ultra 0.5 mL 10,000 MWCO). IEX chromatography was with 1.9 mg in a 250 μL sample loop.

Euglena gracilis (UTEX 753) was washed briefly by centrifugation (1,500 x g, 5 min, 21°C) and resuspension in dH_20, before collection by centrifugation and flash freezing. Material was ground as above, and 4.7 g was resuspended in Lysis buffer plus both the cOmplete mini EDTA-free protease inhibitors and the Plant-Specific Protease Inhibitors (Roche). Lysate was sonicated 9 × 10 s on, 20 s off, 60% duty cycle, followed by gentle nutation 30 min. Debris was removed by centrifugation 1,500 x g, 10 min, and the supernatant was further clarified twice with 14,000 x g, 10 min spins. Final extract was filtered through a 0.45 μm syringe filter (Durapore PVDF, Millipore) prewashed with dH_20. Extract was diluted 4-fold in Buffer S and 2 mg loaded on a 200 μL sample loop for SEC fractionation.

Two fresh pig tracheas (*Sus scrofa*) were shipped on ice from Sierra for Medical Science arriving within 24 h of harvest. After removal of fat tissues the trachea were slit lengthwise, chopped crosswise into several pieces, and washed with multiple changes of ice-cold PBS pH 7.4 to remove serum and blood cells prior to extraction with 100 mL Ca++ shock buffer as in[170] including protease and phosphatase inhibitors at 0.5x concentration and 0.1 mM PMSF. Cilia were released by vortexing and manual agitation for 10 min. Debris was pelleted 500 x g, 2 min and floating lipids were removed by aspiration. Cilia were collected by centrifugation 12,000 x g 10 min and washed once by resuspension and centrifugation. Ciliary pellets were resuspended in Ca++ shock buffer with 1% NP40 to extract soluble proteins and residual axonemes were removed by centrifugation twice at 12,000 x g 10 min. Any floating lipids were removed after each spin. Extract was flash frozen until used. Thawed extract was diluted 2-fold with 10 mM Tris pH 7.5, 5% glycerol, 0.01% NaN_3 and re-clarified 12,000 x g 10 min prior to ultrafiltration with 30,000 MWCO Ultracel Amicon Ultra 0.5 mL units to load 1.7 mg in a 250 μL sample loop for IEX chromatography.

Tetrahymena thermophila SB715 were grown and cilia extracts made as in[171] except that deciliation was by pH shock according to.[172] 1.5 mg cilia extract was fractionated by mixed bed IEX and 1.2 mg by SEC with SEC mobile phase Buffer S-C (50 mM Tris-HCl pH 7.4, 50 mM NaCl, 3 mM $MgSO_4$, 0.1 mM EGTA). Deciliated *Tetrahymena* "bodies" were collected by centrifugation 1,700 x g, 5 min, washed once by resuspension in Deciliation Medium (10 mM Tris-HCl pH 7.4, 10 mM $CaCl_2$, 50 mM sucrose), collected by centrifugation as before and flash frozen until use. *Tetrahymena* body lysate was prepared by liquid nitrogen grinding frozen material before resuspending in an equal volume *Tetrahymena* Lysis Buffer with 0.1 mM PMSF. Lysis was achieved on ice for 10 min by pipetting up and down. Debris was removed by centrifugation 3,000 x g, 10 min. Supernatant was clarified and floating lipids removed by sequential centrifugations at 40,000 x g, 10 min, 45,000 x g 30 min, 130,000 x g 1 h, and 130,000 x g 45 min. Extract was diluted (final NaCl 22 mM) prior to loading 2.2 mg on a 250 μL sample loop for IEX chromatography. The remaining extract was flash frozen and thawed later for SEC chromatography. Extract was clarified 25,000 x g 10 min immediately after thawing, and again after dilution in Buffer S-C for loading of 1.4 mg in 200 μL sample loop. For DSSO-crosslinked samples, cilia extract was prepared using the pH shock method as above, but 20 mM HEPES pH7.4 was substituted for the 50 mM Tris of the Cilia Wash Buffer. Extract was concentrated by ultrafiltration in an Amicon Ultra Ultracel 10k NMWL unit (UFC501096) to load 1.5 mg in a 250 μL sample loop. The final concentration of NP40 was 2.75%. Fractionation on a mixed bed IEX was performed with substitution of 10 mM HEPES pH 7.4 for Tris in the chromatography buffers A and B. For crosslinking DSSO was dissolved freshly in dry DMF to 50 mM and then diluted with 10 mM HEPES pH 7.4 to 10.5 mM before dispensing 25 μL into each 500 μL fraction. To ensure activity of the crosslinker the DSSO solution was prepared in 2 consecutive batches to treat a total of 76 column fractions. Crosslinking proceeded 1 h at room temperature (~21°C) and was quenched by addition of Tris pH 8.0 to 28 mM.

Xenopus laevis sperm were isolated from dissected testes of five or eight J-strain *Xenopus laevis* males. Testes were perforated with a 25-gauge needle, sperm blown out using MMR (Marc's Modified Ringers). Larger debris was allowed to settle, and liquid transferred to a fresh tube. Sperm were collected by centrifugation 1,500 x g, 10 min. Supernatant was discarded and the sperm pellet was lysed by resuspension in an equal volume of Sperm Lysis Buffer (10 mM Tris-HCL pH7.5, 20 mM KCl, 5 mM $MgCl_2$, 5% glycerol, 1% n-Dodecy-ß-D-Maltoside (Anatrace) with 0.5 mM DTT added freshly). Lysate was clarified by centrifugation 14,000 x g, 10 min 1.2 mg was loaded for mixed bed IEX column fractionation (PolyLC Mixed-Bed WAX-WCX, PolyLC Inc. #204CTWX0510), and 3 mg for SEC fractionation (BioSep-SEC-s4000, Phenomenex).

Mus musculus (embryonic stem cells) were grown as described in.[173] Cells were harvested without trypsin by washing in ice-cold phosphate buffered saline (PBS), pelleted, and placed on ice. A 250 μL cell pellet was lysed on ice (5 min) by resuspension in 500 μL of Pierce IP Lysis Buffer (25 mM Tris-HCl pH 7.4, 150 mM NaCl, 1 mM EDTA, 1% NP-40 and 5% glycerol; Thermo Fisher) containing 1x protease inhibitor cocktail III (Calbiochem). During the 5 min, cells were periodically Dounce homogenized with a small-clearance glass pestle (pestle B). Approximately 2 mg of total protein was loaded on either a mixed bed IEX column (PolyLC Mixed-Bed WAX-WCX, PolyLC Inc. #204CTWX0510) or a BioSep-SEC-s4000 gel filtration column (Phenomenex) equilibrated in PBS, pH 7.2. HPLC chromatography was as in[33] and collected fractions were processed as described in.[173]

Lysate preparation and chromatographic separation of other species is described in.[29,33,137,171]

Data acquisition and processing

All column fractions were reduced, alkylated and digested with trypsin for mass spectrometry by either method 1 or 2 of the protocols in.[174] Spectra were collected as in[33] on either a Thermo Scientific Orbitrap Fusion Tribrid or an Orbitrap Fusion Lumos Tribrid mass spectrometer except as noted below. Euglena data were collected on a Lumos using CID (35%) and a topspeed 75 min method as in.[33] Spectra for DSSO crosslinked *Tetrahymena* cilia IEX fractions were collected using a 2 h DDA MS2-MS3 method as described in[171] but processed for protein identifications in this study using the MSBlender pipeline described below.

Experimental analyses of candidate disease genes

Genetic knockouts in Mus musculus

We sourced *Atp6v1a* knockout data (with permission) from the Knockout Mouse Program (KOMP2) sited at the Baylor College of Medicine, which resides under the umbrella of the International Mouse Phenotyping Consortium (IMPC). The IMPC aims to systematically phenotype mice that are homozygous for a single-gene knockout or heterozygous when homozygotes are lethal or sub-viable.[175] Within the IMPC, KOMP2 production centers use the high-throughput and rigorously standardized IMPReSS pipeline to generate and phenotype single-gene knockout mice. Methods for generating single-gene null alleles are described in[176] and phenotype data collection procedures can be accessed in detail at https://www.mousephenotype.org/impress/index.

Atp6v1a mutant alleles were generated by KOMP using CRISPR/Cas9 to introduce a critical exon deletion in a murine C57BL/6N background. Phenotypes were measured from postnatal mice following the embryonic and early adult IMPC pipelines. In the case of *Atp6v1a*, homozygous knockouts resulted in complete penetrance of pre-weaning lethality. As a result, heterozygous knockouts were generated for 8 female mice and 8 male mice. At 14 weeks, bone mineral content and density was measured for each *Atp6v1a*$^{em1(IMPC)Bay/+}$ mutant using dual-energy X-ray absorptiometry (DEXA). The IMPC uses the PhenStat R package to identify abnormal phenotypes from high-throughput pipelines[177]; for the *Atp6v1a*$^{em1(IMPC)Bay/+}$ mutants, a linear mixed model factoring in the effects of sex and body weight was implemented in PhenStat to assess significance of differential bone mineral content.

EFHC2 patient genetics

Individual A4237-22 was a male of Egyptian origin who was diagnosed with small kidneys, increased echogenicity, cortical and medullary cysts, and microcephaly. To identify a potential genetic cause for the individual's phenotype, authors S.S. and F.H. performed whole exome sequencing (WES) analysis on individual A4237-22. Given the parents' unaffected status regarding their renal phenotype, a recessive mode of inheritance was hypothesized.

Homozygosity mapping revealed only 4.4 Mb of homozygosity, confirming the non-consanguinity of the parents (Figure S4A). We detected a hemizygous X-linked missense variant in A4237-22 (c.398G>A; p.Arg133His) (Figures 6D; S4B). The variant has not been reported as homozygous or hemizygous in the gnomAD database in 166,211 control individuals. The p.Arg133His amino acid resides in the DM10 domain (Figure 6E).

The patient's DNA was also screened for potentially deleterious variants in all genes known to cause kidney disease without results.

Protein localization and knockdown experiments in Xenopus laevis

Xenopus embryo manipulations were performed as in.[178–180] Briefly, female adult *Xenopus* were ovulated by injection of hCG (human chorionic gonadotropin). *In vitro* fertilization was carried out by homogenizing a small fraction of a testis in 1/3X Marc's Modified Ringer's (MMR). Embryos were dejellied in 1/3X MMR with 2.5% cysteine (pH 7.8) at the two-cell stage. For microinjections, embryos were placed in a 2% Ficoll and 1/3X MMR solution, injected with mRNA using forceps and an Oxford universal micromanipulator, and washed with 1/3X MMR after 2 h.

The full length sequences of *Xenopus EFHC2* and *GLG1* were downloaded from Xenbase.[181] The DNAs corresponding to the open reading frames (ORFs) of *EFHC2* and *GLG1* were amplified from *Xenopus* cDNA and were cloned into a pCS10R MCC vector containing an N-terminal GFP or a C-terminal FLAG tag driven by an MCC specific alpha tubulin promoter, respectively. The pCS10R MCC *GFP-EFHC2* R133H construct was generated by site-directed mutagenesis (NEB, #E0554S) from pCS10R MCC *GFP-EFHC2*. Capped mRNAs were synthesized using the mMESSAGE mMACHINE SP6 transcription kit (Invitrogen Ambion, #AM1340). A morpholino antisense oligonucleotide (MO) against *GLG1* was designed to block translation (GeneTools). The MO sequence is 5′-CCATCTTGGGAAGTGCTAGTCAAG-3’.

mRNA and MO were injected into two ventral blastomeres of 4-cell stage *Xenopus* embryos in 2% Ficoll (w/v) in 1/3 X MMR and the injected doses of mRNAs or MO per cell are as follows: *GFP-EFHC2* and *GFP-EFHC2* R133H (78 pg), *GFP-IFT56* and *GFP-IFT80* (100 pg),[134] membraneRFP(50 pg), *GLG1-FLAG* for rescue experiment (700 pg), and *GLG1* MO (30 ng) for the knockdown experiment. Live images were captured at stage 23 or stage 25 with LSM700 inverted confocal microscope (Carl Zeiss) with a Plan-APOCHROMAT 63×/1.4 oil immersion objective or Nikon eclipse Ti confocal microscope with 60×/1.4 oil immersion objective.

Imaging analysis was performed using Fiji. Bonferroni-adjusted *p*-values were calculated in R using the base stats package. Unprocessed, uncompressed raw images are available from Mendeley (https://doi.org/10.17632/fnv33t96vn.1).

QUANTIFICATION AND STATISTICAL ANALYSIS

Computational analyses

Reference database construction

Protein sequences from each of the 31 reference FASTA files in Table S3 were compared against the eggNOG 5.0 database[38] and mapped to orthologous groups (OGs) at the euNOG level (taxonomic level = 2759) using eggNOG-mapper v2.0.5[140] with DIAMOND and a hit cut-off e-value of 10^{-3}. Using a previously well-tested strategy for comparative proteomics,[33] for each species, a reference database was constructed where proteins are binned into their respective OGs such that each FASTA entry represents a bin of proteins or protein family; this was accomplished by concatenating each sequence assigned to an OG interposed with a triple lysine sequence. Since we allow for two missed trypsin cleavages in peptide spectra assignment, this triple lysine sequence ensures that we avoid the misassignment of peptides matching a chimera of two binned sequences. The benefits of this approach are 3-fold: (1) defining proteomes in terms of OGs enables cross-species comparisons, (2) OG binning recovers peptide mass spectra that otherwise could not be uniquely assigned to highly sequence-similar proteins, and as a natural extension (3) facilitates proteomic analysis of species with high ploidy, e.g., *X. laevis* (allotetraploid)[137] and *T. aestivum* (allohexaploid).[33]

Peptide mass spectra processing

Matching of mass spectra to peptides was performed with MSGF+, X!Tandem, and Comet-2013.02.0, each run with 10ppm precursor tolerance and allowing for fixed cysteine carbamidomethylation (+57.021464) and optional methionine oxidation (+15.9949). Peptide search results were integrated with MSBlender[182] as described in[29,33] with the exception that high confidence (1% FDR) peptide spectral matches were required from two out of the three peptide identification algorithms. In all, we measured 379,758,411 peptides that were uniquely assigned to 259,732 unique proteins and orthogroups across all fractions. These results were filtered such that we only retain orthogroups that (a) were determined to trace back to the last eukaryotic common ancestor and (b) were strongly observed such that the sum total peptide spectral matches (PSMs) across all fractionations was $\geq$ 150.

Feature curation for protein-protein interactions

For each orthogroup found in each MS fractionation for each species sample, an elution vector was constructed by concatenating the peptide spectral counts for each orthogroup in each fraction. Four measures were used to compare all pairwise elution vectors: the Pearson correlation coefficient, Spearman's correlation coefficient, Euclidean distance, Bray-Curtis dissimilarity. These measures were computed as described in[174] and were generated for: (1) vectors for 149 individual fractionations, (2) concatenated vectors that include all samples within the Amorphea eukaryotic supergroup, (3) concatenated vectors that include all samples within the Excavate eukaryotic supergroup, (4) concatenated vectors that include all samples within the TSAR eukaryotic supergroup, (5) concatenated vectors that include all samples within the Archaeplastida eukaryotic supergroup, and (6) concatenated vectors that include all eukaryotic samples, netting 616 CFMS features.

To specifically target conserved pan-eukaryotic protein interactions, we required elution vectors for each protein-protein interaction (PPI) to have a minimum Pearson *r* of 0.3 and be observed in at least two of the four eukaryotic supergroups, i.e., Amorphea, Excavata, TSAR, and/or Archaeplastida (Figure 2B). This reduced the size of our input data from 17,895,154 pairwise protein comparisons to a curated set of 4,491,719 highly conserved PPIs. Finally, we integrated the intersection of these conserved PPIs with 47 pairwise features generated from an orthogonal collection of ~15,000 mass spectrometry proteomic experiments[30] that include APMS,[54–56] proximity labeling,[57,58] and RNA-pulldown data[59] to attain our final PPI feature matrix, resulting in a total of 663 features for each of 4,491,719 highly conserved potential pairwise PPIs.

Assembly of gold standard protein complexes

Gold standard protein interactions were downloaded from the CORUM[60] (http://mips.helmholtz-muenchen.de/corum) and Complex Portal[61] (https://www.ebi.ac.uk/complexportal) databases. Both databases include protein-protein interactions for multiple species, spanning multiple mammals in CORUM (human, rat, mouse, cow, pig) and many eukaryotes in Complex Portal (human, rat, mouse, cow, pig, yeast, *Arabidopsis*, worm, fly, chicken, snake, fish, frog, rabbit). Redundant complexes were merged, and (to reduce representational bias) any complex with >30 subunits was removed from the gold standard complex set. Finally, UniProt IDs were matched to euNOG IDs, and the gold standard complexes were pruned to only include those in the LECA proteome as determined by the ancestral state reconstruction described above.

Machine learning for protein interactions

All gold standard PPIs observed in our filtered dataset were labeled as positive interactions. Negative interactions were defined as interactions between proteins in different gold standard complexes (e.g., given two gold standard heterotrimers A-B-C and X-Y-Z, data corresponding to an A-X protein pair would be labeled as a negative interaction). To mitigate class imbalance, the total number of negative labels was limited to 3X the observed number of positive PPIs in our data, resulting in 6,629 total positive PPI and 19,887 total negative PPI labels in our feature matrix.

Positive PPIs that participate in multiple complexes are a potential source of representation bias in the truth set, which can lead to under or overfitting during model training. To overcome this, we implemented a data stratification approach (Figure S6). First, all gold standard protein complexes are given a unique numeric ID. All protein pairs within a complex inherit that ID. Negative PPIs receive

group labels by randomly sampling the distribution of positive PPI group IDs with replacement. If a protein pair participates in >1 complex, that pair will be labeled with a list of IDs. These ID lists represent networks of overlapping complexes. We implemented transitive closure of the networks by recursively merging ID lists that overlap with each other, netting a fully stratified "supergroup" label for each gold standard PPI in the dataset.

A group-based split method (scikit-learn's "GroupShuffleSplit" class) was used to generate 5 sets of test and training data, where 75% of the labeled data was used for training and 25% for testing. Each of the 5 training sets were used as input into TPOT,[183] an automated machine learning pipeline built on top of scikit-learn, to find the best classification method, pre-processing steps, and parameters for our data. While TPOT generates an internal cross-validation score to evaluate the performance of different models, the pipeline is agnostic to strata within the training set and is thus subject to overfitting. In each instance, the 25% hold-out set was used as a true test of the optimized models produced by TPOT. These results are reported in Table S4. In the majority of cases, TPOT reports the extremely randomized trees (ExtraTrees) algorithm with slightly varying parameters and pre-processing steps as the best model for our data. However, both a stochastic gradient descent and linear support vector classification (SGDClassifier and LinearSVC, respectively) pipeline scored comparably to the ExtraTrees method, so we moved forward with feature selection and model assessment for those pipelines as well.

To further reduce the risk of overfitting, recursive feature elimination with cross-validation (RFECV) was used to obtain feature importances and an optimal feature set for each of the chosen models (see Zenodo data repository). Feature importance was evaluated using either the Gini index (in the case of ExtraTrees) or the absolute value of the coefficients of the linear model (in the case of LinearSVC and SGDClassifier). While RFECV generates an internal cross-validated test score to determine an optimal feature set, the module is agnostic to stratified data and is thus subject to sampling bias. Again, we employed a custom group-based split approach (scikit-learn's "GroupKFold" class, illustrated in Figure S7) to generate 5 sets of test and training data. Since GroupKFold generates test/train splits such that every protein complex supergroup is included in the test data at least once, this allows us to use hold-out test sets to gauge bias in the "best" feature sets output by RFECV while also evaluating feature importance stability.

RFECV determines an optimal feature set following these steps: (1) the input training data is used to fit a given model, resulting in either a Gini index value or coefficient for each feature in the dataset; (2) the least important feature(s) are recursively removed and the mean test accuracy is computed using cross-validation across the input training set, (3) the optimal number of features is determined such that mean test accuracy is maximized. Then, we use the holdout test set to evaluate the true performance of the final model as output by RFECV method. True feature importance was assessed by aggregating the RFECV results of each split, and, for each feature, counting the number of times it appears in the "optimal" set output and computing the mean and relative standard deviation of its Gini index/coefficient. The "best" features are those that maximize the number of appearances in the final "optimal" set across GroupKFolds splits, maximize the absolute value of the Gini index/coefficient and minimize relative standard deviation (*i.e.*, low RSD indicates the feature importance is stable across GroupKFold train/test splits).

We measured precision and recall for each model using the top 5, 10, 25, 50, 100, and 250 highest ranked features (determined on a model-by-model basis, in other words, the "top" features for the LinearSVC classifier are different than the "top" features for the ExtraTreesClassifier) and all 663 features (Figure S8A), choosing the feature set that had the highest number of PPIs and unique proteins within a 10% FDR threshold (Figures S8B and S8C).

Community detection of protein complexes

Communities of interacting proteins were identified using the walktrap algorithm via the igraph library interface in Python. Briefly, the walktrap algorithm detects community structure by executing a user-defined number of random walks from each vertex in a graph. Random walks tend to become "trapped" in strongly connected sub-communities of the graph,[62] a behavior that we reinforce by weighting graph edges with the probability scores output by our three PPI models (LinearSVC, ExtraTrees, and SGDClassifier). Then, individual protein complex clusters are partitioned such that the modularity of the network is maximized,[184] netting an "optimal" number of protein complex communities given the input graph structure. The walktrap algorithm performed best with the PPI scores output by the LinearSVC model, and was thus chosen as our final interactome. The features used in the final model are reported in the Zenodo repository.

Validation of the protein interaction model with external data

External interaction datasets were sourced from.[63–65] The likelihood of LECA PPIs (I_L) agreeing with external interaction networks (I_E) was calculated with a formula analogous to an odds ratio, described by the equations below.

		LECA interaction (I_L)	
		+	-
**External interaction (I_E)**	+	A	B
	-	C	D

$$A = I_E \cap I_L \qquad \text{(Equation 1)}$$

$$B = I_E \setminus I_L \quad \text{(Equation 2)}$$

$$C = I_L \setminus I_E \quad \text{(Equation 3)}$$

$$D = I \notin (I_E \cup I_L) \quad \text{(Equation 4)}$$

$$A + B + C + D = \frac{N_i(N_i - 1)}{2} \quad \text{(Equation 5)}$$

$$\frac{P(I_{L+}|I_{E+})}{P(I_{L+}|I_{E-})} = \frac{A/(A+B)}{C/(C+D)} = \frac{A(C+D)}{C(A+B)} \quad \text{(Equation 6)}$$

Challenges and limitations to mapping the LECA protein interaction set

It is important to note that the data used in this study strongly favors humans and mammals in general. Most of the CFMS and APMS experiments in this study are sourced from humans. Of the 25,000 experiments included in this study, approximately 18,000 are derived from human cells. Gold standard protein complexes obtained from the CORUM database are exclusively mammalian, which motivated us to also incorporate gold standard PPIs from the ComplexPortal database. However, though ComplexPortal is more diverse and includes *Arabidopsis* assemblies, most of the data is still Amorphean. We took a number of steps to ensure we target pan-eukaryotic proteins and protein interactions; for example, we filtered the MS data to only include proteins that we trace back to LECA as well as require every PPI be observed and reasonably correlated by CFMS (Pearson $r > 0.3$) in at least 2 of the 4 eukaryotic supergroups (as defined in Figure 2B) prior to entry into the machine learning pipeline, with this latter requirement helping to ensure that PPI support is not drawn solely from opisthokonts.

Ideally, it would be preferable to experimentally determine interactions independently in each species and use these as traits for evolutionary analysis. In practice, however, such an approach is deeply underpowered, as all current protein interaction mapping technologies, including CF-MS, affinity-purification/MS, immunoprecipitation/MS, yeast 2-hybrid, and even computational approaches such as AlphaFold, exhibit high false negative rates for detecting and measuring protein interactions.[185] This tendency is exacerbated further by the limited sampling of tissues and cell types imposed by a study of this scale; hence a full evolutionary analysis is not sufficiently powered to be effective with current datasets. This limitation motivates the current (better powered) approach to determining interactions as accurately as possible using the pooled data across species, while requiring supporting evidence from multiple supergroups to ensure a high probability of the interactions being ancestral.

Co-fractionation mass spectrometry identifies stable complexes that survive biochemical fractionation. Stochastic sampling across a large number of species and fractionations allows us to recover some transient interactions. CFMS is also biased toward abundant and soluble proteins, though we typically employ detergents to improve coverage of membrane proteins. We measure approximately 60% of the 10,091 LECA OGs (probably due to the above detection biases) and high precision PPIs for half of these, indicating a high false negative rate. Inclusion of the APMS datasets increases the power of our model for PPI detection but only for systems conserved within Amorphea. Binning proteins into evolutionarily related protein families (orthogroups) prior to peptide identification and assignment results in loss of resolution for different isoforms and some paralogs. For the most part, we are able to draw conclusions about the properties of families of genes rather than individual members. In some cases, we can retroactively disentangle which variant(s) or paralog(s) participate in a specific interaction by examining the specific peptides identified by mass spectrometry. Moreover, the loss of family member resolution is offset by improved protein identifications: Since the proteomes must be defined in terms of OGs in any event in order to effectively perform cross-species comparisons, by moving that process to the protein identification step, we additionally benefit from the increased protein identification rates by allowing orthogroup-restricted peptides to contribute to identification and quantification, significantly improving proteome coverage (see[33] for quantification of this effect).

Resources for disease analyses

We downloaded 17,019 gene-disease relationships from the Online Mendelian Inheritance in Man database (omim.org).[119]

Curation of a gene-disease dataset

Ensembl accessions from the OMIM dataset were first mapped to human UniProt identifiers and then subsequently matched with their corresponding eggNOG orthologous groups (OGs) at the root of eukaryotes (NCBI taxonomy level = 2759). Gene-disease assignments in the raw OMIM data did not follow a standardized scheme and required a combination of programmatic and manual cleaning. For example, AKT1, PTEN, KLLN, and SEC23B are respectively assigned to Cowden syndrome 6, Cowden syndrome 1, Cowden syndrome 4, and Cowden syndrome 7 in the original dataset. After cleaning, these genes are grouped under a common "Cowden syndrome" label. We filtered the dataset to contain only LECA OGs, netting 5,761 genotype-to-phenotype associations for the network propagation of gene-disease relationships in the conserved eukaryotic interactome. In total, we curated 1,683 unique disease labels for 2,262 highly conserved human genes.

Network propagation

We used a cross-validated network propagation approach to systematically assign disease predictions to proteins in the interactome. If a protein in the network is known to be associated with a disease, then each connecting node receives the score of the

connecting edge. This process is repeated for each unique disease label in our OMIM dataset given that the disease has at least five mapped associations with genes that also map to an orthologous group in the last eukaryotic common ancestor. To assess the quality of this propagation for each disease, we iteratively leave out true positive nodes and query how well the propagation recapitulates known gene-disease relationships (*i.e.*, leave-one-out cross validation). We calculate true and false positive rates to construct a receiver operating characteristic (ROC) curve as a function of propagated score. Then, we use the area under the ROC curve (AUROC) as a measure of performance. Additionally, for each disease, we repeat propagation from randomly selected nodes from the network to evaluate the statistical strength of the gene-disease network versus randomly assigned gene-disease relationships.

Analysis of yeast gene-mutant phenotype datasets

We downloaded results from 14,484 phenotypic screens across 4,554 mutant yeast strains via the YeastPhenome project.[186] Normalized phenotypic values (NPVs) are used in the YeastPhenome dataset to measure gene-phenotype associations. NPVs greater than 3 standard deviations (s.d.) above the mutant strain mean or less than −3 s.d. below the mean denote significant gene-phenotype associations. This dataset was split by NPVs into associations with NPVs greater than or equal to 3 (“upper”) and less than or equal to −3 (“lower”). This resulted in 226,050 upper gene-phenotype associations and 458,562 lower gene-phenotype associations, netting 684,612 significant associations in yeast across 19,607 phenotypes. The genes for these associations were matched to their corresponding LECA OGs, and these gene sets for each yeast mutant phenotype analyzed via network propagation in the LECA interactome as described above for the OMIM gene sets.

Analysis of Chlamydomonas gene-mutant phenotype datasets

We downloaded reported phenotypic measurements for 747 *Chlamydomonas* mutant strains[187] and selected all 1,636 significant (FDR <0.3) gene-phenotype associations, spanning 167 unique phenotypes. Assayed genes were matched to their corresponding LECA OGs, then the gene sets for each *Chlamydomonas* phenotype analyzed via network propagation in the LECA interactome as described above for the OMIM gene sets.

Cell Genomics, Volume *6*

Supplemental information

A protein interactome for the last eukaryotic common ancestor illuminates the biochemical basis of modern genetic diseases

Rachael M. Cox, Ophelia Papoulas, Shirlee Shril, Chanjae Lee, Tynan P. Gardner, Zoya T. Ansari, Anna M. Battenhouse, Muyoung Lee, Kevin Drew, Claire D. McWhite, David Yang, Janelle C. Leggere, Dannie Durand, Friedhelm Hildebrandt, John B. Wallingford, and Edward M. Marcotte

SUPPLEMENTAL FIGURES AND LEGENDS

Figure S1. Support for LECA OGs from Wagner parsimony and phylogenetic reconciliation, Related to Figure 1. All OGs presented are supported as present in LECA by at least Dollo parsimony. We further looked for agreement among multiple independent approaches as a robustness test; pathway and gene-level interpretations in the paper are restricted to the multiply-supported subset, with detailed gene-level results provided in **Table S1. (A)** 64% of all LECA OGs supported by Dollo Parsimony are also supported by Wagner or phylogenetic reconciliation with transfers. **(B)** 98% of the core set of 3,193 OGs in the LECA interactome are also supported by Wagner or phylogenetic reconciliation with transfers. For both plots: blue, LECA OGs also supported by Wagner and/or phylogenetic reconciliation with transfers; dark gray, LECA OGs only supported by Dollo parsimony; orange, LECA OGs supported by Dollo parsimony for which phylogenetic reconciliation was not calculated; yellow, LECA OGs supported by Dollo and reconciled as Eukaryotes not Excavata.

Figure S2. Phylogenetic analysis of the reviewed UniProt database by subcellular localization, Related to Figure 1. Limitations to available annotations are evident in an analysis of the UniProt protein database across species, where reviewed proteins have assigned subcellular localizations likely present in the last eukaryotic common ancestor. **(A)** Light gray, total number of reviewed UniProt proteins by UniProt SL term; dark gray, total number of unique eukaryotic OGs assigned to UniProt proteins by UniProt SL term. **(B)** Phylogenetic representation of the proteins sourced from UniProt by UniProt SL term. **(C)** The percentage of eukaryotic orthologous groups (euNOGs) that trace back to LECA by UniProt SL term.

Figure S3. Homozygosity mapping and verification of EFHC2 mutation in individual A4237-22, Related to Figure 6. (A) Homozygosity mapping depicts a homozygosity of 4.4 Mb and confirms the reported non-consanguinity of the parents. **(B)** Chromatograms obtained by direct sequencing of PCR products reveal a homozygous substitution of C for T in exon 4 of the *EFHC2* gene in A4237-22.

Figure S4. Guilt-by-association in the LECA interactome predicts loss-of-function phenotypes in mutant (A,B) yeast and (C) *Chlamydomonas*, Related to Figure 7. Performance in panels **A** and **C** was quantified using the area under receiver operating characteristic curves (AUROC) for leave-one-out cross-validated predictions of known phenotype-linked genes (light blue) versus random associations (yellow), calculated as for Figure 7A and with predictive performance being roughly comparable to the prediction of the human disease gene sets in Figure 7A. Panel **(B)** shows the relevant LECA PPI networks for genes associated with the two labeled yeast phenotypes.

Figure S5. Reference species tree illustration generated by the Interactive Tree of Life for most of the Quest for Orthologs benchmark species (147/156) used in the Dollo and Wagner parsimony analyses, Related to STAR Methods. Branch lengths are not to scale. Major supergroups are highlighted across the tree. Prokaryotic groups include Bacteria (gray) and Archaea (yellow). Eukaryotic groups include Excavata (light blue), Archaeplastida (green), TSAR (purple), and Amorphea (red).

Figure S6. Illustration of transitive closure for grouping gold standard protein complexes into supergroups, Related to STAR Methods.

Figure S7. Illustration of group-based k-fold (in this example, k=3) cross-validation for protein-protein interactions, Related to STAR Methods.

Figure S8. Model selection and optimization, Related to STAR Methods. (A) Precision-recall curves for three different algorithms, varying the number of “top” most important features used as input (duplicate panel to Figure 3B). Feature importance is defined per algorithm, ranked by either the absolute value of coefficients for linear models (LinearSVC, SGDClassifier) or the Gini index (ExtraTreesClassifier). (B) The number of pairwise protein-protein interactions (PPIs) within a 10% FDR threshold for each model. Black stars (★) denote the final models used as input to a community detection algorithm to define protein complexes. (C) The number of unique proteins that have at least one interaction scored within a 10% FDR threshold for each model.

Figure S1.

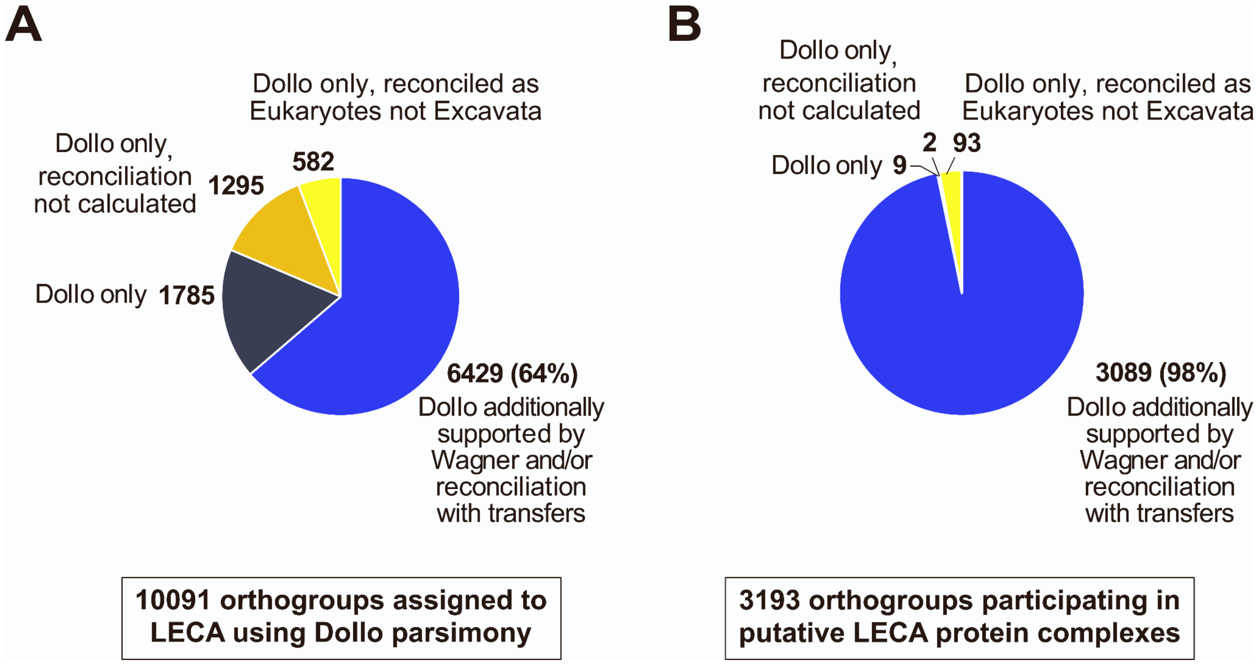
A
Dollo only, reconciled as Eukaryotes not Excavata
Dollo only, reconciliation not calculated
1295
582
Dollo only 1785
6429 (64%)
Dollo additionally supported by Wagner and/or reconciliation with transfers
10091 orthogroups assigned to LECA using Dollo parsimony
B
Dollo only, reconciliation not calculated
Dollo only, reconciled as Eukaryotes not Excavata
2
93
Dollo only 9
3089 (98%)
Dollo additionally supported by Wagner and/or reconciliation with transfers
3193 orthogroups participating in putative LECA protein complexes

Figure S2.

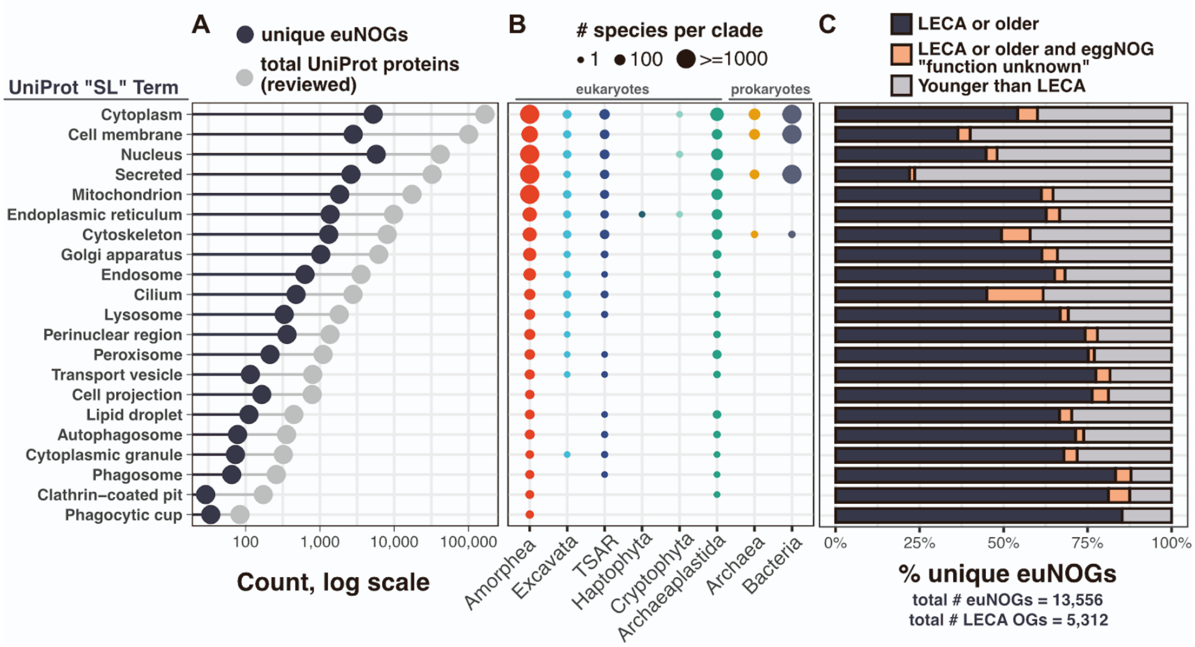
A
unique euNOGs
total UniProt proteins (reviewed)
UniProt "SL" Term
Cytoplasm
Cell membrane
Nucleus
Secreted
Mitochondrion
Endoplasmic reticulum
Cytoskeleton
Golgi apparatus
Endosome
Cilium
Lysosome
Perinuclear region
Peroxisome
Transport vesicle
Cell projection
Lipid droplet
Autophagosome
Cytoplasmic granule
Phagosome
Clathrin–coated pit
Phagocytic cup
100
1,000
10,000
100,000
Count, log scale
B
species per clade
1
100
>=1000
eukaryotes
prokaryotes
Amorphea
Excavata
TSAR
Haptophyta
Cryptophyta
Archaeaplastida
Archaea
Bacteria
C
LECA or older
LECA or older and eggNOG "function unknown"
Younger than LECA
0%
25%
50%
75%
100%
% unique euNOGs
total # euNOGs = 13,556
total # LECA OGs = 5,312

Figure S3.

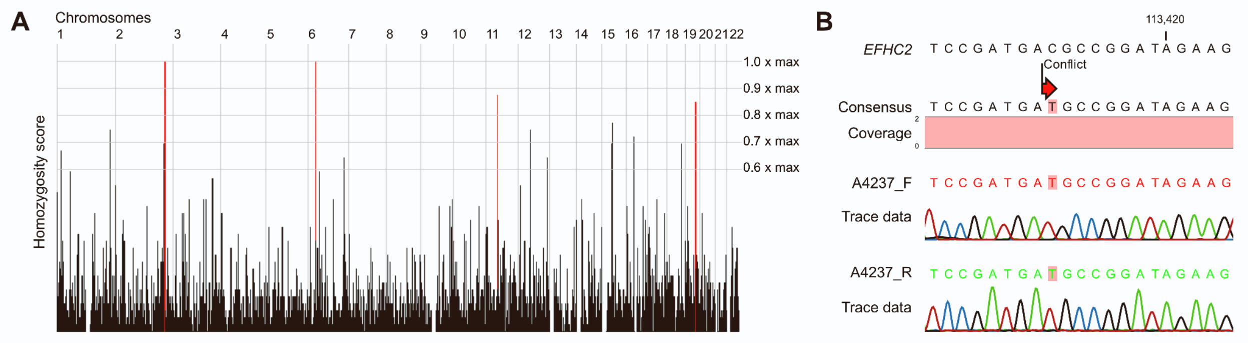
A
Chromosomes
1 2 3 4 5 6 7 8 9 10 11 12 13 14 15 16 17 18 19 20 21 22
1.0 x max
0.9 x max
0.8 x max
0.7 x max
0.6 x max
Homozygosity score
B
113,420
EFHC2
T C C G A T G A C G C C G G A T A G A A G
Conflict
Consensus
T C C G A T G A T G C C G G A T A G A A G
Coverage
A4237_F
T C C G A T G A T G C C G G A T A G A A G
Trace data
A4237_R
T C C G A T G A T G C C G G A T A G A A G
Trace data

Figure S4.

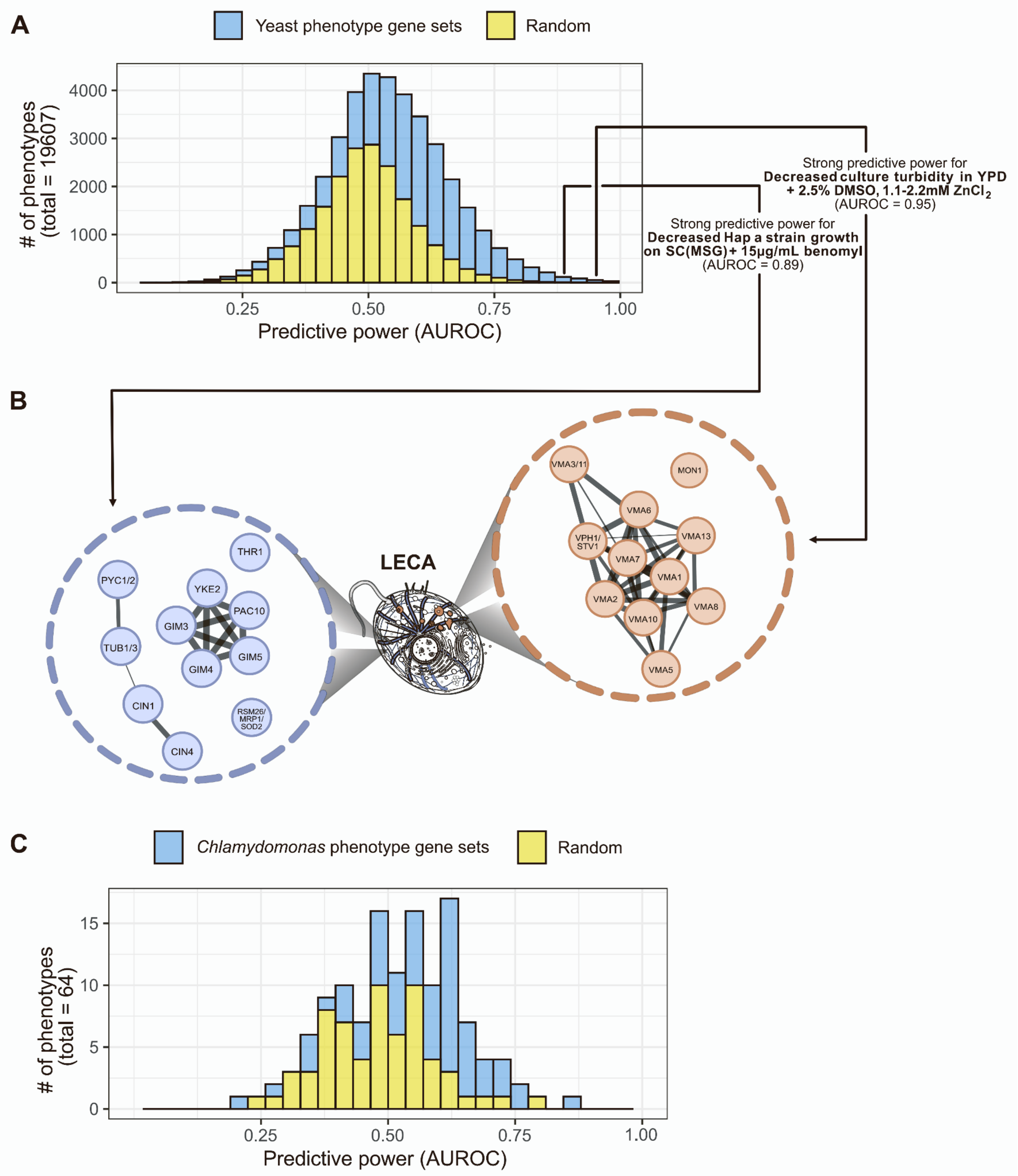

A
Yeast phenotype gene sets
Random
# of phenotypes (total = 19607)
4000
3000
2000
1000
0
0.25
0.50
0.75
1.00
Predictive power (AUROC)
Strong predictive power for
Decreased culture turbidity in YPD + 2.5% DMSO, 1.1-2.2mM $ZnCl_2$
(AUROC = 0.95)
Strong predictive power for
Decreased Hap a strain growth on SC(MSG) + 15μg/mL benomyl
(AUROC = 0.89)
B
LECA
PYC1/2
TUB1/3
CIN1
CIN4
THR1
YKE2
PAC10
GIM3
GIM5
GIM4
RSM26/ MRP1/ SOD2
VMA3/11
MON1
VMA6
VPH1/ STV1
VMA13
VMA7
VMA1
VMA2
VMA10
VMA8
VMA5
C
Chlamydomonas phenotype gene sets
Random
# of phenotypes (total = 64)
15
10
5
0
0.25
0.50
0.75
1.00
Predictive power (AUROC)

Figure S5.

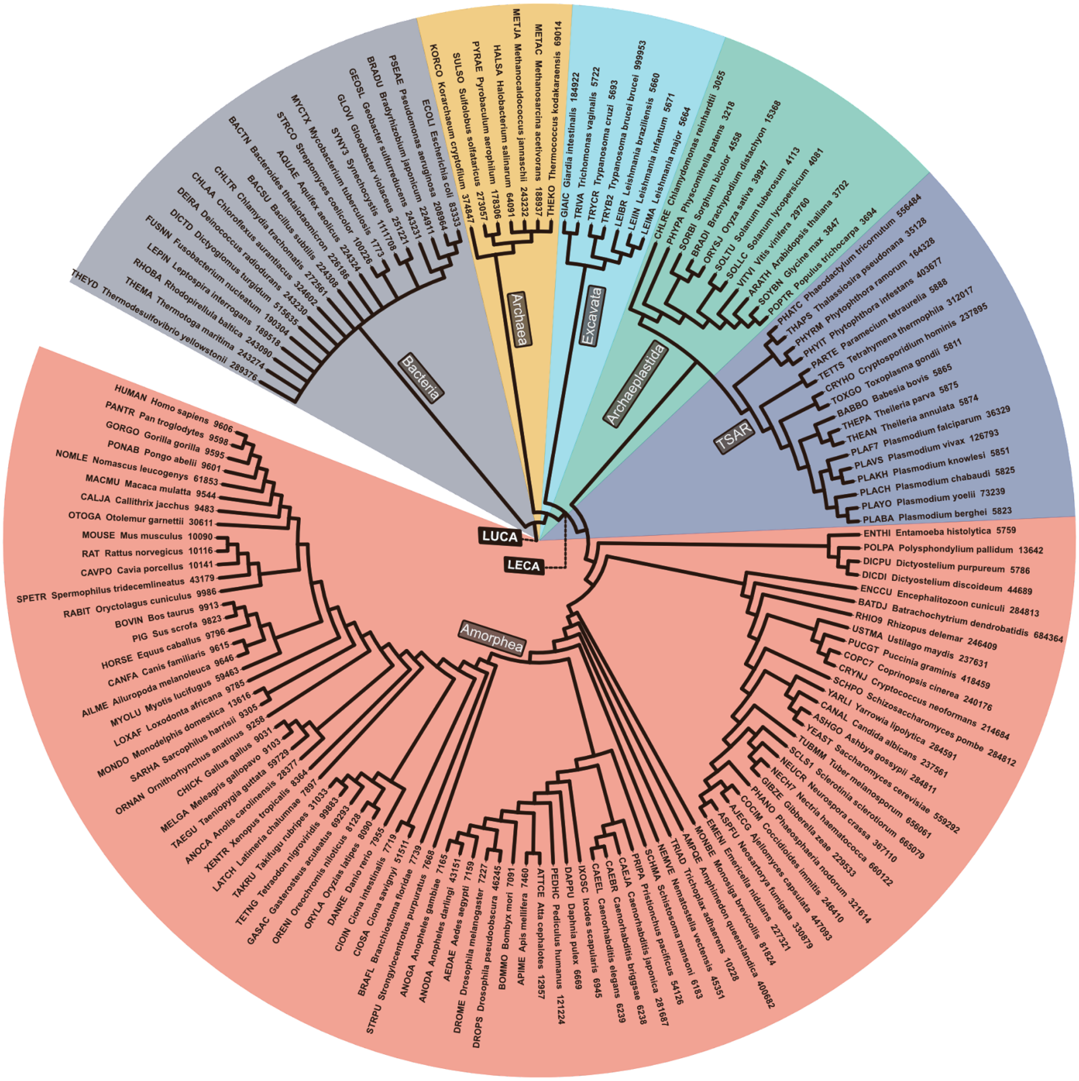
LUCA
LECA
Bacteria
Archaea
Excavata
Archaeplastida
TSAR
Amorphea
THEYD Thermodesulfovibrio yellowstonii 289376
THEMA Thermotoga maritima 243274
RHOBA Rhodopirellula baltica 243090
LEPIN Leptospira interrogans 189518
FUSNN Fusobacterium nucleatum 190304
DICTD Dictyoglomus turgidum 515635
DEIRA Deinococcus radiodurans 243230
CHLAA Chloroflexus aurantiacus 324602
CHLTR Chlamydia trachomatis 272561
BACSU Bacillus subtilis 224308
BACTN Bacteroides thetaiotaomicron 226186
AQUAE Aquifex aeolicus 224324
STRCO Streptomyces coelicolor 100226
MYCTX Mycobacterium tuberculosis 1773
SYNY3 Synechocystis 1111708
GLOVI Gloeobacter violaceus 251221
GEOSL Geobacter sulfurreducens 243231
BRADU Bradyrhizobium japonicum 224911
PSEAE Pseudomonas aeruginosa 208964
ECOLI Escherichia coli 83333
KORCO Korarchaeum cryptofilum 374847
SULSO Sulfolobus solfataricus 273057
PYRAE Pyrobaculum aerophilum 178306
HALSA Halobacterium salinarum 64091
METJA Methanocaldococcus jannaschii 243232
METAC Methanosarcina acetivorans 188937
THEKO Thermococcus kodakarensis 69014
GIAIC Giardia intestinalis 184922
TRIVA Trichomonas vaginalis 5722
TRYCR Trypanosoma cruzi 5693
TRYB2 Trypanosoma brucei brucei 999953
LEIBR Leishmania braziliensis 5660
LEIIN Leishmania infantum 5671
LEIMA Leishmania major 5664
CHLRE Chlamydomonas reinhardtii 3055
PHYPA Physcomitrella patens 3218
SORBI Sorghum bicolor 4558
BRADI Brachypodium distachyon 15368
ORYSJ Oryza sativa 39947
SOLTU Solanum tuberosum 4113
SOLLC Solanum lycopersicum 4081
VITVI Vitis vinifera 29760
ARATH Arabidopsis thaliana 3702
SOYBN Glycine max 3847
POPTR Populus trichocarpa 3694
PHATC Phaeodactylum tricornutum 556484
THAPS Thalassiosira pseudonana 35128
PHYRM Phytophthora ramorum 164328
PHYIT Phytophthora infestans 403677
PARTE Paramecium tetraurelia 5888
TETTS Tetrahymena thermophila 312017
CRYHO Cryptosporidium hominis 237895
TOXGO Toxoplasma gondii 5811
BABBO Babesia bovis 5865
THEPA Theileria parva 5875
THEAN Theileria annulata 5874
PLAF7 Plasmodium falciparum 36329
PLAVS Plasmodium vivax 126793
PLAKH Plasmodium knowlesi 5851
PLACH Plasmodium chabaudi 5825
PLAYO Plasmodium yoelii 73239
PLABA Plasmodium berghei 5823
ENTHI Entamoeba histolytica 5759
POLPA Polysphondylium pallidum 13642
DICPU Dictyostelium purpureum 5786
DICDI Dictyostelium discoideum 44689
ENCCU Encephalitozoon cuniculi 284813
BATDJ Batrachochytrium dendrobatidis 684364
RHIO9 Rhizopus delemar 246409
USTMA Ustilago maydis 237631
PUCGT Puccinia graminis 418459
COPC7 Coprinopsis cinerea 240176
CRYNJ Cryptococcus neoformans 214684
SCHPO Schizosaccharomyces pombe 284812
YARLI Yarrowia lipolytica 284591
CANAL Candida albicans 237561
ASHGO Ashbya gossypii 284811
YEAST Saccharomyces cerevisiae 559292
TUBMM Tuber melanosporum 656061
SCLS1 Sclerotinia sclerotiorum 665079
NEUCR Neurospora crassa 367110
NECH7 Nectria haematococca 660122
GIBZE Gibberella zeae 229533
PHANO Phaeosphaeria nodorum 321614
COCIM Coccidioides immitis 246410
AJECG Ajellomyces capsulatus 447093
ASPFU Neosartorya fumigata 330879
EMENI Emericella nidulans 227321
MONBE Monosiga brevicollis 81824
AMPQE Amphimedon queenslandica 400682
TRIAD Trichoplax adhaerens 10228
NEMVE Nematostella vectensis 45351
SCHMA Schistosoma mansoni 6183
PRIPA Pristionchus pacificus 54126
CAEJA Caenorhabditis japonica 281687
CAEBR Caenorhabditis briggsae 6238
CAEEL Caenorhabditis elegans 6239
IXOSC Ixodes scapularis 6945
DAPPU Daphnia pulex 6669
PEDHC Pediculus humanus 121224
ATTCE Atta cephalotes 12957
APIME Apis mellifera 7460
BOMMO Bombyx mori 7091
DROPS Drosophila pseudoobscura 46245
DROME Drosophila melanogaster 7227
AEDAE Aedes aegypti 7159
ANODA Anopheles darlingi 43151
ANOGA Anopheles gambiae 7165
STRPU Strongylocentrotus purpuratus 7668
BRAFL Branchiostoma floridae 7739
CIOSA Ciona savignyi 51511
CIOIN Ciona intestinalis 7719
DANRE Danio rerio 7955
ORYLA Oryzias latipes 8090
ORENI Oreochromis niloticus 8128
GASAC Gasterosteus aculeatus 69293
TETNG Tetraodon nigroviridis 99883
TAKRU Takifugu rubripes 31033
LATCH Latimeria chalumnae 7897
XENTR Xenopus tropicalis 8364
ANOCA Anolis carolinensis 28377
TAEGU Taeniopygia guttata 59729
MELGA Meleagris gallopavo 9103
CHICK Gallus gallus 9031
ORNAN Ornithorhynchus anatinus 9258
SARHA Sarcophilus harrisii 9305
MONDO Monodelphis domestica 13616
LOXAF Loxodonta africana 9785
MYOLU Myotis lucifugus 59463
AILME Ailuropoda melanoleuca 9646
CANFA Canis familiaris 9615
HORSE Equus caballus 9796
PIG Sus scrofa 9823
BOVIN Bos taurus 9913
RABIT Oryctolagus cuniculus 9986
SPETR Spermophilus tridecemlineatus 43179
CAVPO Cavia porcellus 10141
RAT Rattus norvegicus 10116
MOUSE Mus musculus 10090
OTOGA Otolemur garnettii 30611
CALJA Callithrix jacchus 9483
MACMU Macaca mulatta 9544
NOMLE Nomascus leucogenys 61853
PONAB Pongo abelii 9601
GORGO Gorilla gorilla 9595
PANTR Pan troglodytes 9598
HUMAN Homo sapiens 9606

Figure S6.

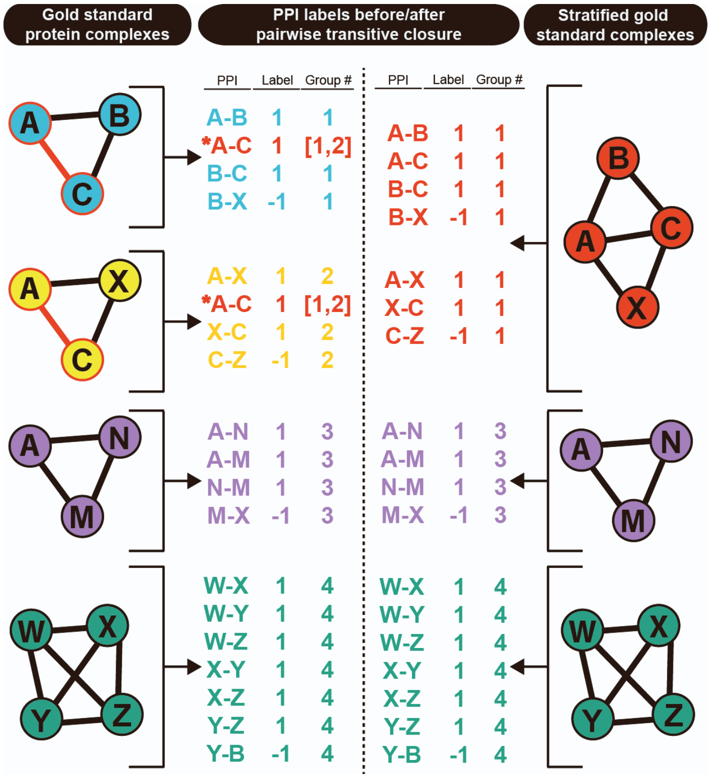

Gold standard protein complexes
PPI labels before/after pairwise transitive closure
Stratified gold standard complexes
PPI Label Group #
A-B 1 1
*A-C 1 [1,2]
B-C 1 1
B-X -1 1
A-X 1 2
*A-C 1 [1,2]
X-C 1 2
C-Z -1 2
A-N 1 3
A-M 1 3
N-M 1 3
M-X -1 3
W-X 1 4
W-Y 1 4
W-Z 1 4
X-Y 1 4
X-Z 1 4
Y-Z 1 4
Y-B -1 4
PPI Label Group #
A-B 1 1
A-C 1 1
B-C 1 1
B-X -1 1
A-X 1 1
X-C 1 1
C-Z -1 1
A-N 1 3
A-M 1 3
N-M 1 3
M-X -1 3
W-X 1 4
W-Y 1 4
W-Z 1 4
X-Y 1 4
X-Z 1 4
Y-Z 1 4
Y-B -1 4

Figure S7.

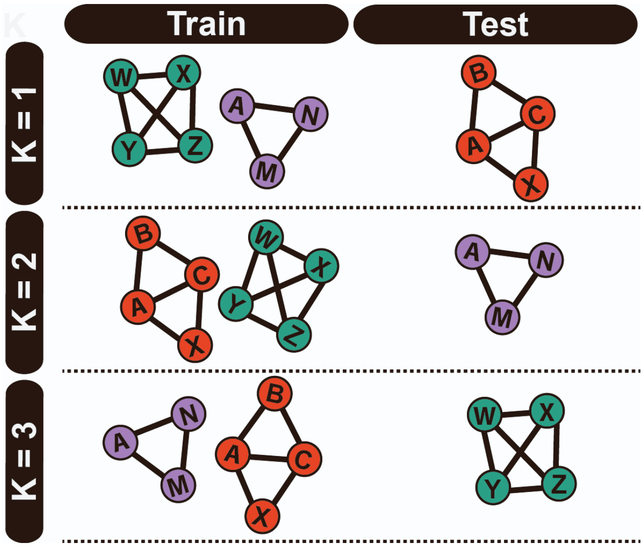

Figure S8.

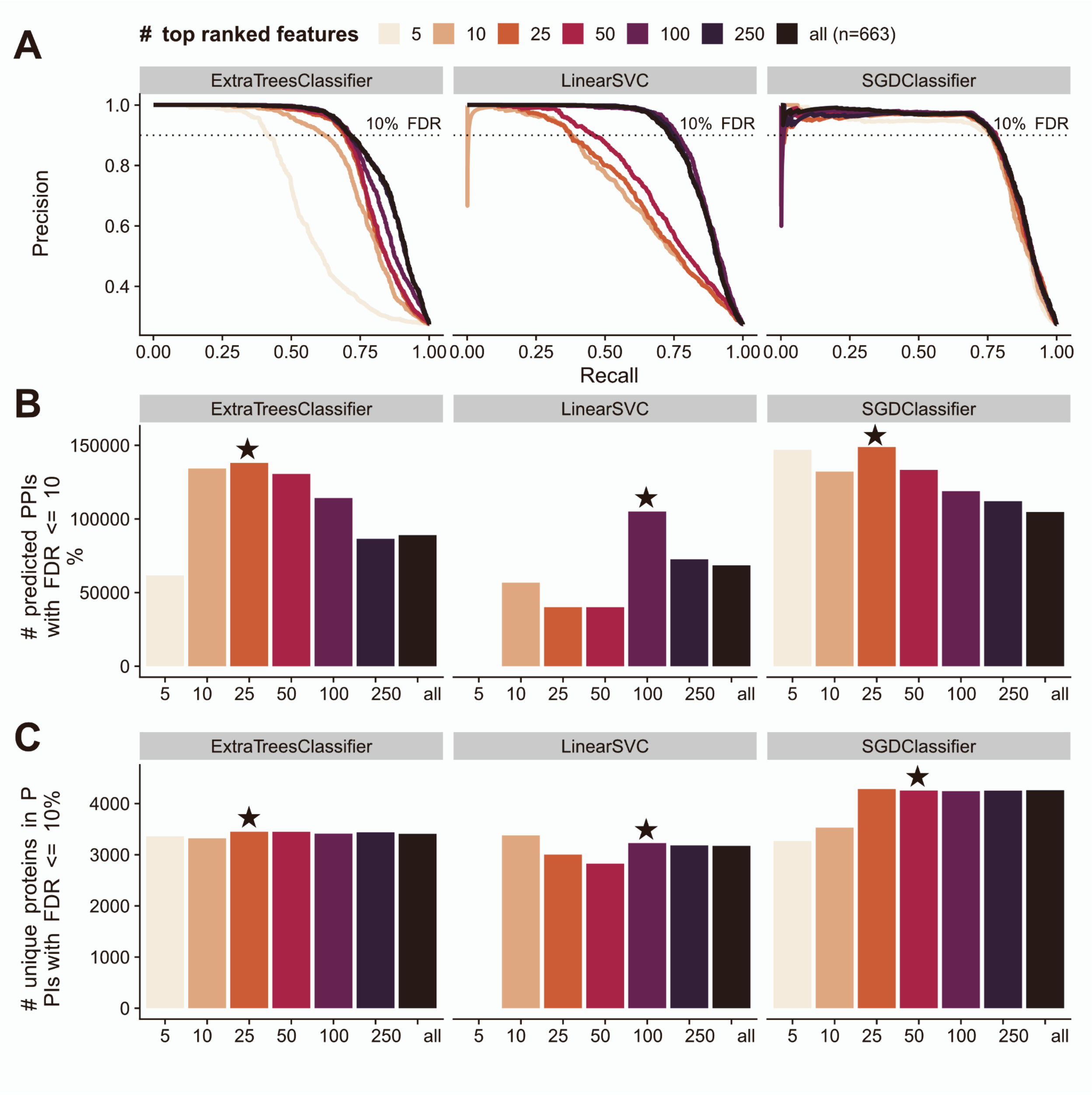
A
top ranked features
5
10
25
50
100
250
all (n=663)
ExtraTreesClassifier
LinearSVC
SGDClassifier
10% FDR
Precision
Recall
B
predicted PPIs with FDR <= 10 %
C
unique proteins in PPIs with FDR <= 10%
